# Discovery of new Schiff bases of the disalicylic acid scaffold as DNA gyrase and topoisomerase IV inhibitors endowed with antibacterial properties

**DOI:** 10.3389/fchem.2024.1419242

**Published:** 2024-06-07

**Authors:** Lamya H. Al-Wahaibi, Mohamed A. Mahmoud, Hayat Ali Alzahrani, Hesham A. Abou-Zied, Hesham A. M. Gomaa, Bahaa G. M. Youssif, Stefan Bräse, Safwat M. Rabea

**Affiliations:** ^1^ Department of Chemistry, College of Sciences, Princess Nourah Bint Abdulrahman University, Riyadh, Saudi Arabia; ^2^ Pharmaceutical Organic Chemistry Department, Faculty of Pharmacy, Assiut University, Assiut, Egypt; ^3^ Applied Medical Science College, Medical Laboratory Technology Department, Northern Border University, Arar, Saudi Arabia; ^4^ Medicinal Chemistry Department, Faculty of Pharmacy, Deraya University, Minia, Egypt; ^5^ Pharmacology Department, College of Pharmacy, Jouf University, Sakaka, Saudi Arabia; ^6^ Institute of Biological and Chemical Systems, IBCS-FMS, Karlsruhe Institute of Technology, Karlsruhe, Germany; ^7^ Medicinal Chemistry Department, Faculty of Pharmacy, Minia University, Minia, Egypt; ^8^ Apogee Pharmaceuticals, Burnaby, BC, Canada

**Keywords:** salicylic acid, Cushman, Schiff bases, antibacterial, DNA, gyrase, topoisomerase

## Abstract

DNA gyrase and topoisomerase IV show great potential as targets for antibacterial medicines. In recent decades, various categories of small molecule inhibitors have been identified; however, none have been effective in the market. For the first time, we developed a series of disalicylic acid methylene/Schiff bases hybrids (**5a-k**) to act as antibacterial agents targeting DNA gyrase and topoisomerase IV. The findings indicated that the new targets **5f-k** exhibited significant antibacterial activity against Gram-positive and Gram-negative bacteria, with efficacy ranging from 75% to 115% of the standard ciprofloxacin levels. Compound **5h** demonstrated the greatest efficacy compared to the other compounds tested, with minimum inhibitory concentration (MIC) values of 0.030, 0.065, and 0.060 μg/mL against *S. aureus*, *E. coli*, and *P. aeruginosa*. **5h** had a MIC value of 0.050 μg/mL against *B. subtilis*, which is five times less potent than ciprofloxacin. The inhibitory efficacy of the most potent antibacterial derivatives **5f**, **5h**, **5i**, and **5k** against *E. coli* DNA gyrase was assessed. The tested compounds demonstrated inhibitory effects on *E. coli* DNA gyrase, with IC_50_ values ranging from 92 to 112 nM. These results indicate that **5f**, **5h**, **5i**, and **5k** are more effective than the reference novobiocin, which had an IC_50_ value of 170 nM. Compounds **5f**, **5h**, **5i**, and **5k** were subjected to additional assessment against *E. coli* topoisomerase IV. Compounds **5h** and **5i**, which have the highest efficacy in inhibiting *E. coli* gyrase, also demonstrated promising effects on topoisomerase IV. Compounds **5h** and **5i** exhibit IC_50_ values of 3.50 µM and 5.80 µM, respectively. These results are much lower and more potent than novobiocin’s IC_50_ value of 11 µM. Docking studies demonstrate the potential of compound **5h** as an effective dual inhibitor against *E. coli* DNA gyrase and topoisomerase IV, with ADMET analysis indicating promising pharmacokinetic profiles for antibacterial drug development.

## Highlights

• A series of new schiff base derivatives of disalicylic acid scaffold was developed.
• The structures of new compounds were validated by different spectral methods.
• The new compounds were evaluated as antibacterial agent targeting DNA gyrase and Topoisomerase IV.
• Molecular Docking and ADMET studies were investigated.

## 1 Introduction

The emergence of antibiotic resistance poses a significant risk to human health, and searching for novel antibacterial agents is a pressing concern (Chinemerem Nwobodo et al., 2022). The research community has faced significant challenges in identifying novel antibacterial drugs in recent decades (Terreni et al., 2021). To resolve this complex challenge, it is critical to successfully pursue the primary goal of identifying novel antibacterial medication classes (Miethke et al., 2021). As a result, significant research is being conducted to identify novel antibacterial drugs with greater efficacy in combating antibiotic resistance.

DNA gyrase is an important enzyme for bacteria’s survival because it plays a significant role in DNA replication, transcription, and recombination. It facilitates the breaking and rejoining of the DNA strand (Collin et al., 2011). Topoisomerase IV, similar to DNA gyrase, plays a crucial role in bacterial cell division. DNA gyrase and topoisomerase IV both put together tetrameric complexes with subunits that are needed to bind, cut, and transport DNA. The DNA gyrase consists of two GyrA and two GyrB subunits, whereas the topoisomerase IV is composed of two ParC and two ParE subunits (Collin et al., 2011; Tomašić and Peterlin Masic, 2014). Cleavage and rejoining of the DNA molecule needs the hydrolysis of an ATP molecule. The ATP-binding site is found on the GyrB subunit of DNA gyrase and the ParE component of topoisomerase IV. These enzymes offer great potential for new antibacterial agents to target due to their comparable structures, thereby reducing the risk of bacterial resistance developing (Nyerges et al., 2020; Al-Wahaibi et al., 2021a; Frejat F. O. A. et al., 2022; Abdel-Aziz et al., 2023; Elbastawesy et al., 2023).

Salicylic acid is a potent antibacterial agent commonly used in medicine due to its low toxicity. The phenolic structure of salicylic acid may be responsible for its antibacterial action (da Rocha Neto et al., 2015; Rad et al., 2021). The antibacterial properties of phenolic acids may be attributed to their capacity to modify microbial cell permeability, allowing macromolecule release from the interior, such as ribose and Na glutamate (He et al., 2011; Franklin and Snow, 2013). They can also affect membrane function, including electron transport, nutrition absorption, and nucleic acid synthesis. Furthermore, they can interact with membrane proteins, altering their structure and functions (Tiwari et al., 2009; Liu et al., 2016).

Schiff bases, compounds containing the azomethine functional group (C=N), have become significant in medicine and pharmaceuticals (Mahdy et al., 2022). They are highly versatile organic synthetic intermediates and exhibit various biological activities, including antibacterial and antifungal properties (Imran et al., 2014; Dzeikala and Sykula, 2018). Schiff bases serve as valuable intermediates in synthesizing numerous heterocyclic ring systems (Shanty et al., 2017). Schiff bases are substrates in synthesizing several commercial and physiologically useful compounds through ring closure, cycloaddition, and replacement reactions (Chavan et al., 2011). In addition, Schiff bases generated from different heterocycles have been documented to have diverse biological properties (Somwanshi, 2020).

**FIGURE 1:**
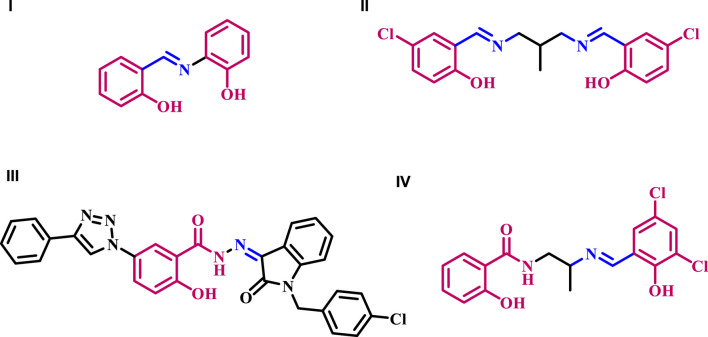
Structures of antibacterial compounds **I-IV**.

Schiff bases derived from salicylic acid or salicylaldehyde have been identified as potential antibacterial agents. As an illustration, *N*-(salicylidene)-2-hydroxyaniline **I** (Figure 1) is highly efficient against *Mycobacterium tuberculosis* H37Rv. It demonstrates an MIC value of 8 μg/mL (Souza et al., 2007). Compound **4**’s selectivity was tested with J774 macrophages. Despite testing at 1,000 μg/mL concentration, compound **4** did not show cytotoxic effects on J774 macrophages. Over 80% of macrophage cells are viable under these conditions, proving compound **4**’s strong selectivity.

Shi et al. (Shi et al., 2007) reported the synthesis and antibacterial activity of Schiff bases formed by condensing 5-chloro-salicylaldehyde with primary amines. All produced compounds were tested for antibacterial activity against two g (+) bacterial strains (*B*. *subtilis* and *S*. *aureus*) and two g (−) strains (*E*. *coli* and *P*. *fluorescence*). Compound **II** (Figure 1) exhibited the most potent activity against *B. subtilis* (MIC = 1.8 μg/mL), *P. fluorescence* (MIC = 3.6 μg/mL), and *E. Coli* (MIC = 4.9 μg/mL), in comparison to the reference Kanamycin with MIC values of 0.39, 3.9, and 3.9 μg/mL, respectively.

In another study from our laboratory (Abdu-Allah et al., 2017), we detailed the development of a new series of 1*H*-1,2,3-triazolylsalicyl hydrazones tested as potential antitubercular agents against *M. tuberculosis* H37Rv. The results showed compound **III** (Figure 1) had a higher potency, with an MIC value of 0.39 μg/mL, compared to the reference ethambutol with an MIC of 3.13 μg/mL. Compound **III** and isoniazid, rifampicin, and moxifloxacin were tested for potency against dominant forms of *mycobacterium* using a nutrition starvation paradigm at a dose of 10 μg/mL. The results demonstrated that compound **III** reduced bacterial count by 2.4 logs, comparable to the action of the reference medications.

A series of Schiff bases derived from salicylic acid hydrazide was designed, synthesized, and investigated for their biological activity as inhibitors of *E. coli* b-Ketoacyl-acyl carrier protein synthase III (ecKAS III) (Cheng et al., 2010). The results indicated that compound **IV** (Figure 1) exhibits potent inhibitory activity against ecKAS III and has a high affinity for binding to Gly152/Gly209 of ecKAS III. It also fits well into the substrate tunnel and is potentially an antibiotic agent. The MIC values of **IV** against various bacteria range from 0.20 to 3.13 μg/mL.

### 1.1 Rational design

The compound **NSC 14778**, also known as disalicylic acid methylene, was specifically designed to inhibit DNA methyltransferases (DNMTs), enzymes involved in DNA methylation. DNA methylation leads to hypermethylation and subsequent suppression of genes, including tumor suppressor genes. **NSC 14778** has been shown to exhibit inhibitory activity on DNMTs within the micromolar range (Leroy et al., 2019). To the best of our knowledge, there is no available data regarding the antimicrobial properties of **NSC 14778** or any of its derivatives.

Schiff bases contain an imine or azomethine group (-C=N-) formed via the condensation of primary amine functionalities with carbonyl compounds. This group’s pharmacological potential stems from their capacity to influence the activity of several enzymes engaged in metabolic reactions by forming complex compounds with bivalent and trivalent metals situated in the active centers of these enzymes (Ceramella et al., 2022).

Moreover, it was reported that in the case of Schiff bases with a benzene ring directly attached to an azomethine moiety, the presence of a hydroxyl group in the two-position (ortho) to the azomethine moiety may aid in the development of intramolecular resonance-stabilized hydrogen bonds. These hydroxyl groups increase the molecule’s overall thermodynamic stability and, thus, its activity (Raczuk et al., 2022). Additionally, the phenolic hydroxyl group is recognized to have significant antibacterial activity against numerous therapeutically relevant pathogens (Manso et al., 2021; Ecevit et al., 2022).

**FIGURE 2:**
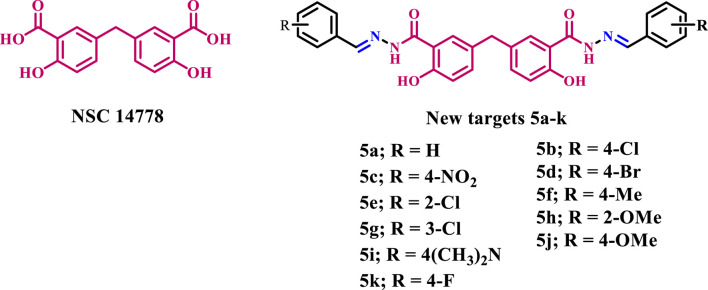
Structures of **NSC 14778** and new targets **5a-k**.

Based on the results above and our ongoing research on developing medicinally active antimicrobials (Youssif et al., 2016; Haseeb et al., 2017; Alkhaldi et al., 2019; Ibrahim et al., 2020; Shaykoon et al., 2020; Al-Wahaibi et al., 2021a; Al-Wahaibi et al., 2021b; Hofny et al., 2021; Frejat F. O. A. et al., 2022; Frejat F. O. et al., 2022; Abdel-Aziz et al., 2023; Elbastawesy et al., 2023; Gomaa et al., 2023; Aly et al., 2024), we present herein the design and synthesis of novel Schiff bases **5a-k** (Figure 2) derived from methylene disalicylic acid hydrazide. The new compounds **5a-k** were evaluated for their antibacterial activity against *S. aureus* and *B. subtilis*, which are representative of Gram-positive pathogens, and *E. coli* and *P. aeruginosa*, which are Gram-negative strains. Ciprofloxacin was used as a reference drug. In addition, the synthesized compounds **5a-k** were evaluated for their antifungal activity against *A. flavus* and *C. albicans*, with Fluconazole as a reference drug. The minimum inhibitory concentrations (MICs) of the most active compounds against the tested microorganisms were calculated against Ciprofloxacin and/or Fluconazole. Moreover, the inhibitory potency of the most active derivatives against *E. coli* DNA gyrase and Topoisomerase IV as probable targets was evaluated.

## 2 Results and discussion

### 2.1 Chemistry

**SCHEME 1:**
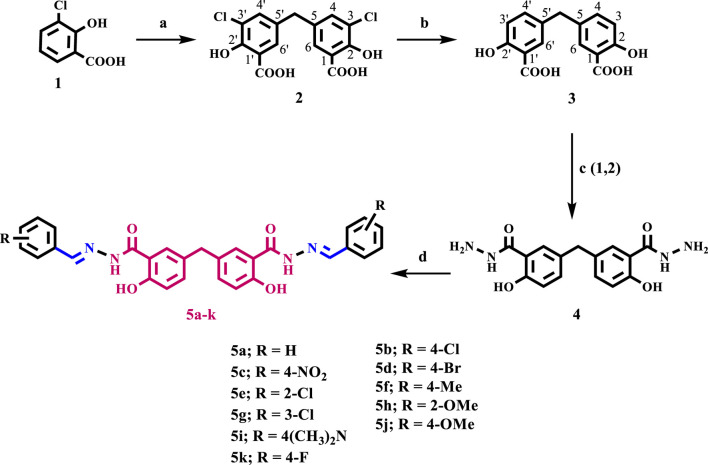
Synthesis of target compounds **5a-k.**

Synthesis of the target compounds **5a-k** is outlined in Scheme 1. First, the procedure developed for preparing the key intermediate, compound **3,** from 3-chlorosalicyclic acid **one** involved two steps (Cushman and Kanamathareddy, 1990), as outlined in Scheme 1. The current study’s procedures differ slightly from those used in Cushman and Suseela’s research (Cushman and Kanamathareddy, 1990). The synthesis started with the preparation of the methylene bridged 3-chlorosalicylic acid dimer **2 (**DSA-Cl_2_), in 98% yield, via condensation of two molecules of 3-chlorosalicylic acid **1** with paraformaldehyde in concentrated sulfuric acid (H_2_SO_4_). It is important to note that the chlorine atom was utilized as a protective group to regulate the regio-chemistry in the dimerization reaction and to inhibit the further interaction of DSA-Cl_2_ with formaldehyde in the presence of acid, which would result in the formation of phenol-formaldehyde polymers. The ^1^H NMR spectrum of compound **2** is characterized by a singlet signal at δ 3.87 ppm corresponding to the benzylic CH_2_ group. Two doublet signals each of two protons at δ 7.67 and 7.45 ppm of 6, 6ʼ and 4, 4^ʼ^ aromatic protons, respectively. The ^13^C NMR spectrum of **two** revealed a characteristic signal at δ 38.5 of benzylic carbon and a signal at 171.7 ppm of carboxylic group carbons. Moreover, the high-resolution mass spectrometry of compound **2** shows [M-H]^-^ peak at m/*z =* 354.9783, which validates its structure (See Supplementary Material).

Reagents and reaction conditions: a) HCHO, H_2_SO_4_, 35°C, 18 h, 98%; b) KHCOO, Pd/C, KOH, 2-PrOH/H_2_O, 70°C, 4 h, 92%; c) 1-EtOH/Conc. H_2_SO_4_, 2- Hydrazine hydrate, reflux 6 h; d) Aldehyde, EtOH, reflux 6 h.

**FIGURE 3:**
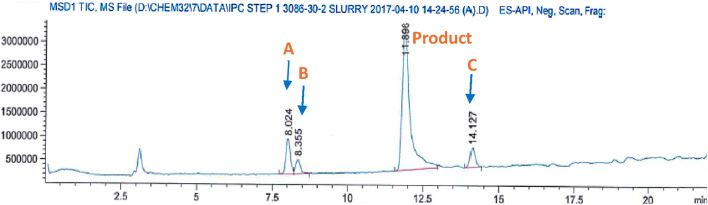
Mass analysis of crude DSA-Cl_2_ (impurities profiling).

It is important to note that compound **1** experiences side reactions that produce impurities when there is an excess of formaldehyde. The purity of the produced DAS-Cl_2_ was determined using liquid chromatography-mass spectrometry (LC-MS), while the standard chromatography method proved challenging for purification. The LC-MS analysis for impurity profiling detected three side products with retention times (RT in minutes) of 8.024, 8.355, and 14.127 (Figures 3A–C).

**SCHEME 2:**
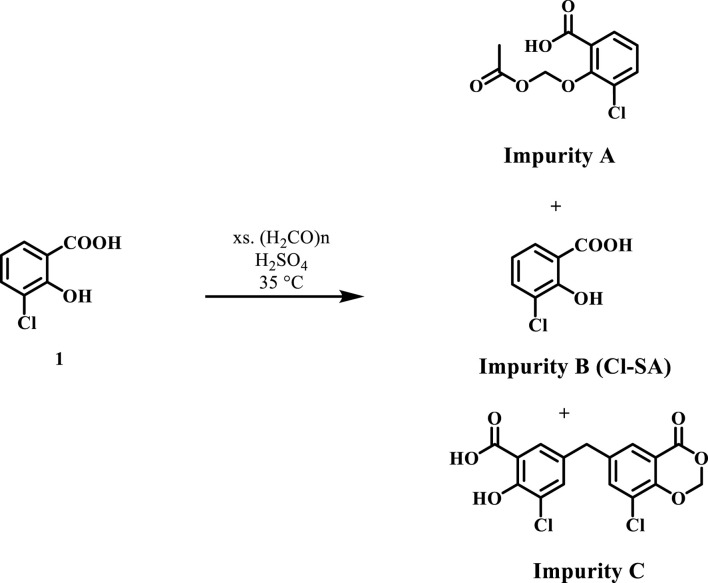
Impurities formed due to excess formaldehyde in the initial condensation step.

**FIGURE 4:**
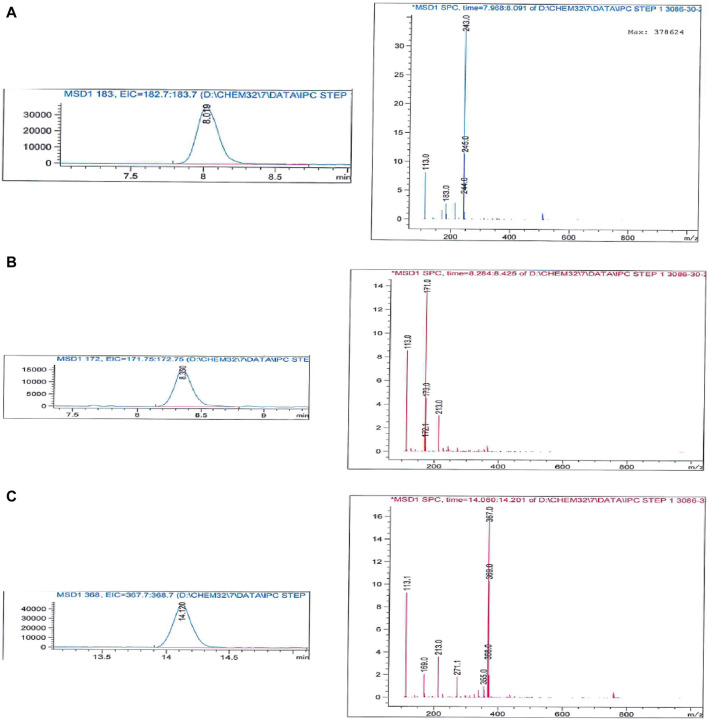
Mass spectrum of impurities formed due to excess formaldehyde in the initial condensation step **(A)** impurity **(A, B)** impurity **(B, C)** impurity **(C)**.

Impurity **A** at *m/z*: 244 corresponds to the formylated phenolic OH derivative, impurity **B** at *m/z*: 171 corresponds to the starting material chlorosalicylic acid (Cl-SA), and **C** acetal impurity at *m/z*: 369 (Scheme 2; Figure 4A–C). These side products were also shown to be exacerbated with longer reaction times or higher paraformaldehyde concentrations. The preliminary structures of these impurities (Scheme 2) were proposed based on mass data and the feasibility of various chemical interactions involving paraformaldehyde and chlorosalicylic acid.

The next step involved the reductive dehalogenation of **two** using potassium formate as a hydride source and Pd/C as a catalyst to afford disalicylic acid dimer **3** in 92% yield. Hydrodehalogenation of the chlorine atom in compound **2** to provide the disalicylic acid dimer **3** was initially conducted using molecular hydrogen (hydrogen gas) (Cushman and Kanamathareddy, 1990). We observed a slow reaction, and the conversion was incomplete after 24 h. Therefore, catalytic transfer hydrogenation (CTH) with organic hydrogen donors like potassium formate (HCOOK) as a reductant was used to avoid the need for high-pressure hydrogen gas. CTH exhibited a much higher reaction rate with potassium formate than molecular hydrogen. Moreover, potassium formate is easy to handle, less toxic, and less flammable; thus, it is more favorable for upscale synthesis.

The ^1^H NMR spectrum of compound **3** is characterized by a singlet signal at δ 3.85 ppm corresponding to the benzylic CH_2_ group. Two doublet signals of two protons at δ 7.62 and 6.89 ppm of 6, 6ʼ (J = 2.2 Hz) and 3, 3^ʼ^ (J = 8.5 Hz) aromatic protons, respectively. The spectrum is also characterized by a dd (J = 8.5, 2.2 Hz) signal of two protons corresponding to 4, 4^ʼ^ aromatic protons. The ^13^C NMR spectrum of **3** revealed a characteristic signal at δ 39.2 ppm of benzylic carbon and a signal at 172.0 ppm of carboxylic group carbons. Moreover, the high-resolution mass spectrometry of compound **2** shows [M-H]^-^ peak at m/*z =* 289.0616, which validates its structure (See Supplementary Material).

The residual formaldehyde in compound **2** resulted in potent poisoning of the Pd/C catalyst. The mechanism of catalyst poisoning is believed to be the generation of carbon monoxide on the palladium surface via β-hydride elimination of formaldehyde under alkaline conditions. Similarly, the conversion of methanol to formaldehyde via β-hydride elimination (Smythe et al., 2009), or the cleavage of acetal (impurity **C**, Scheme 2) under alkaline conditions to release formaldehyde, were also found to be highly detrimental to the success of the hydrodehalogenation procedure.

In order to remove paraformaldehyde, which results in catalyst poisoning during the reduction step (hydrodehalogenation), and to further convert the acetal impurity **C** (Scheme 2) into the DSA-Cl_2_, the crude DSA-Cl_2_ starting material was treated with 1 M KOH solution at 90°C to conduct the remediation under Cannizzaro conditions, which can induce disproportionation of formaldehyde into formate and methoxide and also simultaneously convert the acetal impurities into the product.

The carboxylic acid **3** was subjected to Fisher esterification with absolute ethanol in the presence of H_2_SO_4_ as a dehydrating agent for 20 h. This was followed by refluxing with hydrazine monohydrate for 6 h, forming compound **4** with an overall yield of 74% for the two steps. The IR spectrum of compound **4** revealed a broad peak in the range of 3,400 to 2,500 cm^-1^ (Phenolic OH), two distinct peaks at 3,333 and 3,195 cm^-1^ for the NH_2_ group, a sharp and intense peak at 1,633 cm^-1^ for the amidic C=O group, and peaks at 824 and 698 cm^-1^ indicating bending of the Ar-CH group. The ^1^H NMR spectra of compound **4** revealed four distinct singlet signals: a signal at δ 12.08 ppm for two phenolic OH protons, a signal at δ 9.95 ppm for the amidic protons, a signal at δ 4.62 ppm for two NH_2_ groups, and a signal at δ 3.74 ppm for methylene protons (Ar-CH_2_). Also, the spectrum revealed characteristic signals of the six aromatic protons.

Schiff base derivatives, **5a-k**, were prepared by refluxing compound **4** with appropriate (un)substituted aldehyde in ethanol for 4–6 h, affording **5a-k** in good yields. The validity of the structures of the targets **5a-k** was confirmed through the use of ^1^H NMR, MS spectroscopy, and elemental microanalysis. The ^1^H NMR spectrum **5j** (R = 4-OMe) displayed five distinct singlet signals. These included a singlet at δ 11.64 ppm, representing two protons of two amidic-NH groups; a singlet at δ 8.41 ppm, representing two phenolic protons; a singlet at δ 7.80 ppm, representing azomethine protons; a singlet at δ 3.89 ppm, representing benzylic protons, and a singlet signal at δ 3.82 ppm, representing six protons corresponding to two methoxy groups. The spectrum also displayed the distinctive signals of 14 aromatic protons.

### 2.2 Biology

#### 2.2.1 Antimicrobial sensitivity test

The antimicrobial activity of compounds **5a-k** was assessed using a modified Kirby-Bauer disc diffusion method (Alkhaldi et al., 2019; Al-Wahaibi et al., 2021b; Manso et al., 2021; Ecevit et al., 2022). Table 1 displays the findings, where compounds **5a-k** were evaluated for their antibacterial activity against *S. aureus* and *B. subtilis*, which are representative of Gram-positive pathogens, and *E. coli* and *P. aeruginosa*, which are Gram-negative strains. Ciprofloxacin was used as a reference medication. The findings revealed that most tested compounds exhibited potential antibacterial action against the test organisms (Table 1). Compounds **5f-k** had promising antibacterial action against Gram-positive and Gram-negative bacteria, ranging from 75% to 115% of typical ciprofloxacin levels.

**TABLE 1:** Inhibitory zone diameter (mm) of compounds **5a-k**.

Sample		Inhibition zone diameter (mm/mg Sample)					
		Bacterial species				Fungi	
		(G^+^)		(G^−^)			
		*Bacillus subtilis*	*Staph. aureus*	*Escherichia Coli*	*Pseudomonas aeruginosa*	*Aspergillus flavus*	*Candida albicans*
Control: DMSO		0.0	0.0	0.0	0.0	0.0	0.0
Standard	Ciprofloxacin	40	40	40	40	--	--
	Fluconazole	--	--	--	--	40	40
**5a**		21	25	24	21	0.0	0.0
**5b**		26	27	26	26	0.0	7
**5c**		26	27	27	25	0.0	11
**5d**		28	31	30	26	0.0	10
**5e**		27	30	28	28	0.0	12
**5f**		37	37	33	33	0.0	15
**5g**		33	35	30	30	0.0	0.0
**5h**		43	46	39	38	0.0	0.0
**5i**		41	42	38	37	0.0	0.0
**5j**		31	33	30	30	0.0	0.0
**5k**		39	41	37	35	0.0	0.0

Compounds **5h** (X = 2-OMe) and **5i** (X = 4-dimethylamino) exhibited the highest activity level among the derivatives tested. They showed higher activity against *S. aureus* and *B. subtilis* compared to ciprofloxacin. Additionally, they displayed similar activity to ciprofloxacin against *E. coli* and *P. aeruginosa*, which are Gram-negative pathogens. Compound **5f** (with X = 4-Me) and compound **5k** (with X = 4-F) exhibited antibacterial action as strong as ciprofloxacin against both *S. aureus* and *E. coli*. Compound **7**, the 3-chloro derivative (X = 3-Cl), exhibited strong antibacterial activity against *S. aureus*, a Gram-positive strain. Its activity was 88% compared to ciprofloxacin. However, it exhibited only 75% activity of ciprofloxacin against Gram-negative strains, as shown in Table 1. Compounds **5a-e** exhibited a moderate level of antibacterial activity, ranging from 53% to 77% that of ciprofloxacin activity, against both Gram-positive and Gram-negative pathogens.

In addition, the synthesized compounds **5a-k** were evaluated for their antifungal activity against *A. flavus* and *C. albicans*, with Fluconazole as a reference medication (Table 1). The results indicated that the compounds examined did not exhibit antifungal effects against *A*. *flavus*. However, they showed mild activity against *C. albicans,* ranging from 18% to 38% of fluconazole, with compound **5f** being the most active (38% of fluconazole).

#### 2.2.2 Minimum inhibitory concentration test

The antibacterial activity of the most potent components **5f**, **5h**, **5i**, and **5k** (Aly et al., 2024) was tested using a twofold serial dilution approach on a 96-well microtiter plate. Table 2 displayed these compounds’ minimum inhibitory concentrations (MICs) against the tested microorganisms, using the reference medication ciprofloxacin.

**TABLE 2:** Antimicrobial activities of compounds **5f**, **5h**, **5i**, and **5k**.

Minimum inhibitory concentration (MIC) in µg/ml				
Compound	Bacterial species			
	(G^+^)		(G^−^)	
	*Bacillus subtilis*	*Staphylococcus aureus*	*Escherichia coli*	*Pseudomonas aeruginosa*
**5f**	0.125	0.070	0.125	0.125
**5h**	0.050	0.030	0.065	0.060
**5i**	0.070	0.035	0.065	0.065
**5k**	0.125	0.060	0.125	0.125
Ciprofloxacin	0.010	0.030	0.060	0.060

The findings of this *in vitro* assay test align with the results of the antimicrobial sensitivity test. Compound **5h** (X = 2-OMe) exhibited the highest potency among the compounds tested, with MIC values of 0.030, 0.065, and 0.060 μg/mL against *S. aureus*, *E. coli*, and *P. aeruginosa*. It demonstrated equivalent potency to ciprofloxacin against the tested organisms, while it had an MIC value of 0.050 μg/mL against *B. subtilis*, which is five times less potent than ciprofloxacin. Compound **5i (**X = 4-dimethylamino) had the second highest activity. Its MIC values were similar to those of compound **5h** and ciprofloxacin against *S. aureus*, *E. coli*, and *P. aeruginosa*. However, it was 7 times less potent than ciprofloxacin against *B. subtilis*. Compounds **5f** and **5k** showed promising action against tested species, particularly against *S. aureus*, with MIC values of 0.070 and 0.060 μg/mL, respectively, compared to ciprofloxacin (MIC = 0.030 μg/mL). Interestingly, all tested compounds had a mildly negative effect on the Gram-positive bacterium *B. subtilis*.

#### 2.2.3 *E. coli* DNA gyrase and topoisomerase IV inhibitory assay

The inhibitory potency of derivatives **5f**, **5h**, **5i**, and **5k** against *E. coli* DNA gyrase was evaluated using the *E. coli* DNA gyrase assay (Frejat F. O. et al., 2022). The results are reported as residual activities (RAs) of the enzyme at a concentration of 1 µM for the compounds or as IC_50_ values for compounds with an RA less than 50% **(** Table 3 **)**.

**TABLE 3:** The inhibitory actions of compounds **5f**, **5h**, **5i**, 5k, and Novobiocin on *E. coli* DNA gyrase and topoisomerase IV.

Compound	IC_50_ (nM)^a^ or RA (%)^b^	IC_50_ (µM)^a^ or RA (%)^b^
	*E. Coli* DNA gyrase	*E. Coli* Topo IV
**5f**	53%	77%
**5h**	92 ± 5 nM	3.50 ± 0.30 µM
**5i**	97 ± 6 nM	5.80 ± 0.40 µM
**5k**	112 ± 7 nM	55%
Novobiocin	170 ± 20 nM	11 ± 2 µM

^a^ Concentration of compound that inhibits the enzyme activity by 50%.

^b^ Residual activity of the enzyme at 1 µM of the compound.

This assay’s results supplement the antimicrobial activity study’s findings. Compounds **5f**, **5h**, **5i**, and **5k** inhibited *E. coli* DNA gyrase with IC_50_ values ranging from 92 to 112 nM compared to the reference novobiocin (IC_50_ = 170 nM). Compounds **5h**, **5i**, and **5k** showed higher inhibitory activity against *E. coli* DNA gyrase (IC_50_ = 92 ± 5, 97 ± 6, and 112 ± 7, respectively) than the positive control novobiocin. Finally, compound **5f** was the least effective compound as an *E. coli* DNA gyrase inhibitor, with an RA of 53%.

Compounds **5f**, **5h**, **5i**, and **5k** underwent further evaluation against *E. coli* topoisomerase IV (Frejat F. O. et al., 2022), as shown in Table 3. Compounds **5h** and **5i**, the most effective inhibitors of *E. coli* gyrase, also showed encouraging effects on topoisomerase IV. The IC_50_ values for compounds **5h** and **5i** are 3.50 µM and 5.80 µM, respectively. These values are significantly lower, indicating that they are more potent than the IC_50_ value of 11 µM for novobiocin. Based on these data, it can be concluded that both compounds **5h** and **5i** show promise as dual-target inhibitors against DNA gyrase and topoisomerase IV, especially following optimization.

#### 2.2.4 Cell viability assay

**FIGURE 5:**
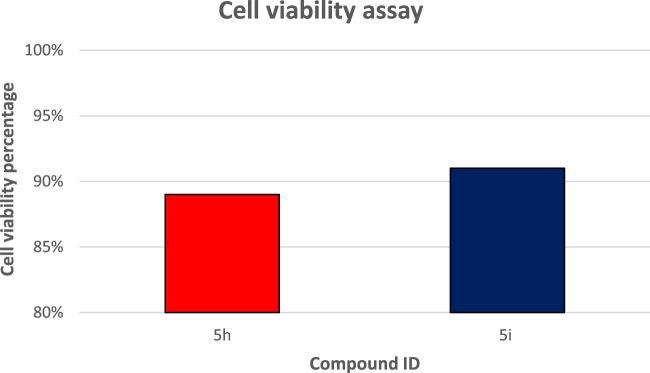
Cell viability assay results on the normal cell line of compound **5h** and **5i**.

This test investigates the influence of **5h** and **5i**, the most potent derivatives in all *in vitro* studies, on normal cell lines to establish their safety. The compounds’ vitality impact was evaluated using the MCF-10A cell line, a normal human mammary gland epithelial cell line. After a 4-day incubation period on MCF-10A cells with a concentration of 50 µM for each compound tested, cell viability was measured using the MTT assay (Mekheimer et al., 2022; Mahmoud et al., 2024). The results revealed that neither **5h** nor **5i** were cytotoxic, with cell viability exceeding 89% and 91% at 50 μM, respectively, Figure 5.

### 2.3 Docking study into *E. Coli* DNA gyrase B and *E. Coli* topoisomerase IV

This study conducted a detailed computational docking analysis to elucidate the binding interactions between compounds **5f**, **5h**, and the reference drug Novobiocin with *E. coli* DNA gyrase B and *E. Coli* topoisomerase IV. The methodology involved Discovery Studio software, facilitating an in-depth exploration of the interaction mechanisms between the compounds and the target proteins.

To ensure the accuracy and relevance of our study, we integrated the crystallographic structure of the *E. coli* DNA gyrase B ligand complex (PDB ID: 4DUH) obtained from the Protein Data Bank. We specifically selected a structure where the loop with low crystallographic density is intact and well-defined, ensuring the most accurate representation of the protein conformation. This choice was based on the critical role this loop plays in the functionality and its potential impact on ligand binding. Recognizing the importance of structural integrity, additional details on the selection of this intact loop structure have been elaborated in the Methods section. The OPLS-AA (Optimized Potentials for Liquid Simulations - All Atom) force field was employed during the energy minimization process for the molecular systems examined. Using this force field was pivotal in achieving conformational stability for the molecular structures, thereby enhancing the precision and dependability of our computational investigations. Before commencing the docking procedure, the protein structure was comprehensively prepared to guarantee its accuracy. This preparation included protonation, a step that significantly contributed to the robustness and reliability of the docking analysis that followed.

**FIGURE 6:**
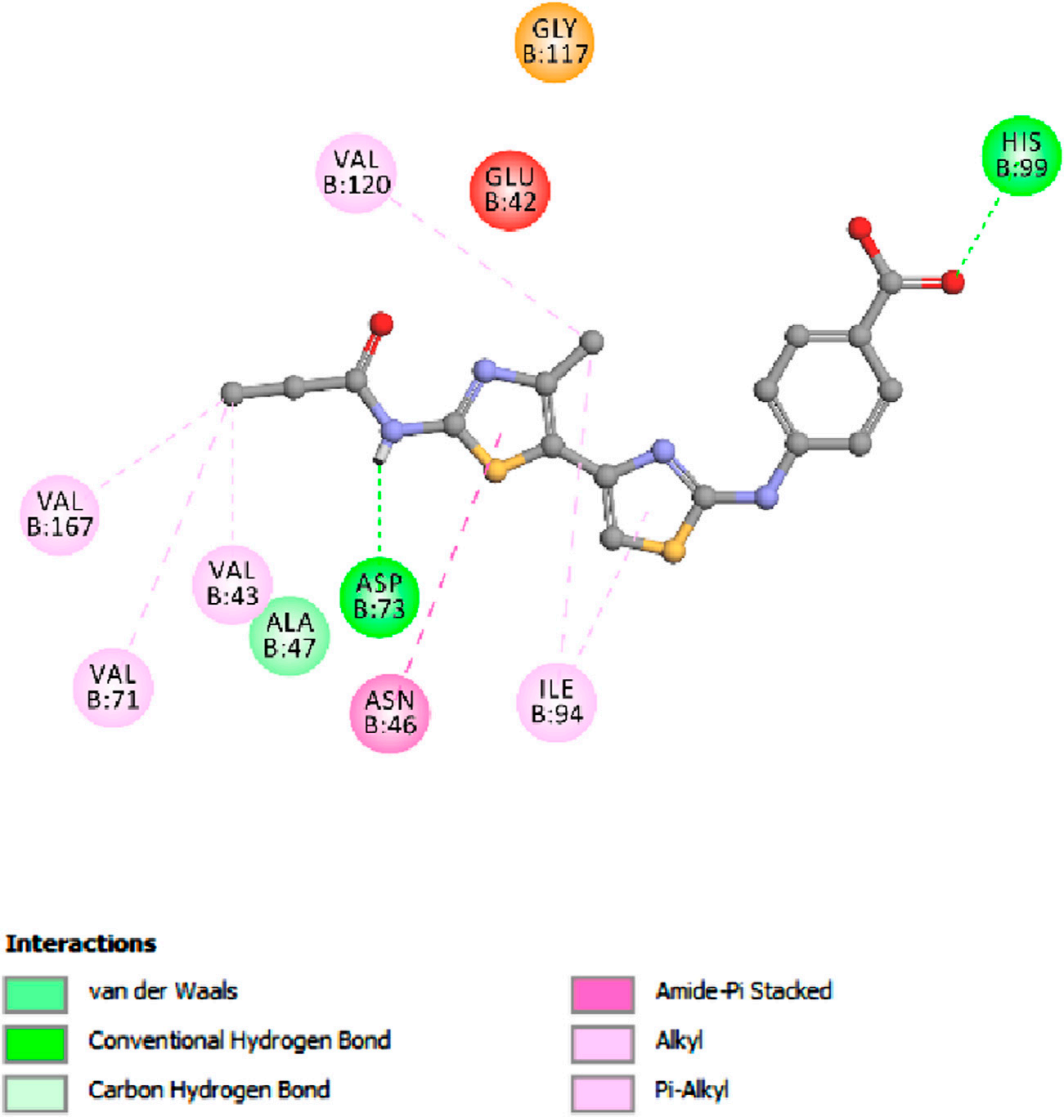
Docking representation model of co-crystallized ligand within the binding site of *E. coli* DNA gyrase B protein.

To ascertain the accuracy of the docking methodology, the co-crystallized ligand was re-docked into the active site of the *E. coli* DNA gyrase B protein. The re-docking process resulted in an S score of −7.15 kcal/mol, affirming the precision of the docking procedure as illustrated in Figure 6. The S score serves as an index to evaluate the binding affinity of a compound to its target protein within docking studies, where lower S scores, expressed in kilocalories per mole (kcal/mol), signify stronger binding affinities. Identifying pivotal hydrogen bond interactions between the ligand and specific amino acid residues within the *E. coli* DNA gyrase B protein underscored this validation step.

Notably, an essential hydrogen bond was observed between the amide nitrogen of the ligand and Asp 73, accompanied by additional hydrogen bonds with residues Gly 101 and Arg 136. These interactions are critical for stabilizing the ligand in the active site, underscoring the significance of such molecular interactions in facilitating the binding process. Further analysis of the docking scores concerning the *in vitro* activity levels of *E. coli* DNA gyrase B among the studied compounds revealed a direct correlation. The compound **5h**, which exhibited the highest *in vitro* activity against *E. coli* DNA gyrase B, recorded a docking score of −7.56 kcal/mol. In contrast, compound **5f**, with a lower *in vitro* activity, achieved a docking score of −6.95 kcal/mol. This pattern indicates a direct relationship between docking scores and biological activity, suggesting that higher docking scores correlate with increased affinity and, potentially, greater biological effectiveness. Moreover, Novobiocin, used as a reference drug in the study, displayed a docking score of −7.31 kcal/mol, positioning it within the range of affinities observed for compounds **5h** and **5f**.

This comparative analysis further validates the docking scores as a reliable metric for predicting the biological activity of compounds against the *E. coli* DNA gyrase B protein. In analyzing interactions between the tested compounds and the *E. coli* DNA gyrase B protein, compound **5h** has exhibited notable binding characteristics. A significant interaction was observed where the phenolic oxygen of compound **5h** acted as a hydrogen bond acceptor. This interaction involved the formation of a hydrogen bond with the critical amino acid residue Arg 136 in DNA gyrase, marking a crucial contribution to the compound’s binding affinity and specificity towards the enzyme. Further detailing the binding dynamics of compound **5h**, the study highlighted the role of its 2-methoxy phenyl group. This group engaged in a hydrogen bond with the amino acid residue Asn 46, adding another interaction layer critical for stabilizing the active site.

**FIGURE 7:**
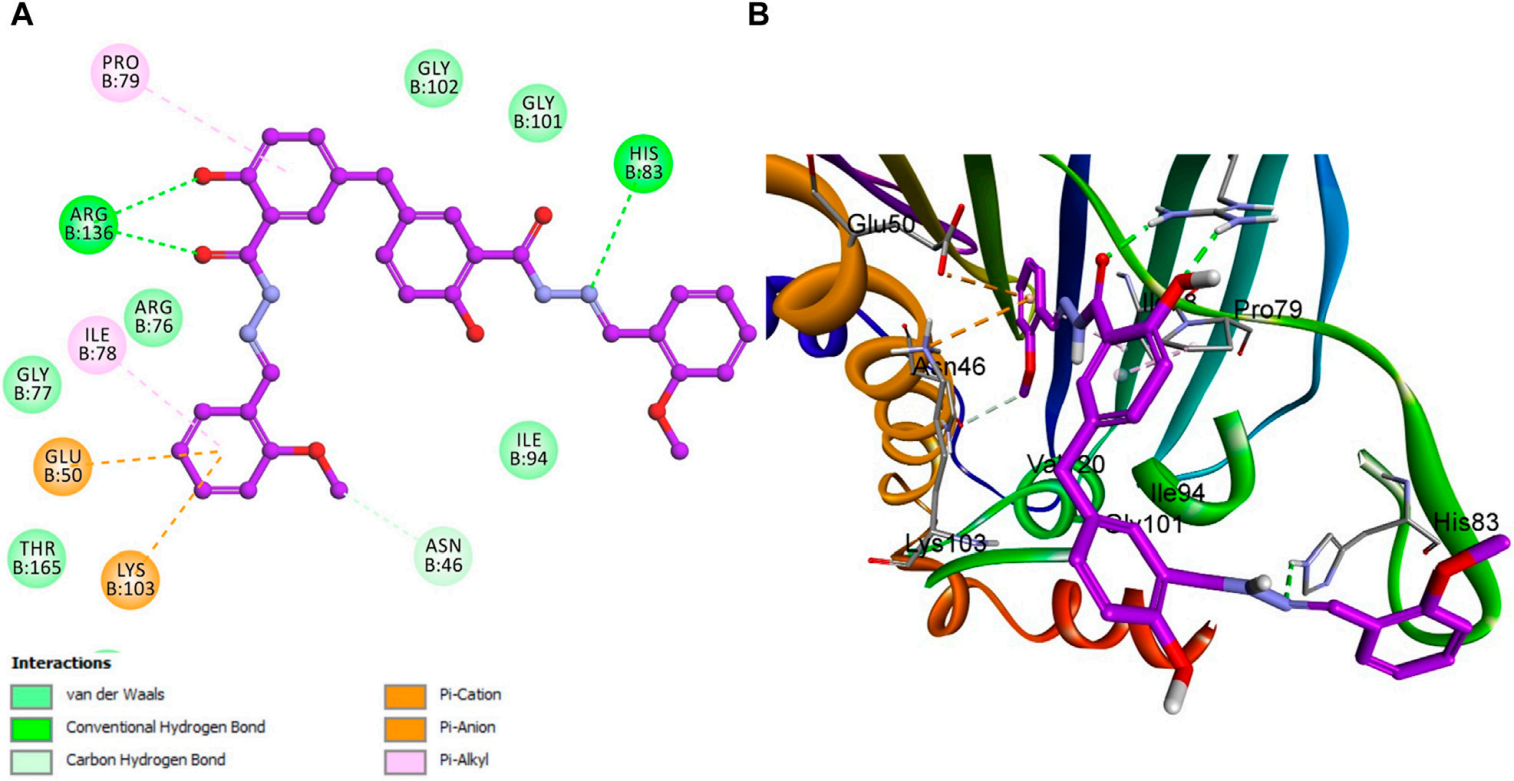
Docking representation models of compound **5h** within the binding site of *E. coli* DNA gyrase B protein; **(A)** 2D-docked model of compound **5h; (B)** 3D-docked model of compound **5h**.

Additionally, the aromatic rings of compound **5h** were involved in pi-stacking interactions with residues Ile 78 and Pro 78. These complex interactions, encompassing hydrogen bonding and pi-stacking, play a pivotal role in effectively binding compound **5h** to the *E. coli* DNA gyrase B protein, as illustrated in Figure 7. This comprehensive interaction profile underscores the multifaceted nature of **5h** with the target protein, contributing to its efficacy.

**FIGURE 8:**
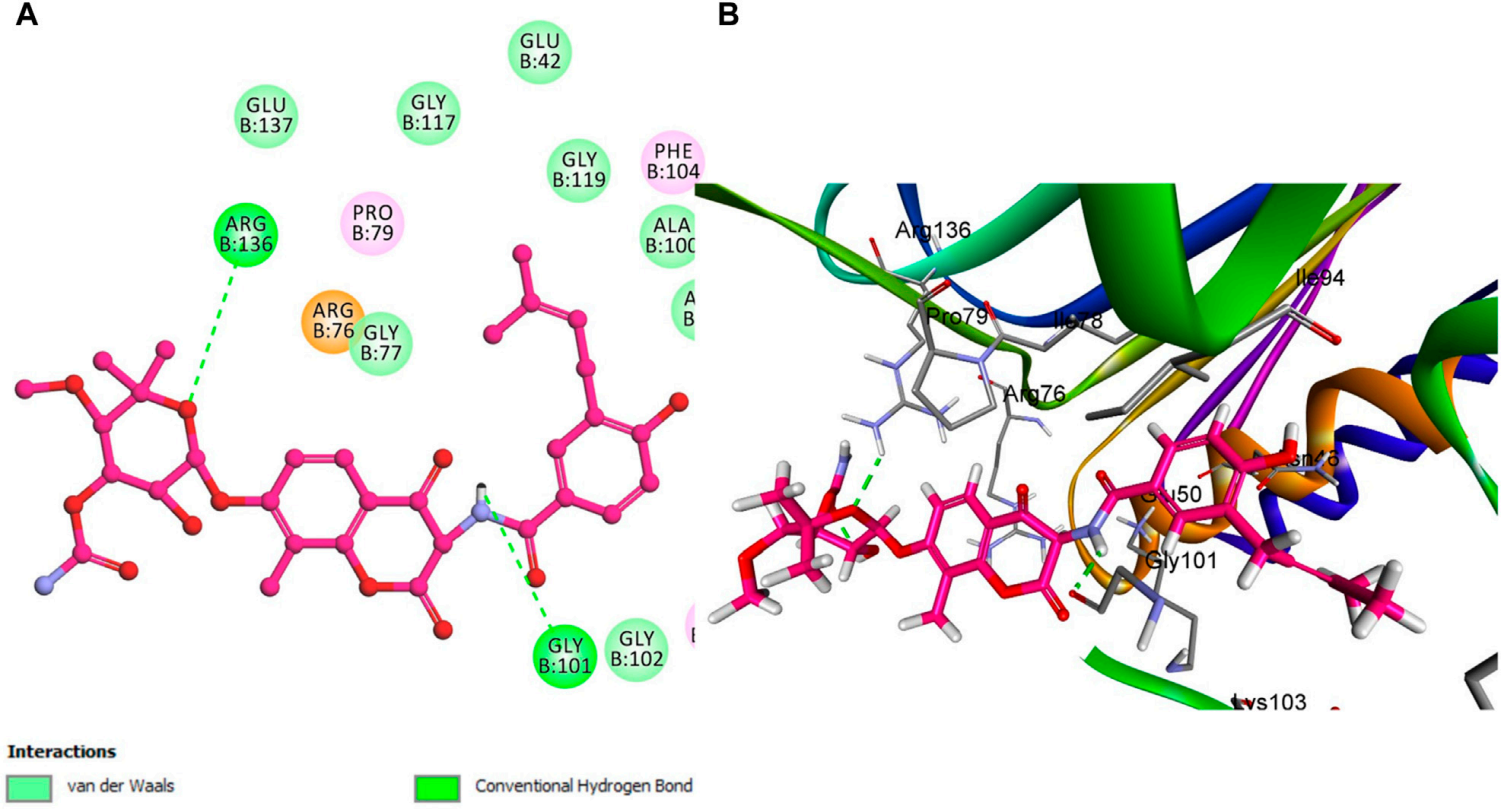
Docking representation models of Novobiocin within the binding site of *E. coli* DNA gyrase B protein; **(A)** 2D-docked model of Novobiocin**; (B)** 3D-docked model.

Similar to compound **5h**, reference Novobiocin exhibited notable interactions with the *E. coli* DNA gyrase B protein, with its amide nitrogen of the pyridine ring functioning as a hydrogen bond acceptor in conjunction with Gly 101, as depicted in Figure 8. However, this significant interaction results in Novobiocin having a comparatively lower binding affinity to the active site than compound **5h**. This reduced affinity can be attributed to the absence of hydrophobic pi-stacking interactions that are present in the binding profile of compound **5h**. The pi-stacking interactions contribute to a more stable and effective binding by enhancing the hydrophobic contact between the compound and the protein. The lack of these interactions in the case of Novobiocin suggests that, despite the hydrogen bonding, the overall binding efficacy is lessened due to the absence of crucial hydrophobic interactions that support stronger and more stable engagement with the active site.

**FIGURE 9:**
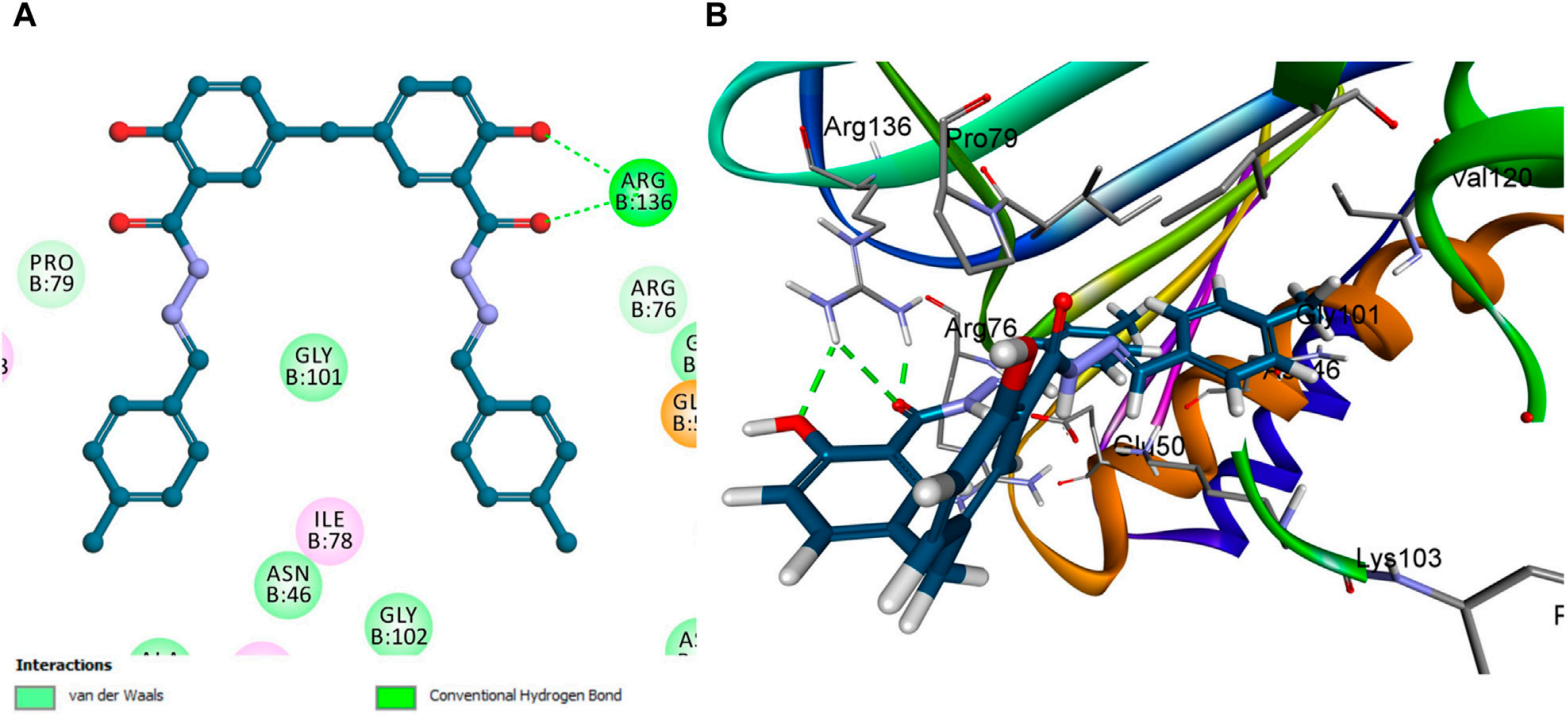
Docking representation models of compound **5f** within the binding site of *E. coli* DNA gyrase B protein; **(A)** 2D-docked model of compound **5f**; **(B)** 3D-docked model of compound **5f**.

However, compound **5f** is notably lacking in pi-stacking interactions (Figure 9) and has the lowest docking score among the evaluated compounds, including **5h** and Novobiocin. The experimental *in vitro* assays corroborate these computational findings, with compound **5f** showing a residual activity (RA) of 53%. In contrast, the IC_50_ values for compound **5h** and Novobiocin were 92 ± 5 nM and 170 ± 20 nM, respectively. These findings align the docking scores obtained through computational analysis with the biological activity observed *in vitro*. Specifically, the lower docking score of **5f**, coupled with its structural deficiency in pi-stacking interactions, accounts for its diminished binding affinity and reduced effectiveness compared to **5h** and Novobiocin.

In the context of our study on *E. coli* topoisomerase IV, the crystallographic structure of its ligand complex (PDB ID: 3FV5), as obtained from the Protein Data Bank, was integrated to provide a foundational framework for computational modeling. To evaluate the precision of our docking methodology, the co-crystallized ligand was re-docked into the active site of *E. coli* topoisomerase IV. This procedure yielded an S score of −6.88 kcal/mol, corroborating the methodological accuracy of our docking approach. Critical to this validation was identifying significant hydrogen bonding between the ligand and the amino acid residues Asp 69 and Arg 132, delineating crucial interactions underpinning ligand binding. A subsequent analysis that correlated docking scores with the observed *in vitro* activity levels for *E. coli* topoisomerase IV across the compounds under investigation revealed a direct relationship.

**FIGURE 10:**
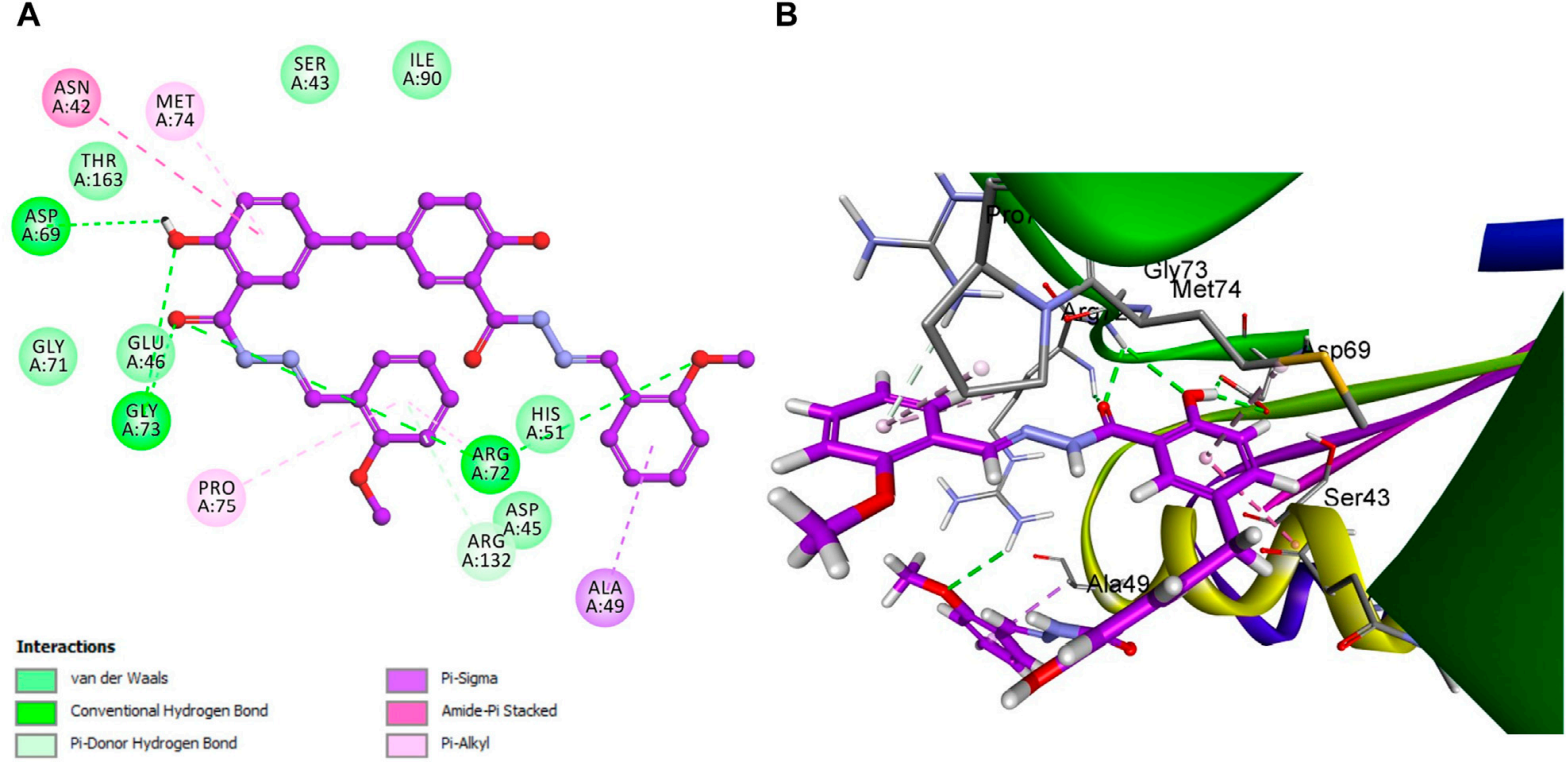
Docking representation models of compound **5h** within the binding site *E. coli* topoisomerase IV; **(A)** 2D-docked model of compound **5h**; **(B)** 3D-docked model of compound **5h**.

Specifically, compound **5h**, which exhibited the highest *in vitro* activity against *E. coli* topoisomerase IV, attained a docking score of −7.36 kcal/mol. In contrast, compound **5f**, associated with lower *in vitro* activity, presented a docking score of −6.45 kcal/mol. Furthermore, Novobiocin, serving as the reference compound within this study, recorded a docking score of −6.77 kcal/mol. This score positions Novobiocin within the affinity range identified for compounds **5h** and **5f**. In the comprehensive analysis focusing on the interaction profiles between the investigated compounds and *E. coli* topoisomerase IV, compound **5h** exhibited significant binding characteristics. Multiple interactions were delineated, notably with the phenolic hydroxyl group of **5h** acting as a hydrogen bond donor. This mechanism facilitated the establishment of a hydrogen bond with the crucial amino acid residue Asp 69 alongside a robust hydrogen bond formation with Gly 73, underscoring the affinity for the active site. Further examination of **5h** binding revealed the contributory role of its 2-methoxy phenyl group, which engaged in a hydrogen bond with the amino acid residue Arg 72. Complementing these interactions, the aromatic rings of **5h** participated in pi-stacking interactions with residues Ala 49 and Met 74, illustrating a complex interplay of binding interactions as evidenced in Figure 10.

**FIGURE 11:**
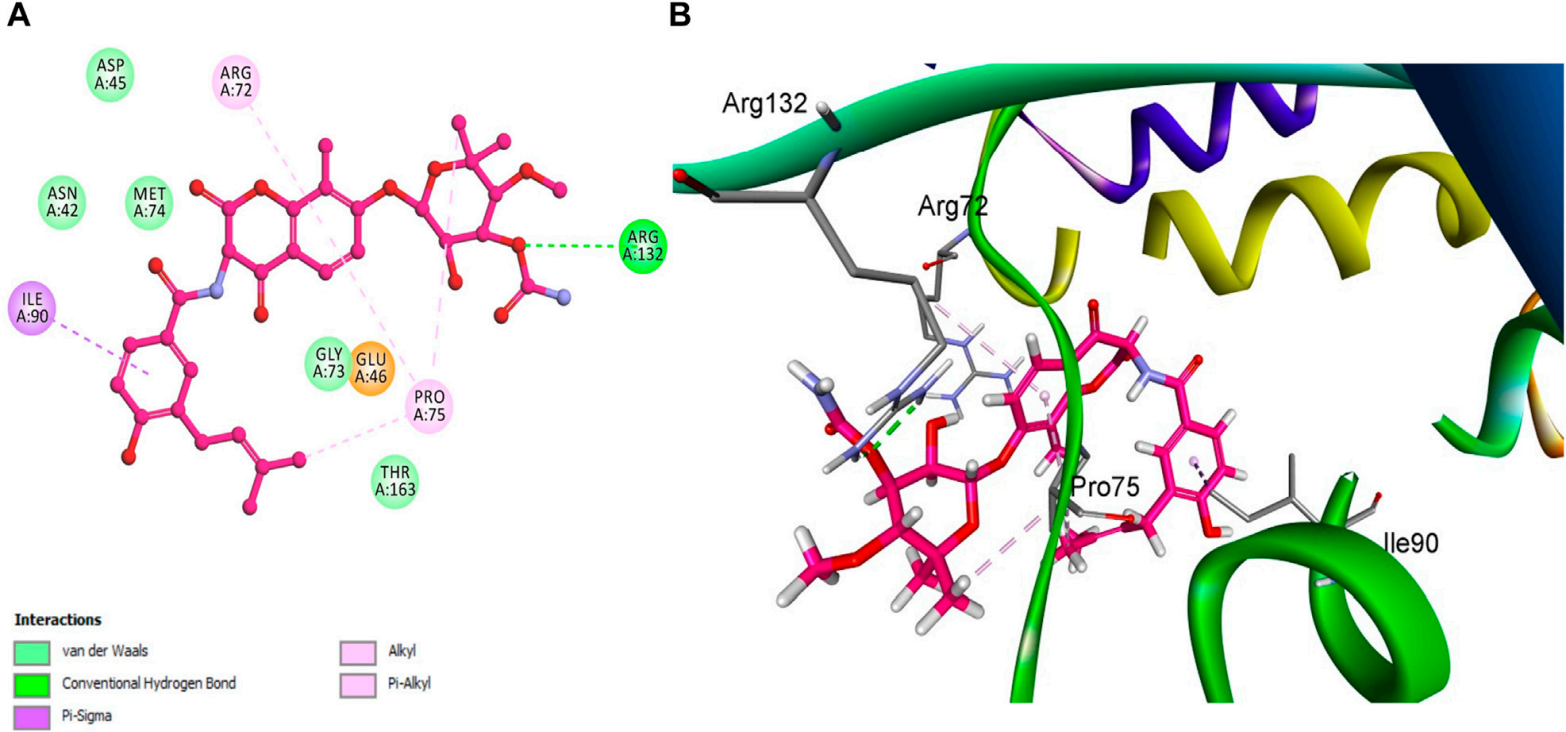
Docking representation models of Novobiocin within the binding site *E. coli* topoisomerase IV; **(A)** 2D-docked model of Novobiocin; **(B)** 3D-docked model of Novobiocin.

In a similar vein, Novobiocin demonstrated notable interactions with *E. coli* topoisomerase IV, particularly involving Arg 132, as depicted in Figure 11. However, the quantum of significant hydrogen bonding interactions was fewer than those observed for **5h**, resulting in a relatively lower affinity of Novobiocin for *E. coli* topoisomerase IV.

**FIGURE 12:**
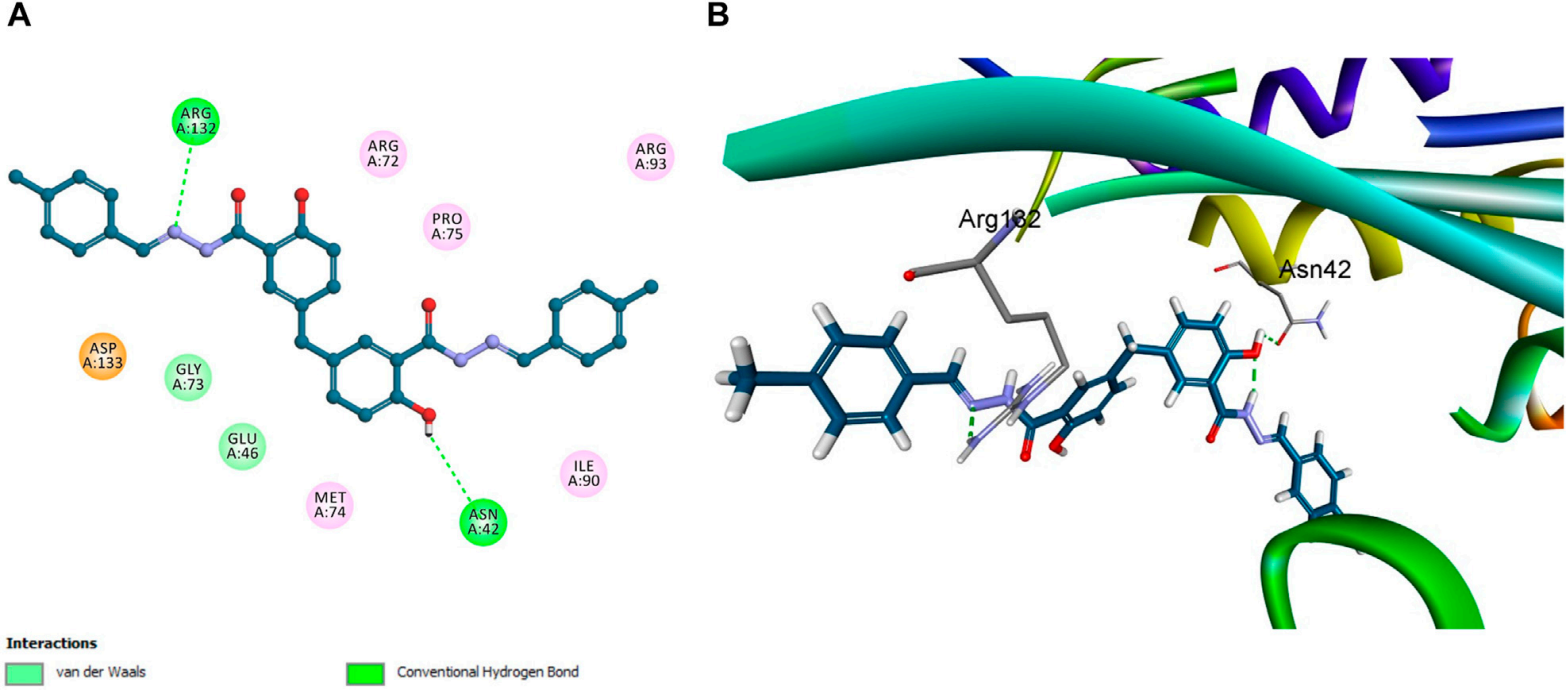
Docking representation models of compound **5f** within the binding site *E. coli* topoisomerase IV; **(A)** 2D-docked model of compound **5f**; **(B)** 3D-docked model of compound **5f**.

Conversely, the molecular structure of compound **5f** was characterized by the absence of multiple significant interactions that were pivotal in the enhanced binding stability of **5h** within the active site. This absence and the lowest docking score among the compounds studied positioned compound **5f** as the least effective binder to *E. coli* topoisomerase IV, as depicted in Figure 12.

This observation was corroborated by experimental *in vitro* assay results, which reported a residual activity of 77% for **5f**, contrasted with IC_50_ values of 3.50 ± 0.30 µM for **5h** and 11 ± 2 µM for Novobiocin. These findings affirm a direct correlation between the molecular interaction profiles, docking scores, and the biological activities of the compounds, illustrating the critical role of specific binding interactions in mediating the efficacy of compound engagement with *E. coli* topoisomerase IV active site. This modeling study against *E. coli* DNA gyrase B and topoisomerase IV enzymes has crucial implications for antibacterial drug development. The study provides valuable insights into how molecular interactions influence compound efficacy by detailing compounds **5f**, **5h**, and Novobiocin binding interactions with these key bacterial enzymes. Particularly, the favorable docking poses of **5h** suggest its potential as a lead for developing new antibiotics. The study highlights the importance of specific interactions, such as hydrogen bonds and pi-stacking, in achieving high specificity and potency, pointing towards strategies for enhancing drug effectiveness. The findings pave the way for the rational design of antibacterial agents, allowing for targeted improvements in drug-binding characteristics. This approach promises more efficient drug development processes and offers a pathway to combat antibiotic resistance by developing novel agents with improved efficacy against bacterial targets.

### 2.4 ADMET studies

This study aims to design and synthesize compounds with improved ADMET characteristics, emphasizing achieving favorable plasma protein binding, absorption, and solubility profiles while ensuring minimal BBB penetration and no inhibition of the CYP2D6 enzyme. The objective will be to develop a compound that has an optimal balance between being sufficiently lipophilic to cross cell membranes and hydrophilic enough to remain soluble in the aqueous environment of the gastrointestinal tract for absorption.

The Polar Surface Area (PSA) across the series of synthesized compounds (**5a-k**) remains conducive to good membrane permeability, a critical attribute in oral drug absorption. Compound **5c**, despite its slightly higher PSA, may offer a balance between solubility and permeability due to its “No” for plasma protein binding (PPB), which suggests a lower likelihood of drug-drug interactions and possibly a shorter half-life, which could be beneficial depending on the desired dosing regimen (Table 4). All synthesized compounds have a PPB value marked as “Yes,” indicating more than 90% plasma protein binding, except for compound **5c**. High PPB may lead to less free drug available for activity but may also provide a depot effect for sustained drug release. The absorption level indicated here spans “2″for most compounds, which are classified as poor, which is a potential concern for oral bioavailability.

**TABLE 4:** Comprehensive prediction of the absorption, distribution, metabolism, excretion, and toxicity (ADME) profiles of synthesized hybrids **5a-k** compared to Novobiocin.

Comp. ID	PSA	PPB^a^	Absorption level^b^	CYP2D6 prediction^c^	BBB level^d^	Solubility level^e^	AlogP98
**5a**	124.499	Yes	2	No	4	2	5.027
**5b**	124.499	Yes	2	No	4	1	6.356
**5c**	210.145	No	3	No	4	2	4.816
**5d**	124.499	Yes	2	No	4	1	6.524
**5e**	124.499	Yes	2	No	4	1	6.356
**5f**	124.499	Yes	2	No	4	2	5.999
**5g**	124.499	Yes	2	No	4	1	6.356
**5h**	142.359	Yes	2	No	4	2	4.994
**5i**	131.204	Yes	2	No	4	2	5.351
**5j**	142.359	Yes	2	No	4	2	4.994
**5k**	124.499	Yes	2	Yes	4	2	5.438
Nov	194.834	No	3	No	4	1	3.696

^**a**^ PPB, plasma protein binding, No means less than 90%, Yes means > 90%.

^b^ Absorption level, 0 = good, 1 = moderate, 2 = poor, 3 = very poor.

^c^ CYP2D6, cytochrome P2D6, Yes = inhibitor, No = non inhibitor.

^d^ BBB, level, blood–brain barrier level, 0 = very high, 1 = high, 2 = medium, 3 = low, 4 = very low.

^e^ Solubility level, 1 = very low, 2 = low, 3 = good, 4 = optimal.

**FIGURE 13:**
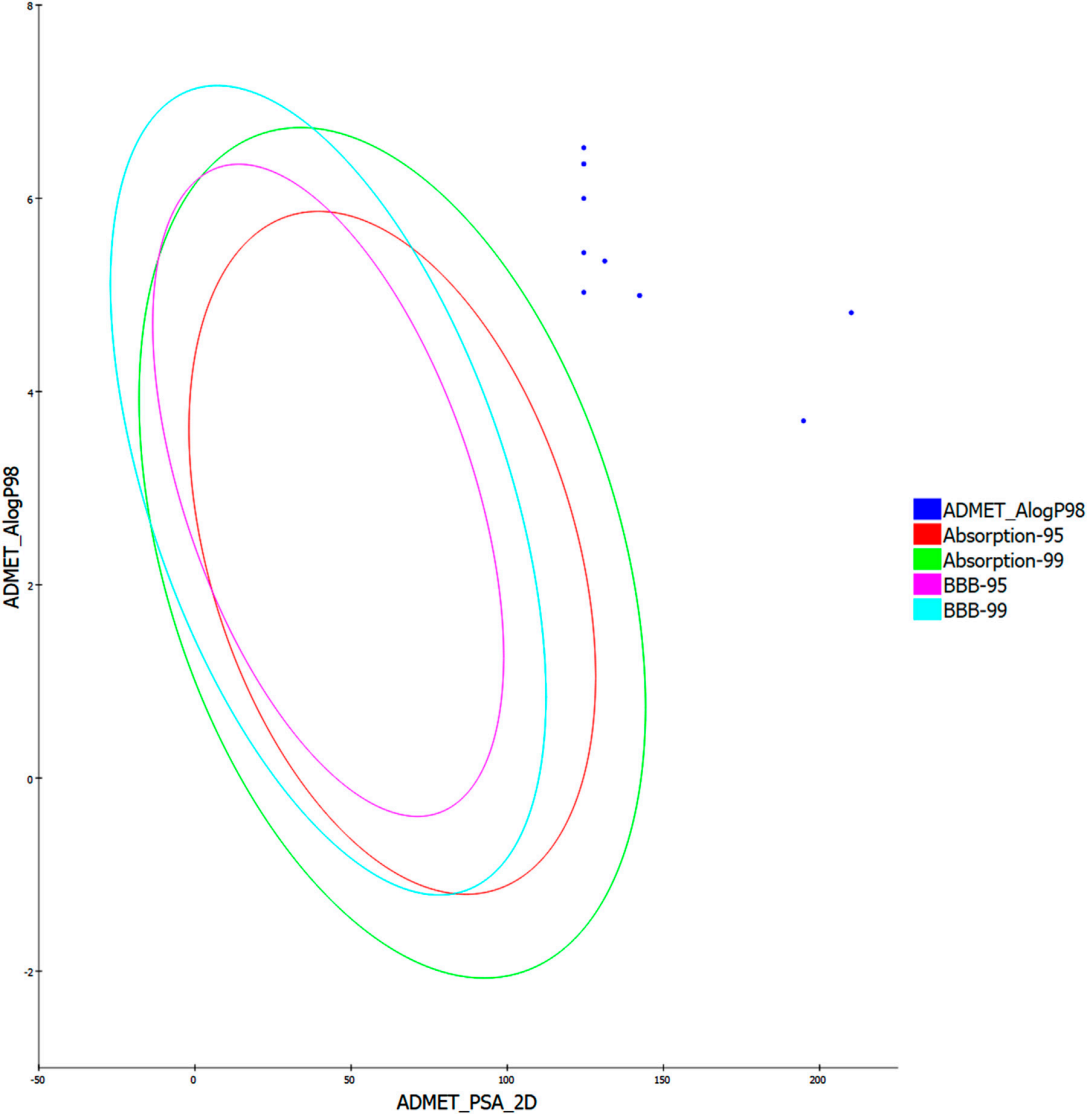
The predicted ADME study of hybrids **5a-k**.

Compound **5c**, however, is rated at “3,” suggesting very poor absorption, which may necessitate further structural modification to improve this property. None of the compounds, including Novobiocin, are predicted to be CYP2D6 inhibitors, which is positive as it reduces the risk of metabolic drug-drug interactions involving this common enzyme pathway. The Blood-Brain Barrier (BBB) Level for all compounds is “4,” indicating very low penetration. This is beneficial for avoiding central nervous system side effects if the therapeutic target is peripheral. Solubility Levels for most compounds are categorized as “2,” indicating low solubility, with Novobiocin at “1,” suggesting very low solubility. Solubility is a key factor for drug absorption and may need to be optimized for these compounds. The AlogP98 scores, which indicate lipophilicity, vary, with most synthesized compounds showing higher values than Novobiocin (Figure 13).

While this may be beneficial for membrane permeation, it may also impact the solubility and distribution of the compounds. The synthesized compounds are promising candidates for further optimization. Given their high plasma protein binding and the poor absorption levels indicated, future work may focus on modifying these compounds to enhance their solubility and absorption. This study is a significant step toward developing new antibacterial agents with enhanced ADME properties. Future efforts may include applying prodrug strategies, using novel delivery systems, or further molecular modification to balance the physicochemical properties and optimize the pharmacokinetic profile of these compounds. The ultimate aim is to deliver new drugs to market that are safer, more effective, and capable of addressing the urgent need for novel antibiotics in the face of rising drug resistance.

## 3 Conclusion

Eleven compounds (**5a-k**) were developed by reacting disalicylic acid methylene hydrazide with different aldehydes. The antibacterial activity of newly synthesized compounds **5a-k** was tested against various gram-negative and gram-positive bacterial strains and fungal species. These new targets were investigated for DNA gyrase and topoisomerase IV inhibitory activity. Compounds **5h** and **5i** had the highest activity level in enzymatic tests. Compound **5h** exhibited an IC_50_ value of 92 ± 5 nM against *E. coli* DNA gyrase, while compound **5i** showed an IC_50_ value of 97 ± 6 nM against the same target. In addition, they exhibited inhibitory effects on *E. coli* topoisomerase IV, with IC_50_ values of 3.50 ± 0.30 µM and 5.80 ± 0.40 µM, respectively. Compounds **5h** and **5i** showed strong antibacterial action against gram-positive bacteria, including *S. aureus*, with MIC values of 0.030 and 0.035 μg/mL. As a result, compounds **5h** and **5i** show promise for future optimization since they have low-MIC values against gram-positive bacteria and low-nanomolar *in vitro* enzymatic activity against topoisomerase IV and DNA gyrase. Docking studies demonstrate compound **5h** as potent inhibitors of *E. coli* DNA gyrase B and topoisomerase IV, showing favorable docking poses. ADMET profile highlighted the need for solubility and absorption optimization while avoiding central nervous system side effects. The research underscores the potential of these compounds as bases for developing new antibacterial agents with improved efficacy and pharmacokinetic properties.

### 3.1 Future perspectives

Antimicrobial resistance continues to be a significant peril to worldwide health in the 21st century. Therefore, it is imperative for researchers and pharmaceutical companies to collaborate in order to combat these lethal superbugs, united by a same objective: Complete eradication of bacterial diseases worldwide. The main cause of antimicrobial resistance is mutations in the subunits of DNA gyrase. Therefore, employing a molecular hybridization strategy using the structures reported in this study has the potential to develop more potent DNA gyrase and/or Topoisomerase IV inhibitors. Furthermore, the lead compounds identified in this investigation can be combined with other molecular agents, like various heterocycle moieties, to enhance their effectiveness against numerous infectious microorganisms.

## 4 Experimental

### 4.1 Chemistry

#### 4.1.1 Synthesis of 5,5′-methylenebis (3-chloro-2-hydroxybenzoic acid), compound 2

3-Chlorosalicylic acid **1** (25.0 g, 144.83 mmol, 2.0 equiv) and paraformaldehyde (2.17 g, 72.42 mmol, 1.0 equiv) were dissolved in concentrated sulfuric acid (150 mL). The reaction mixture was stirred at 35 ^o^C for 18 h. The solution was then poured over crushed ice, and the white precipitate was filtered off, rinsed with ice-cold water, and dried *in vacuo* to afford the desired compound **2** as a white powder. The product can be extracted with diethyl ether and washed with water to remove residual sulfuric acid.

Yield: 25.30 g (98%), white solid, m. p: >300 °C. ^1^H NMR (400 MHz, δ ppm CD_3_OD): 7.67 (d, *J* = 2.2 Hz, 2H, 6,6′Ar-H), 7.45 (d, *J* = 2.2 Hz, 2H, 4,4′Ar-H), 3.87 (s, 2H, benzylic CH_2_). ^13^C NMR (100 MHz, *δ* ppm DMSO*-d*_6_): 171.7, 156.0, 135.5, 131.8, 128.5, 121.5, 113.8, 38.5. HRMS (*m/z*) calcd. for C_15_H_10_Cl_2_O_6_: 355.9854; Found: 354.9853 [M-H]^-^.

#### 4.1.2 Synthesis of 5,5′-methylenebis (2-hydroxybenzoic acid), compound 3

To a 1,000 mL Schlenk flask fitted with a magnetic stir bar, Compound **2** (20.0 g, 55.9 mmol) and 10% Pd/C (5.00 g, 25% w/w relative to **2**) were added. The flask was then purged with nitrogen, and the solid mixture was dissolved in 200 mL 2-PrOH, and slowly added down the sides of the reaction vessel under positive nitrogen gas flow. While stirring, 50.0 mL of a 4.5 M aqueous solution of KHCOO (225 mmol, 4 equiv.) was added slowly under the positive nitrogen gas flow, forming a white precipitate. To this slurry was then added 200 mL of deionized water, followed by the slow addition of KOH pellets (100 g, 1.78 mol, 32 equiv.) while stirring until the precipitate dissolved. The reaction was stirred at 70 °C for 4 h, and an additional 25.0 mL portion of the 4.5 M KHCOO solution was added at 2 h.

UHPLC analysis of the reaction aliquots found 99.7% dehalogenation at time = 2 h. The reaction mixture was filtered through glass frit layered glass microfiber filter pads and glass wool. The Pd/C was then rinsed with two 100 mL portions of deionized water. The filtrate was transferred to a two-liter beaker, cooled in an ice bath, and diluted to 1.5 L with deionized water. A stir bar was added, and the solution was acidified to pH < 4 with concentrated HCl. The precipitate was filtered off and washed with three portions of deionized water (100 mL). Due to the high ionic strength of the filtrate, some potassium chloride may precipitate out along with the product during acidification. Inorganic materials in the product can be removed by dissolving the product in diethyl ether and extracting it with water. The combined extracts were dried over sodium sulfate, and the solvent was removed by rotary evaporation. The resulting solid was dried under reduced pressure to yield **3** as a white solid.

Yield: 14.90 g (92%), white solid, m. p: >300 °C. ^1^H NMR (400 MHz, δ ppm DMSO-*d*_6_): 7.62 (d, *J* = 2.2 Hz, 2H, 6,6′Ar-H), 7.37 (dd, *J* = 8.5 and 2.2 Hz, 2H, 4,4′Ar-H), 6.89 (d, *J* = 8.5 Hz, 2H, 3,3′Ar-H) 3.85 (s, 2H, benzylic CH_2_). ^13^C NMR (100 MHz, *δ* ppm DMSO*-d*_6_): 172.0, 160.2, 135.8, 131.6, 129.8, 116.9, 112.2, 39.2. HRMS (*m/z*) calcd. for C_15_H_12_O_6_: 288.0634; Found: 287.0651 [M-H]^-^.

#### 4.1.3 Synthesis of 5,5′-methylenebis (2-hydroxybenzohydrazide), compound 4

A catalytic amount of concentrated H_2_SO_4_ was mixed into a stirred solution of 4,4′-methylenebis (2-acetoxybenzoic acid) (**III**) (5 g, 17.35 mmol) in 200 mL of absolute ethanol. The mixture was refluxed while the reaction was monitored using TLC until the acid became esterified. The reaction solvent was evaporated under reduced pressure, and the crude ester product proceeded to the next step without purification. The crude ester was mixed in 50 mL of absolute ethanol with an excess of hydrazine hydrate for 30 min, refluxed for 6 h (TLC monitored the reaction), and cooled to room temperature. The resulting product was often filtered and washed with ethanol to get the hydrazide product.

Yield: 3.25 g (74%), white solid, m. p: 260–263 °C, R_*f*_: 0.2 (chloroform: methanol, 9:1, v/v). (FTIR): ν_max_ (cm^−1^) 2,500–3,400 (phenolic OH), 3,333, 3,195 (NH_2_), 2,922, 2,848 (CH), 1,633 (amidic C=O), 1,602, 1,574, (C = C), 824 and 698 (Ar–CH bending). ^1^H NMR (400 MHz, δ ppm DMSO-*d*_6_): 12.08 (s, 2H, phenolic-OH), 9.95 (s, 2H, amidic-NH), 7.68 (d, *J* = 2 Hz, 2H, Ar–H), 7.19 (2 d, *J* = 8.4 Hz, 2 Hz, 2H, Ar–H), 6.81 (d, *J* = 8.4 Hz, 2H, Ar–H), 4.62 (s, 4H, C=O-NH-NH_2_), 3.74 (s, 2H, Ar–CH_2_).

#### 4.1.4 General procedure for the synthesis of compounds (5a-k)

To a stirred solution of 4,4′-methylenebis (2-hydroxybenzohydrazide) (**III**) (0.32 mmol, 1eq) in 30 mL of absolute ethanol using a few drops of glacial acetic acid as a catalyst, appropriate aromatic aldehyde (**VIa-k**) (**0.95 mmol, 3 eq**) was added slowly, and the resultant mixture was refluxed overnight. After the reaction was completed (monitored with TLC), the reaction was allowed to be cooled, and the precipitate was filtered and washed several times with ethanol to get rid of excess unreacted aldehyde.

##### 4.1.4.1 *N*'-((*E*)-Benzylidene)-4-(4-(2-((*Z*)-benzylidene)hydrazine-1-carbonyl)-3-hydroxy benzyl)-2-hydroxybenzohydrazide (5a)

Yield: 0.13 g (83%), white solid, m. p: >300 °C, R_*f*_: 0.38 (chloroform: methanol, 9:1, v/v). ^1^H NMR (400 MHz, δ ppm DMSO-*d*_6_): 11.74 (s, 2H, phenolic-OH), 8.47 (s, 2H, amidic-NH), 7.82–7.62 (m, 5H, Ar–H, 2H, CH = N), 7.47 (d, *J* = 6.1 Hz, 6H, Ar–H), 7.32–7.17 (m, 3H, Ar–H), 6.93 (t, *J* = 7.8 Hz, 2H, Ar–H), 3.90 (s, 2H, Ar–CH_2_). LCMS (*m/z*) calcd. for C_29_H_24_N_4_O_4_: 492.18; Found: 493.30 [M + H]^+^. Anal. Calc. (%) for C_29_H_24_N_4_O_4_: C, 70.72; H, 4.91; N, 11.38. Found: C, 72.98; H, 5.15; N, 11.50.

##### 4.1.4.2 *N*'-((*E*)-4-Chlorobenzylidene)-4-(4-(2-((*Z*)-4-chlorobenzylidene)hydrazine-1-carbonyl)-3-hydroxybenzyl)-2-hydroxybenzohydrazide (5b)

Yield: 0.12 g (68%), white solid, m. p: >300 °C, R_*f*_: 0.42 (chloroform: methanol, 9:1, v/v). ^1^H NMR (400 MHz, δ ppm DMSO-*d*_6_): 11.78 (s, 2H, phenolic-OH), 8.46 (s, 2H, amidic-NH), 7.78–7.72 (m, 2H, CH = N, 5H, Ar–H), 7.53 (d, *J* = 5.2 Hz, 4H, Ar–H), 7.28 (d, *J* = 7.3 Hz, 3H, Ar–H), 6.93 (d, *J* = 5.7 Hz, 2H, Ar–H), 3.90 (s, 2H, Ar–CH_2_). LCMS (*m/z*) calcd. for C_29_H_22_Cl_2_N_4_O_4_: 560.10; Found: 561.20 [M + H]^+^. Anal. Calc. (%) for C_29_H_22_C_l2_N_4_O_4_: C, 62.04; H, 3.95; N, 9.98. Found: C, 62.25; H, 4.09; N, 10.25.

##### 4.1.4.3 *N*'-((*E*)-4-Nitrobenzylidene)-4-(4-(2-((*Z*)-4-nitrobenzylidene)hydrazine-1-carbonyl)-3-hydroxybenzyl)-2-hydroxybenzohydrazide (5c)

Yield: 0.14 g (76%), white solid, m. p: >300 °C, R_*f*_: 0.48 (chloroform: methanol, 9:1, v/v). ^1^H NMR (400 MHz, δ ppm DMSO-*d*_6_): 11.94 (s, 2H, phenolic-OH), 8.56 (s, 2H, amidic-NH), 8.30 (d, *J* = 7.3 Hz, 4H, Ar–H), 8.00 (d, *J* = 5.1 Hz, 4H, Ar–H), 7.72 (d, *J* = 9 Hz, 2H, Ar–H), 7.31–7.22 (m, 2H, CH = N, 2H, Ar–H), 6.93 (d, *J* = 7.5 Hz, 2H, Ar–H), 3.83 (s, 2H, Ar–CH_2_). LCMS (*m/z*) calcd. for C_29_H_22_N_6_O_8_: 582.15; Found: 583.30 [M + H]^+^. Anal. Calc. (%) for C_29_H_22_N_6_O_8_: C, 59.79; H, 3.81; N, 14.43. Found: C, 60.01; H, 3.97; N, 14.61.

##### 4.1.4.4 *N*'-((*E*)-4-Bromobenzylidene)-4-(4-(2-((*Z*)-4-bromobenzylidene)hydrazine-1-carbonyl)-3-hydroxybenzyl)-2-hydroxybenzohydrazide (5d)

Yield: 0.15 g (75%), white solid, m. p: >300 °C, R_*f*_: 0.45 (chloroform: methanol, 9:1, v/v). ^1^H NMR (400 MHz, δ ppm DMSO-*d*_6_): 11.78 (s, 2H, phenolic-OH), 8.44 (s, 2H, amidic-NH), 7.78 (s, 2H, CH = N), 7.72–7.60 (m, 8H, Ar–H), 7.28 (d, *J* = 7.8 Hz, 3H, Ar–H), 6.93 (d, *J* = 6.7 Hz, 3H, Ar–H), 3.90 (s, 2H, Ar–CH_2_). LCMS (*m/z*) calcd. for C_29_H_22_Br_2_N_4_O_4_: 650.32; Found: 651.20 [M + H]^+^. Anal. Calc. (%) for C_29_H_22_Br_2_N_4_O_4_: C, 53.56; H, 3.41; N, 8.62. Found: C, 53.78; H, 3.54; N, 8.89.

##### 4.1.4.5 *N*'-((*E*)-2-Chlorobenzylidene)-4-(4-(2-((*Z*)-2-chlorobenzylidene)hydrazine-1-carbonyl)-3-hydroxybenzyl)-2-hydroxybenzohydrazide (5e)

Yield: 0.11 g (62%), white solid, m. p: >300 °C, R_*f*_: 0.42 (chloroform: methanol, 9:1, v/v). ^1^H NMR (400 MHz, δ ppm DMSO-*d*_6_): 11.98 (s, 2H, phenolic-OH), 8.86 (s, 2H, amidic-NH), 8.04 (d, *J* = 6.5 Hz, 2H, Ar–H), 7.80 (s, 2H, CH = N), 7.53 (d, *J* = 7.5 Hz, 4H, Ar–H), 7.46 (d, *J* = 6 Hz, 4H, Ar–H), 7.30–7.23 (m, 2H, Ar–H), 7.28 (d, *J* = 7 Hz, 2H, Ar–H), 3.91 (s, 2H, Ar–CH_2_). LCMS (*m/z*) calcd. for C_29_H_22_Cl_2_N_4_O_4_: 560.10; Found: 561.20 [M + H]^+^. Anal. Calc. (%) for C_29_H_22_Cl_2_N_4_O_4_: C, 62.04; H, 3.95; N, 9.98. Found: C, 61.97; H, 4.12; N, 10.07.

##### 4.1.4.6 *N*'-((*E*)-4-Methylbenzylidene)-4-(4-(2-((*Z*)-4-methylbenzylidene)hydrazine-1-carbonyl)-3-hydroxybenzyl)-2-hydroxybenzohydrazide (5f)

Yield: 0.1 g (61%), white solid, m. p: >300 °C, R_*f*_: 0.3 (chloroform: methanol, 9:1, v/v). ^1^H NMR (400 MHz, δ ppm DMSO-*d*_6_): 11.70 (s, 2H, amidic-NH), 8.43 (s, 2H, phenolic-OH), 7.80 (s, 2H, CH = N), 7.75–7.59 (m, 5H, Ar–H), 7.28 (d, *J* = 7 Hz, 7H, Ar–H), 6.92 (d, *J* = 7.2 Hz, 2H, Ar–H), 3.89 (s, 2H, Ar–CH_2_), 2.85 (s, 6H, Ar–CH_3_). LCMS (*m/z*) calcd. for C_31_H_28_N_4_O_4_: 520.20; Found: 521.30 [M + H]^+^. Anal. Calc. (%) for C_31_H_28_N_4_O_4_: C, 71.52; H, 5.42; N, 10.76. Found: C, 71.34; H, 5.66; N, 10.97.

##### 4.1.4.7 *N*'-((*E*)-3-Chlorobenzylidene)-4-(4-(2-((*Z*)-3-chlorobenzylidene)hydrazine-1-carbonyl)-3-hydroxybenzyl)-2-hydroxybenzohydrazide (5 g)

Yield: 0.12 g (67%), white solid, m. p: >300 °C, R_*f*_: 0.42 (chloroform: methanol, 9:1, v/v). ^1^H NMR (400 MHz, δ ppm DMSO-*d*_6_): 11.83 (s, 2H, phenolic-OH), 8.45 (s, 2H, amidic-NH), 7.81–7.67 (m, 2H, CH = N, 5H, Ar-H), 7.55–7.45 (m, 5H, Ar-H), 7.28 (d, *J* = 7.7 Hz, 2H, Ar–H), 6.93 (d, *J* = 6.7 Hz, 2H, Ar–H), 3.91 (s, 2H, Ar–CH_2_). LCMS (*m/z*) calcd. for C_29_H_22_Cl_2_N_4_O_4_: 560.10; Found: 561.23 [M + H]^+^. Anal. Calc. (%) for C_29_H_22_Cl_2_N_4_O_4_: C, 62.04; H, 3.95; N, 9.98. Found: C, 62.29; H, 4.08; N, 10.12.

##### 4.1.4.8 *N*'-((*E*)-2-Methoxybenzylidene)-4-(4-(2-((*Z*)-2-methoxybenzylidene)hydrazine-1-carbonyl)-3-hydroxybenzyl)-2-hydroxybenzohydrazide (5 h)

Yield: 0.13 g (73%), white solid, m. p: 288–290 °C, R_*f*_: 0.35 (chloroform: methanol, 9:1, v/v). ^1^H NMR (400 MHz, δ ppm DMSO-*d*_6_): 11.86 (s, 2H, phenolic-OH), 8.80 (s, 2H, amidic-NH), 7.88 (2 d, *J* = 6.4 Hz, 1.3 Hz, 3H, Ar-H), 7.84 (s, 2H, CH = N), 7.46–7.42 (m, 2H, Ar–H), 7.28 (2 d, *J* = 6.7 Hz, 1.8 Hz, 2H, Ar-H), 7.11 (d, *J* = 8.3 Hz, 2H, Ar–H), 7.03 (t, *J* = 7.5 Hz, 3H, Ar–H), 6.91 (d, *J* = 8.4 Hz, 2H, Ar–H), 3.88 (s, 2H, Ar–CH_2_), 3.85 (s, 6H, Ar–OCH_3_). LCMS (*m/z*) calcd. for C_31_H_28_N_4_O_6_: 552.20; Found: 553.30 [M + H]^+^. Anal. Calc. (%) for C_31_H_28_N_4_O_6_: C, 67.38; H, 5.11; N, 10.14. Found: C, 67.51; H, 5.23; N, 10.41.

##### 4.1.4.9 *N*'-((*E*)-4-(Dimethylamino)benzylidene)-4-(4-(2-((*Z*)-4-(dimethylamino) benzylidene)hydrazine-1-carbonyl)-3-hydroxybenzyl)-2-hydroxybenzohydrazide (5i)

Yield: 0.16 g (87%), yellow-orange, m. p: 298–300 °C, R_*f*_: 0.38 (chloroform: methanol, 9:1, v/v). ^1^H NMR (400 MHz, δ ppm DMSO-*d*_6_): 11.58 (s, 2H, phenolic-OH), 8.31 (s, 2H, amidic-NH), 7.81 (s, 2H, CH = N), 7.56 (d, *J* = 8.7 Hz, 4H, Ar–H), 7.26 (2 d, *J* = 6.7 Hz, 1.7 Hz, 3H, Ar-H), 6.91 (d, *J* = 8.4 Hz, 2H, Ar–H), 6.76 (t, *J* = 8.7 Hz, 5H, Ar–H), 3.88 (s, 2H, Ar–CH_2_), 2.98 (s, 12H, Ar–N(CH_3_)_2_). LCMS (*m/z*) calcd. for C_33_H_34_N_6_O_4_: 578.26; Found: 579.50 [M + H]^+^.Anal. Calc. (%) for C_33_H_34_N_6_O_4_: C, 68.50; H, 5.92; N, 14.52. Found: C, 68.76; H, 6.05; N, 14.67.

##### 4.1.4.10 *N*'-((*E*)-4-Methoxybenzylidene)-4-(4-(2-((*Z*)-4-methoxybenzylidene)hydrazine-1-carbonyl)-3-hydroxybenzyl)-2-hydroxybenzohydrazide (5j)

Yield: 0.1 g (58%), white solid, m. p: >300 °C, R_*f*_: 0.35 (chloroform: methanol, 9:1, v/v). ^1^H NMR (400 MHz, δ ppm DMSO-*d*_6_): 11.64 (s, 2H, phenolic-OH), 8.41 (s, 2H, amidic-NH), 7.80 (s, 2H, CH = N), 7.77–7.66 (m, 4H, Ar-H), 7.30–7.22 (m, 3H, Ar–H), 7.03 (d, *J* = 6.8 Hz, 4H, Ar-H), 6.92 (d, *J* = 7 Hz, 3H, Ar–H), 3.89 (s, 2H, Ar–CH_2_), 3.82 (s, 6H, Ar–OCH_3_). LCMS (*m/z*) calcd. for C_31_H_28_N_4_O_6_: 552.20; Found: 553.30 [M + H]^+^. Anal. Calc. (%) for C_31_H_28_N_4_O_6_: C, 67.38; H, 5.11; N, 10.14. Found: C, 67.25; H, 5.07; N, 10.35.

##### 4.1.4.11 *N*'-((*E*)-4-Fluorobenzylidene)-4-(4-(2-((*Z*)-4-fluorobenzylidene)hydrazine-1-carbonyl)-3-hydroxybenzyl)-2-hydroxybenzohydrazide (5k)

Yield: 0.11 g (73%), white solid, m. p: >300 °C, R_*f*_: 0.47 (chloroform: methanol, 9:1, v/v). ^1^H NMR (400 MHz, δ ppm DMSO-*d*_6_): 11.73 (s, 2H, phenolic-OH), 8.46 (s, 2H, amidic-NH), 7.81 (s, 2H, CH = N), 7.79–7.73 (m, 4H, Ar-H), 7.34–7.22 (m, 6H, Ar–H), 6.93 (d, *J* = 7.2 Hz, 2H, Ar-H), 6.84 (d, *J* = 8 Hz, 2H, Ar–H), 3.83 (s, 2H, Ar–CH_2_). LCMS (*m/z*) calcd. for C_29_H_22_F_2_N_4_O_4_: 528.16; Found: 529.30 [M + H]^+^. Anal. Calc. (%) for C_29_H_22_F_2_N_4_O_4_: C, 65.91; H, 4.20; N, 10.60. Found: C, 66.13; H, 4.36; N, 10.81.

### 4.2 Biology

#### 4.2.1 Antibacterial assay

The antibacterial activity of compounds **5f**, **5h**, **5i**, and **5k** were evaluated using a twofold serial dilution method (Parasa et al., 2011), with ciprofloxacin as the reference compound. The MIC values for certain compounds were obtained through dose-response experiments. The stated values are based on a minimum of two independent experiments, with three replicates per concentration in each experiment. The experimental details can be found in Appendix A (Supplementary Material).

#### 4.2.2 DNA gyrase and topoisomerase IV inhibitory assays

The inhibitory action of compounds **5f**, **5h**, **5i**, and **5k** against DNA gyrase and topoisomerase IV was evaluated using a supercoiling test (Durcik et al., 2018). Inspiralis assay kits were used to measure inhibitory activity against DNA gyrase and topoisomerase IV in *E. coli*, following previously published protocols (Durcik et al., 2018). IC_50_ values were determined using seven different inhibitor concentrations and then calculated using the GraphPad Prism 6.0 software. For the most important inhibitors, IC_50_ values were established using three independent measurements, and the final results provided are mean values. Refer to Appendix A for more details.

#### 4.2.3 Cell viability assay

The **5h** and **5i** viability impact was evaluated using the MCF-10A cell line. After a 4-day incubation period on MCF-10A cells with a concentration of 50 µM for each compound tested, cell viability was measured using the MTT assay (Mekheimer et al., 2022; Mahmoud et al., 2024). Refer to Appendix A for more details.

### 4.3 *In Silico* studies

#### 4.3.1 Docking study

We utilized the BIOVIA Discovery Studio 2021 software (version 21.1.0.20.298) for our molecular docking investigation (Ibrahim et al., 2020; Shaykoon et al., 2020). We employed the Protein Preparation Wizard to ready the chosen proteins for docking analysis. After preparing the protein, we carefully placed the ligands onto a three-dimensional model and performed energy minimization using LigPrep. We utilized the Receptor Grid Generation Tool to create a customized receptor grid specifically designed for the chosen binding location. This was done to enhance potential binding interactions. Afterwards, the Glide tool was used to thoroughly evaluate both docking scores and the various binding modes demonstrated by the ligands.

#### 4.3.2 *In silico* ADMET analysis

In our investigation, ADMET (Absorption, Distribution, Metabolism, Excretion, and Toxicity) experiments were performed utilizing BIOVIA I Discovery Studio 2021 (Mohamed et al., 2019). All compounds’ chemical structures were input, and ADMET descriptors were predicted using integrated models. These models included assessments that were based on Lipinski’s Rule of Five and evaluations of absorption, distribution, metabolism, excretion, and toxicity. The collected data were thoroughly examined to ascertain the drug-like properties and safety profiles of the substances being studied.

## Data Availability

The original contributions presented in the study are included in the article/Supplementary Material, further inquiries can be directed to the corresponding authors.
