# Supplementary material for: Discovery of new Schiff bases of the disalicylic acid scaffold as DNA gyrase and topoisomerase IV inhibitors endowed with antibacterial properties
Source: Front Chem. 2024 Jun 7;12:1419242. doi: 10.3389/fchem.2024.1419242 (PMC11191877; doi:10.3389/fchem.2024.1419242)
Supplement: Supplementary file 1 [file DataSheet1.PDF]

Supplementary data

Discovery of new Schiff bases of the disalicylic acid scaffold as DNA gyrase and Topoisomerase IV inhibitors endowed with antibacterial properties

Lamya H. Al-Wahaibi[1], Mohamed A. Mahmoud[2], Hayat Ali Alzahrani[3], Hesham A. Abou-Zied[4], Hesham A. M. Gomaa[5], Bahaa G. M. Youssif[2]*, Stefan Bräse[6]*, Safwat M. Rabea[7,8]

[1]Department of Chemistry, College of Sciences, Princess Nourah bint Abdulrahman University, Saudi Arabia; [2]Pharmaceutical Organic Chemistry Department, Faculty of Pharmacy, Assiut University, Assiut 71526, Egypt; [3]Applied Medical Science College, Medical Laboratory Technology Department, Northern Border University, Arar, Saudi Arabia; [4]Medicinal Chemistry Department, Faculty of Pharmacy, Deraya University, Minia, Egypt; [5]Pharmacology Department, College of Pharmacy, Jouf University, Sakaka 72314, Saudi Arabia; [6]Institute of Biological and Chemical Systems, IBCS-FMS, Karlsruhe Institute of Technology, 76131 Karlsruhe, Germany, [7]Medicinal Chemistry Department, Faculty of Pharmacy, Minia University, Minia 61519, Egypt; [8]Apogee Pharmaceuticals, 4475 Weyburn Dr, Suite 105, Burnaby, BC V6V2H8, Canada.

**To whom correspondence should be addressed:*

Bahaa G. M. Youssif, Ph.D. Pharmaceutical Organic Chemistry Department, Faculty of Pharmacy, Assiut University, Assiut 71526, Egypt.

Tel.: (002)-01098294419

E-mail address: bahaa.youssif@pharm.aun.edu.eg, bgyoussif@ju.edu.sa

Stefan Bräse

Institute of Biological and Chemical Systems, IBCS-FMS, Karlsruhe Institute of Technology, 76131 Karlsruhe, Germany. E-mail: braese@kit.edu

Figure S1: ^{1}H NMR spectrum of compound **2**

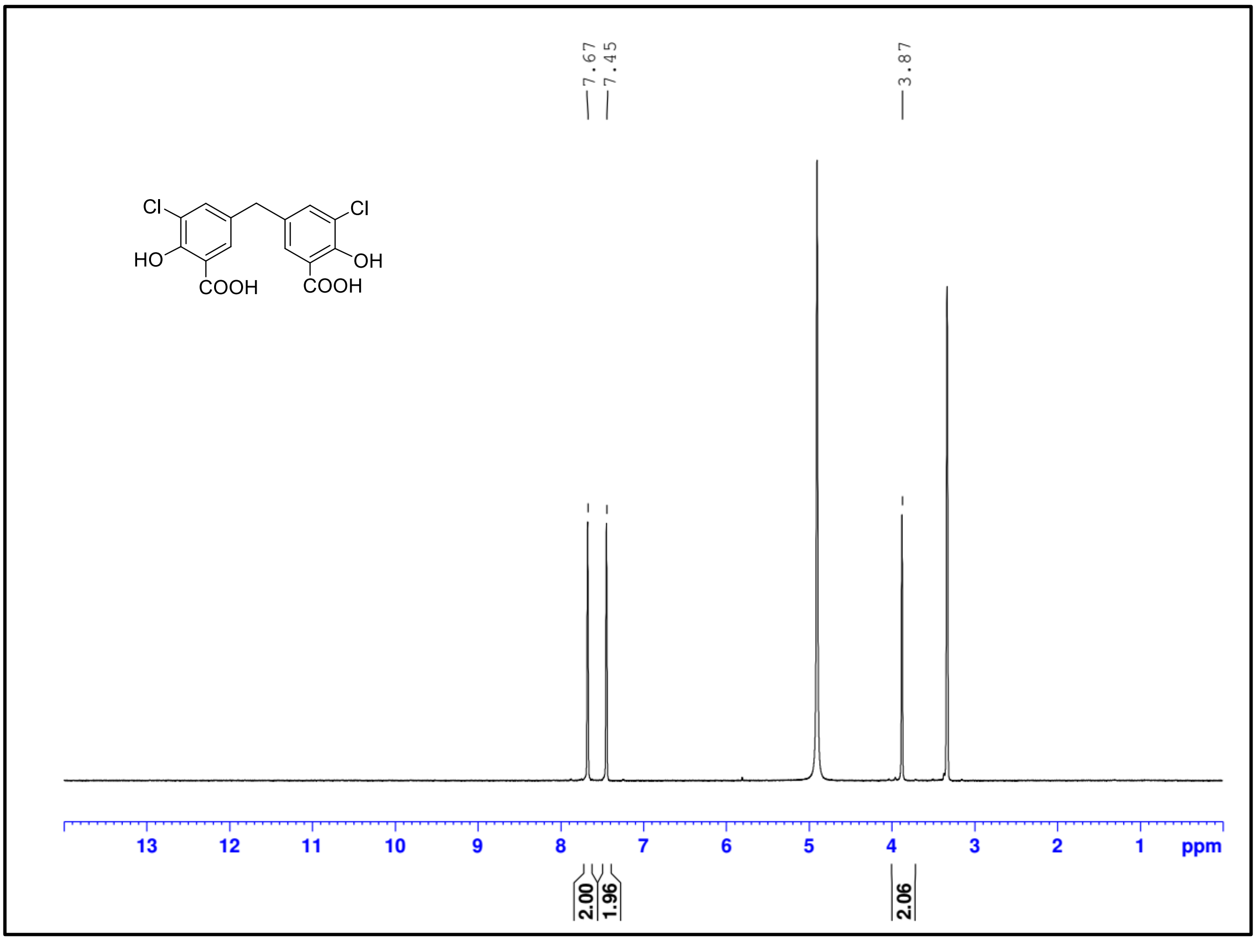

Figure S2: HRMS spectrum of compound **2** showing $[M-H]^-$ peak.

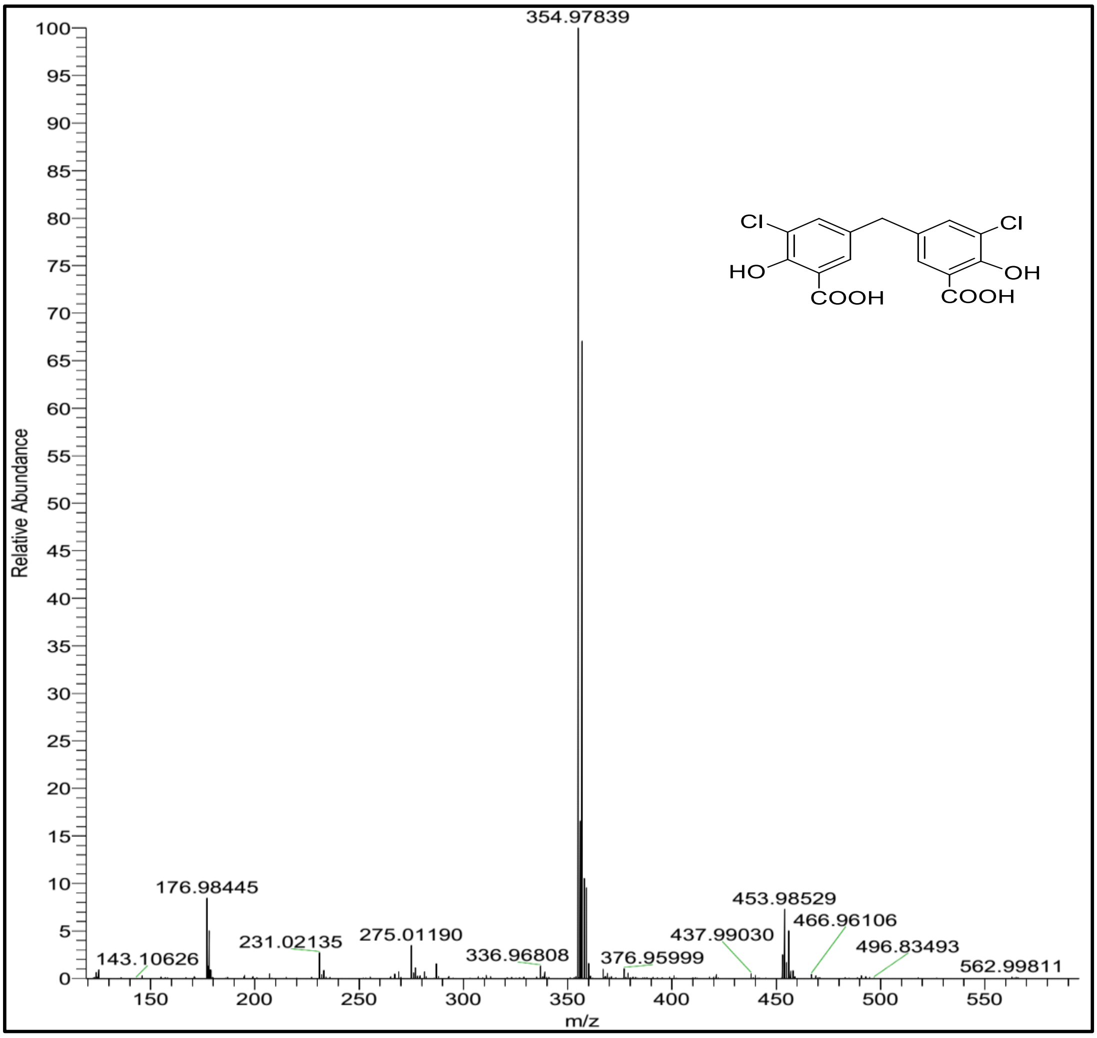

Figure S3: ^{13}C NMR Spectrum of compound **2**

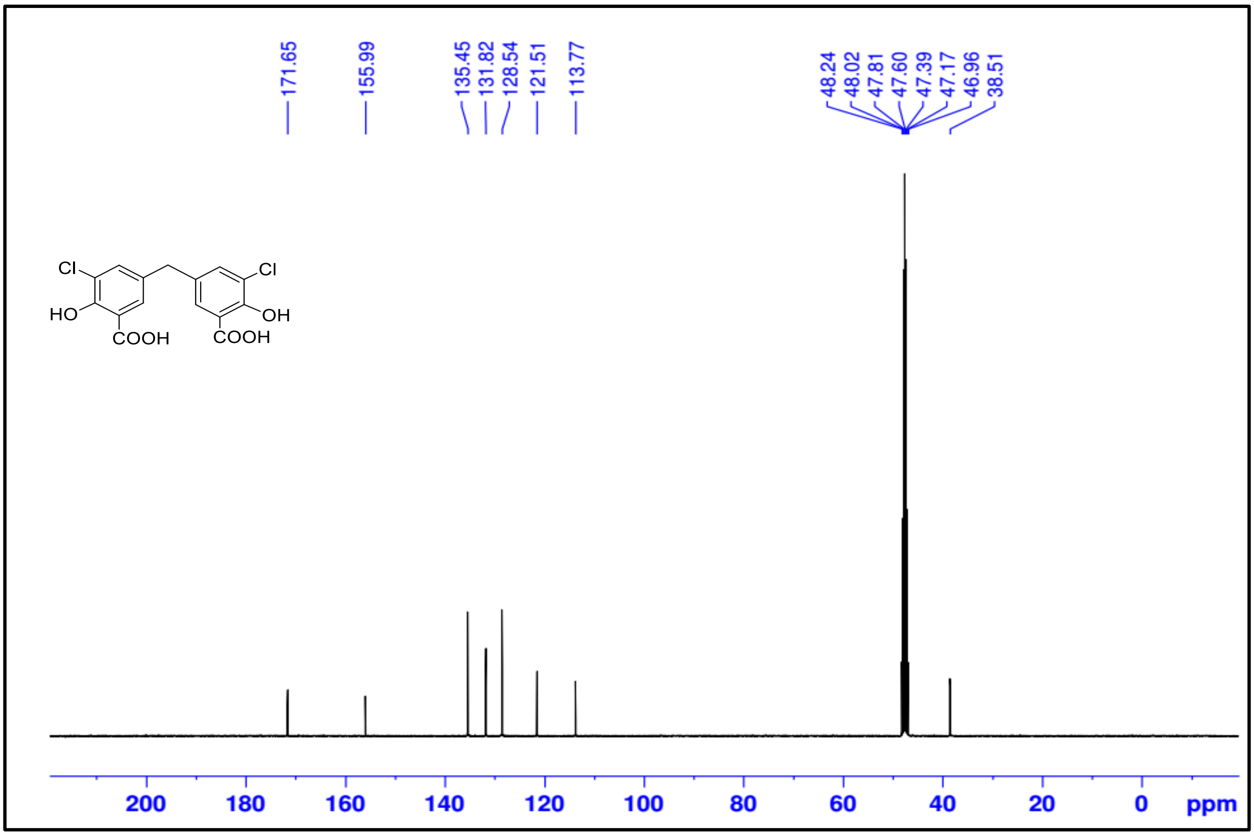

Figure S4: ^{1}H NMR spectrum of compound **3**

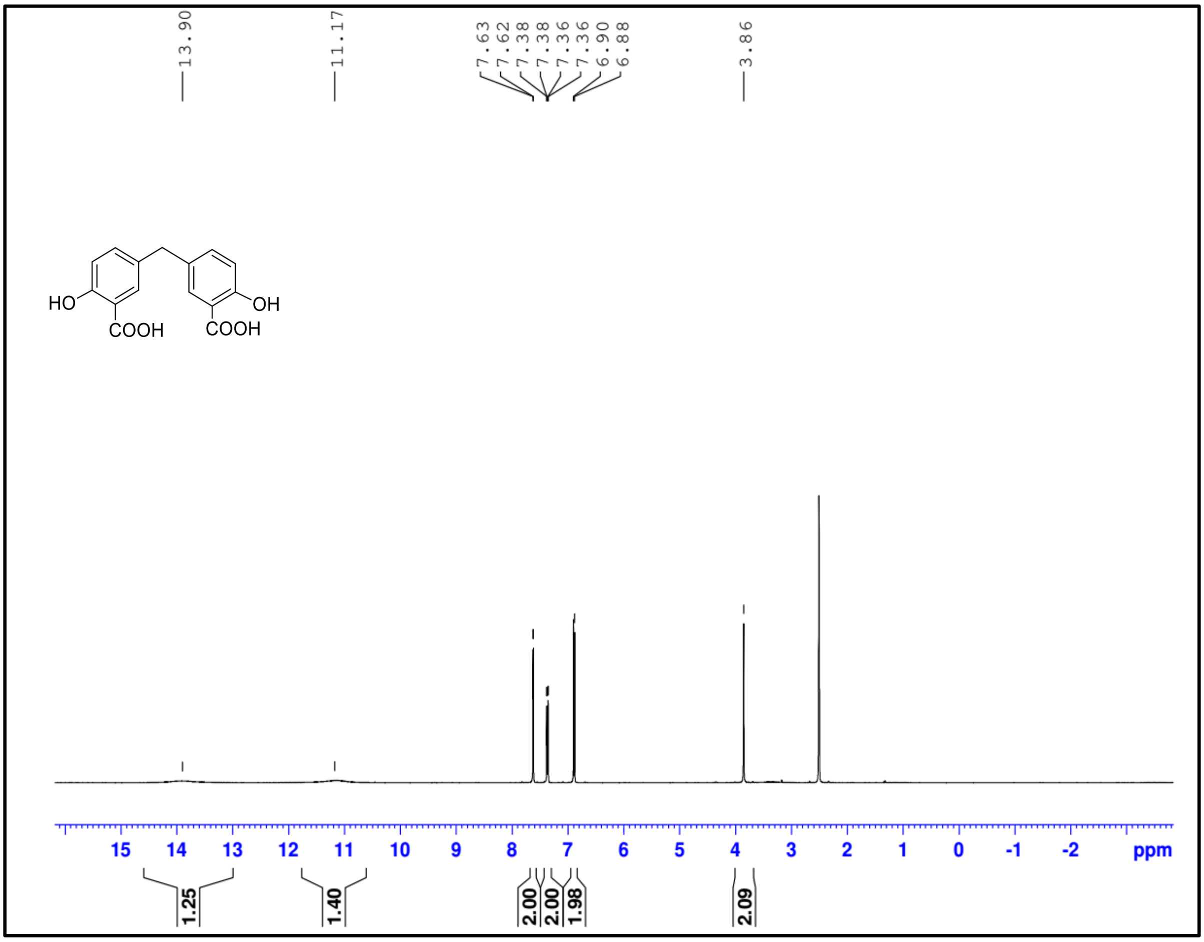

Figure S5: ^{13}C NMR spectrum of compound **3**

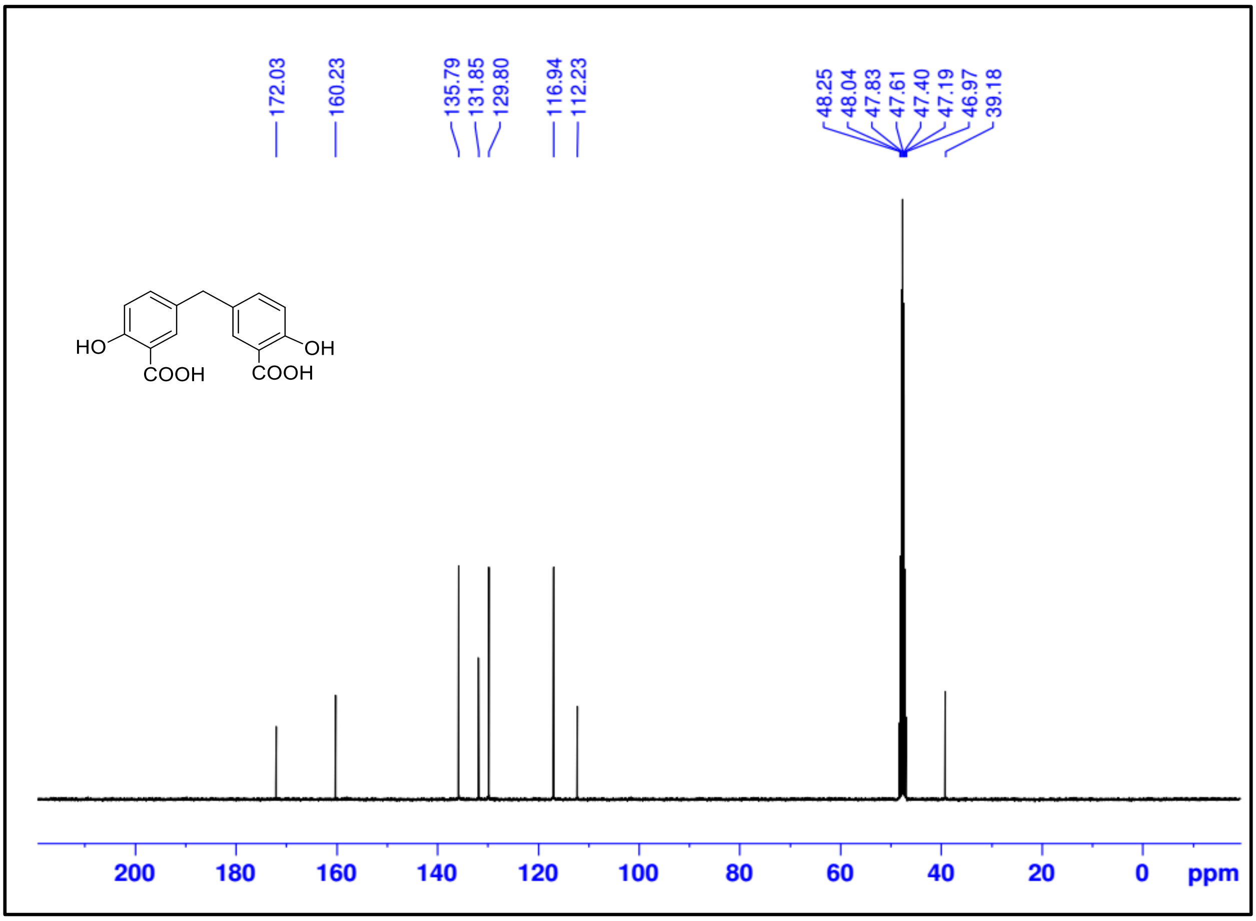

Figure S6: HRMS spectrum of compound **3** showing $[M-H]^-$ peak.

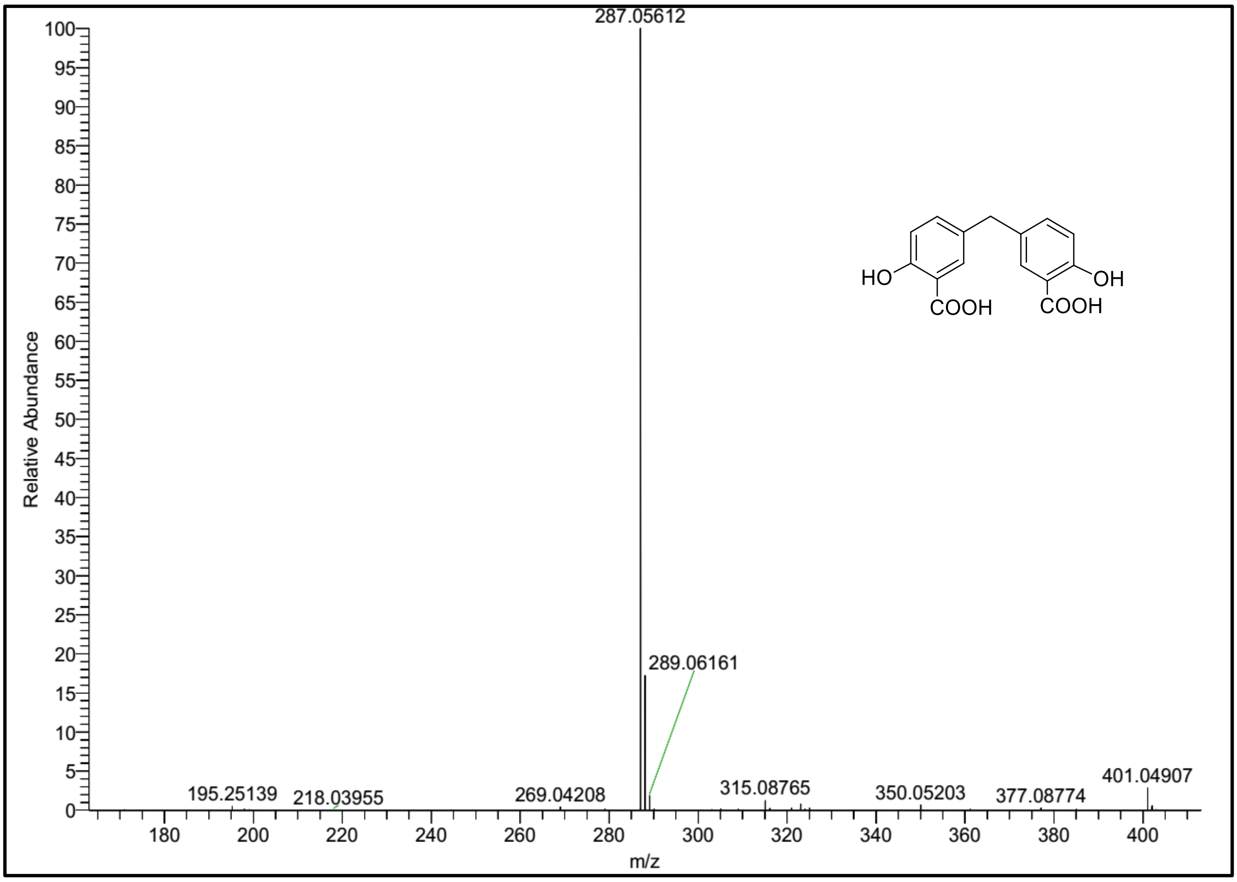

Figure S7: FT-IR spectrum of compound **4**

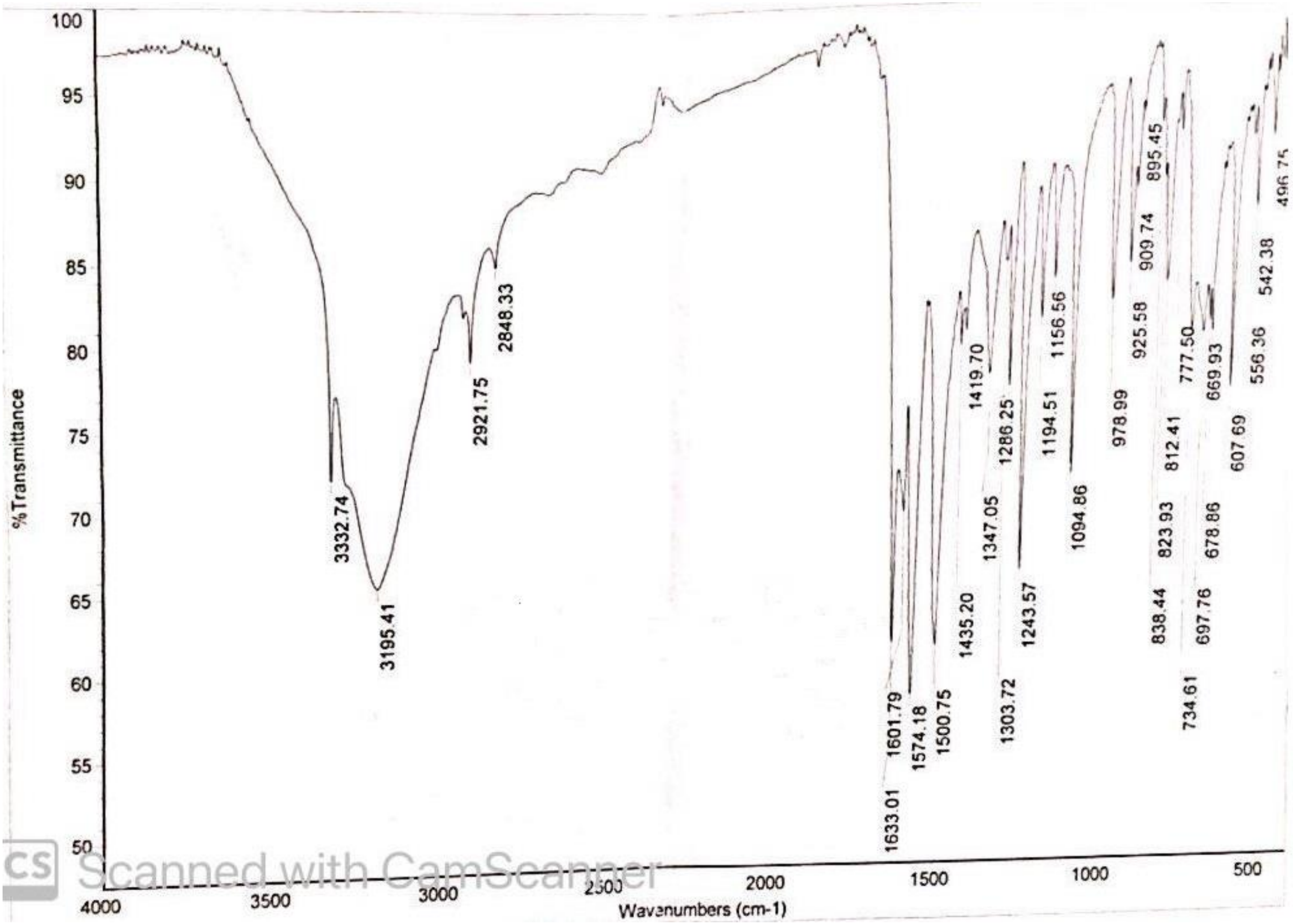

CS Scanned with CamScanner

Figure S8: ^{1}H NMR spectrum (400 MHz, DMSO-d_6) of compound **4**

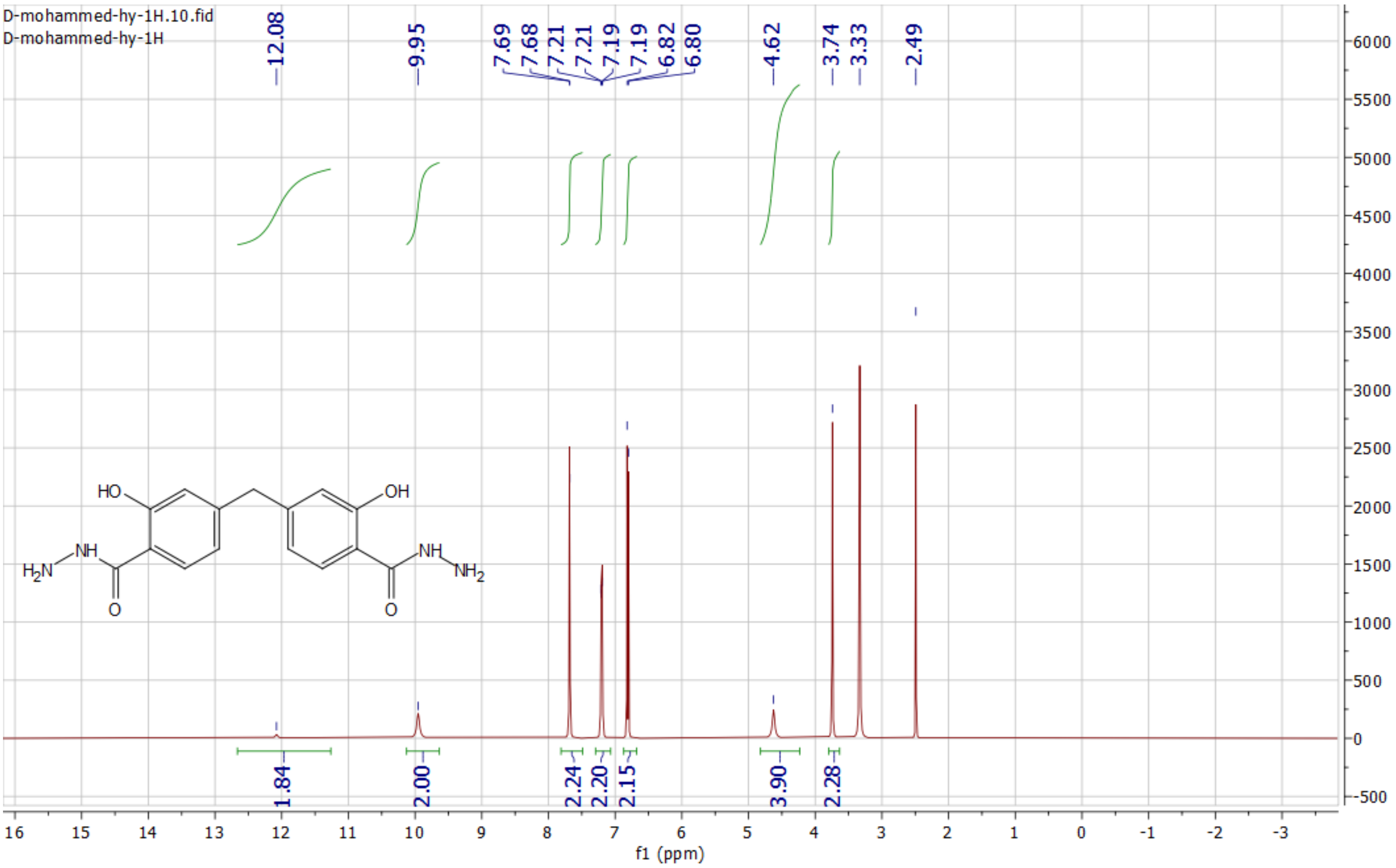

^{1}H NMR (400 MHz, δ ppm DMSO-d_6): 12.08 (s, 2H, phenolic-OH), 9.95 (s, 2H, amidic-NH), 7.68 (d, J= 2 Hz, 2H, Ar–H), 7.19 (two d, J= 8.4 Hz, 2 Hz, 2H, Ar–H), 6.81 (d, J= 8.4 Hz, 2H, Ar–H), 4.62 (s, 4H, C=O-NH-<u>NH$_2$</u>), 3.74 (s, 2H, Ar–CH$_2$).

Figure S9: ^{1}H NMR spectrum (400 MHz, DMSO-d_6) of compound **5a**

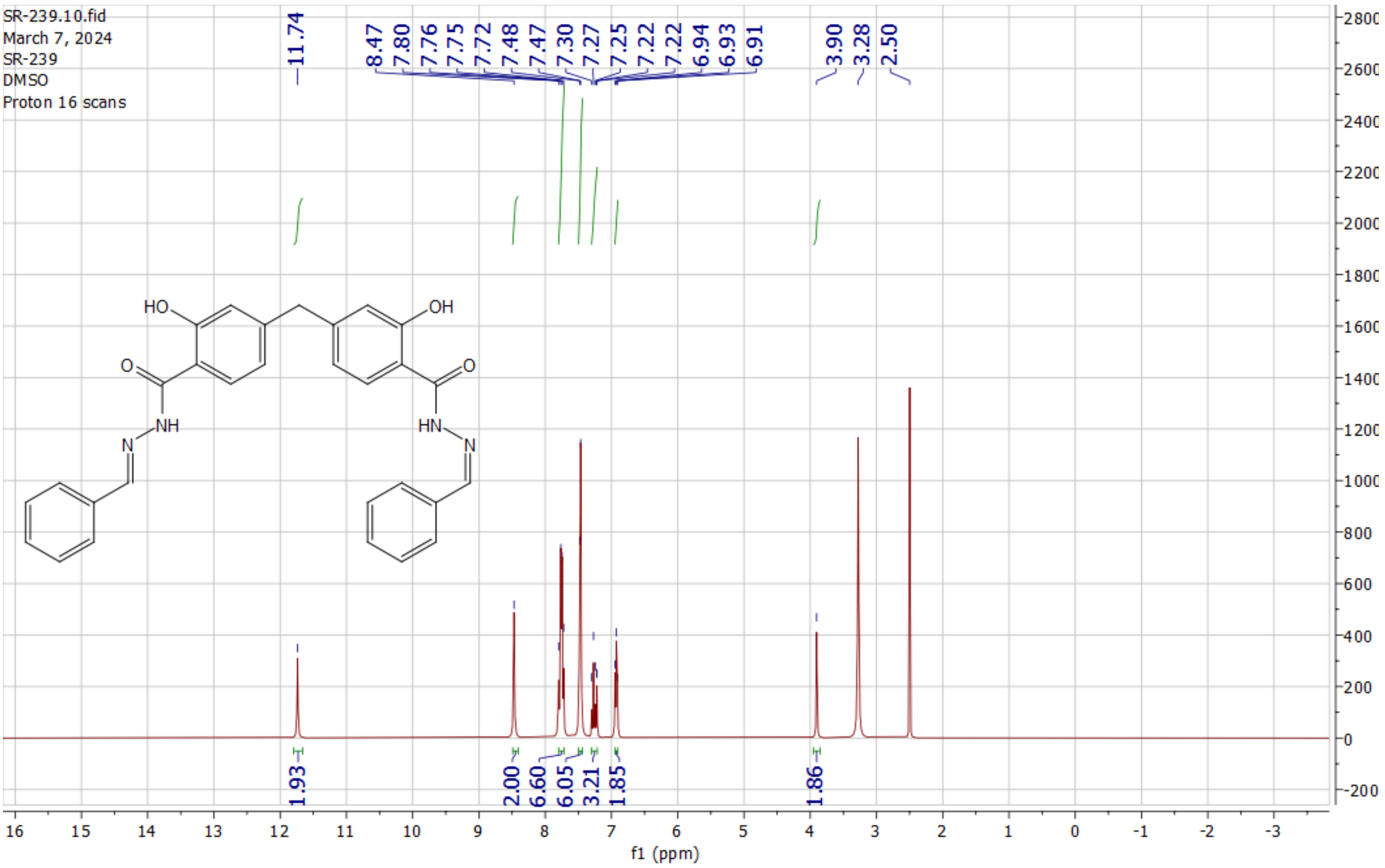

^{1}H NMR (400 MHz, δ ppm DMSO-d_6): 11.74 (s, 2H, phenolic-OH), 8.47 (s, 2H, amidic-NH), 7.82-7.62 (m, 5H, Ar–H, 2H, CH=N), 7.47 (d, J = 6.1 Hz, 6H, Ar–H), 7.32-7.17 (m, 3H, Ar–H), 6.93 (t, J = 7.8 Hz, 2H, Ar–H), 3.90 (s, 2H, Ar–CH_2).

Figure S10: LC-MS mass spectrum of compound **5a**

Spectrum Plot Report

Name	SR-239	Rack Pos.		Instrument	APOGEE-LCMS	Operator	SYSTEM (SYSTEM)
Inj. Vol. (ul)	3	Plate Pos.		IRM Status			
Data File	20240408-Fast Scan-95 Org-SR-239a.d	Method (Acq)	20240404-Fast Scan-95 Org.m	Comment	DMSO-Heat-ACN-Filter-Dilute 50x in ACN	Acq. Time (Local)	2024-04-09 1:23:41 PM (UTC-07:00)

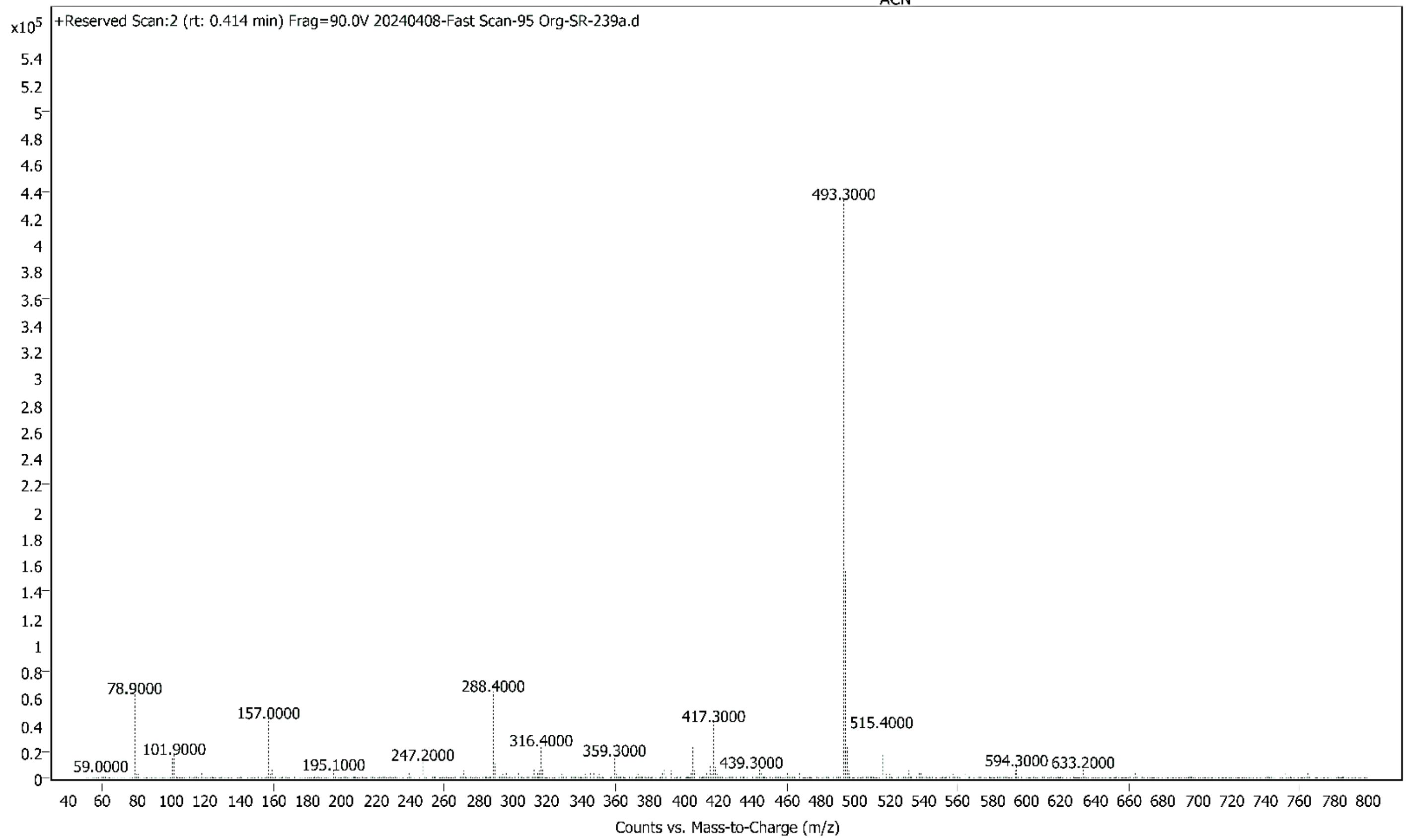

Figure S11: ^{1}H NMR spectrum (400 MHz, DMSO-d_6) of compound **5b**

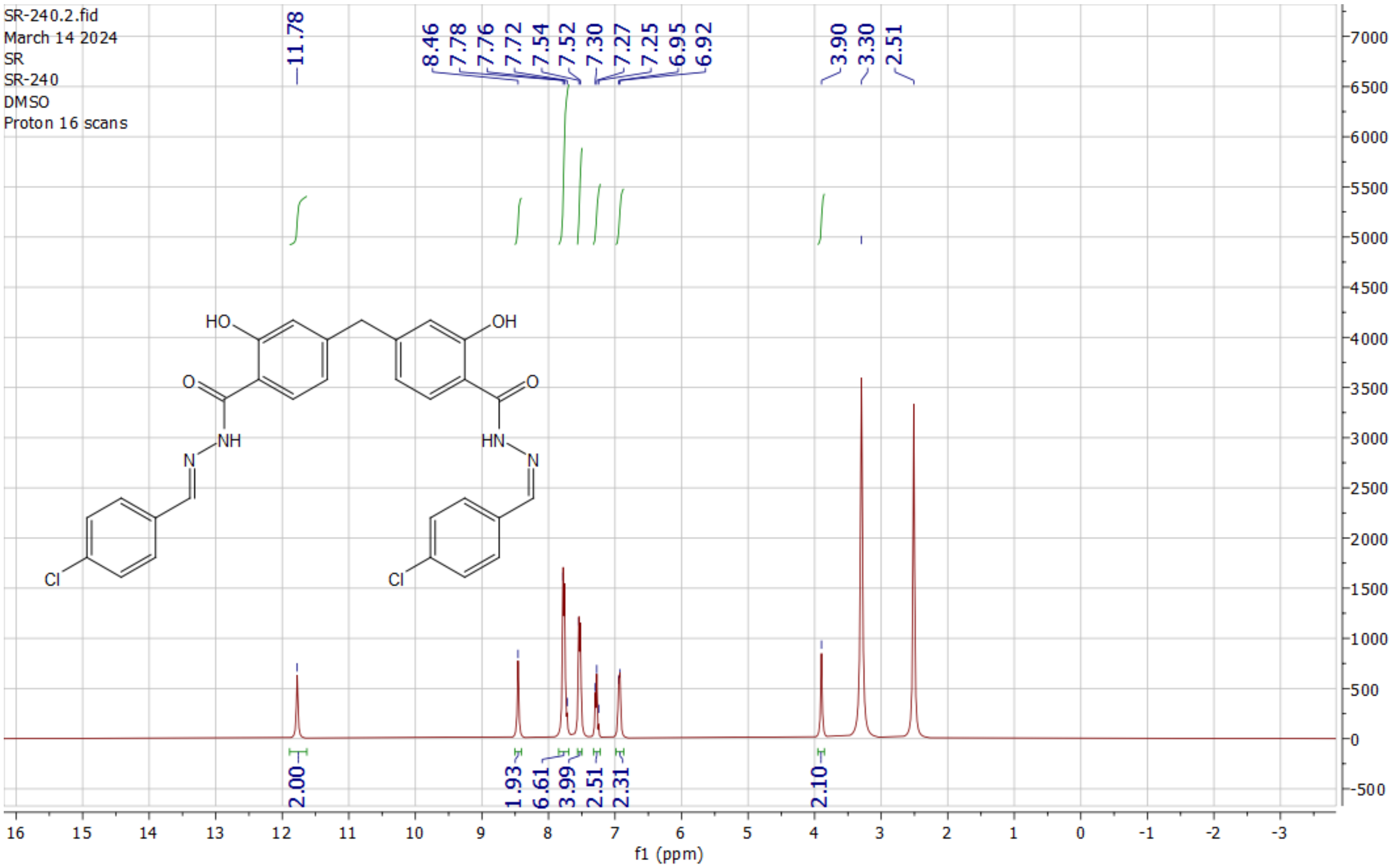

^{1}H NMR (400 MHz, δ ppm DMSO-d_6): 11.78 (s, 2H, phenolic-OH), 8.46 (s, 2H, amidic-NH), 7.78-7.72 (m, 2H, CH=N, 5H, Ar–H,), 7.53 (d, J = 5.2 Hz, 4H, Ar–H), 7.28 (d, J = 7.3 Hz, 3H, Ar–H), 6.93 (d, J = 5.7 Hz, 2H, Ar–H), 3.90 (s, 2H, Ar–CH_2).

Figure S12: LC-MS spectrum of compound **5b**

Spectrum Plot Report

Name	SR-240	Rack Pos.		Instrument	APOGEE-LCMS	Operator	SYSTEM (SYSTEM)
Inj. Vol. (ul)	3	Plate Pos.		IRM Status			
Data File	20240408-Fast Scan-95 Org-SR-240a.d	Method (Acq)	20240404-Fast Scan-95 Org.m	Comment	DMSO-Heat-ACN-Filter-Dilute 50x in ACN	Acq. Time (Local)	2024-04-08 3:41:40 PM (UTC-07:00)

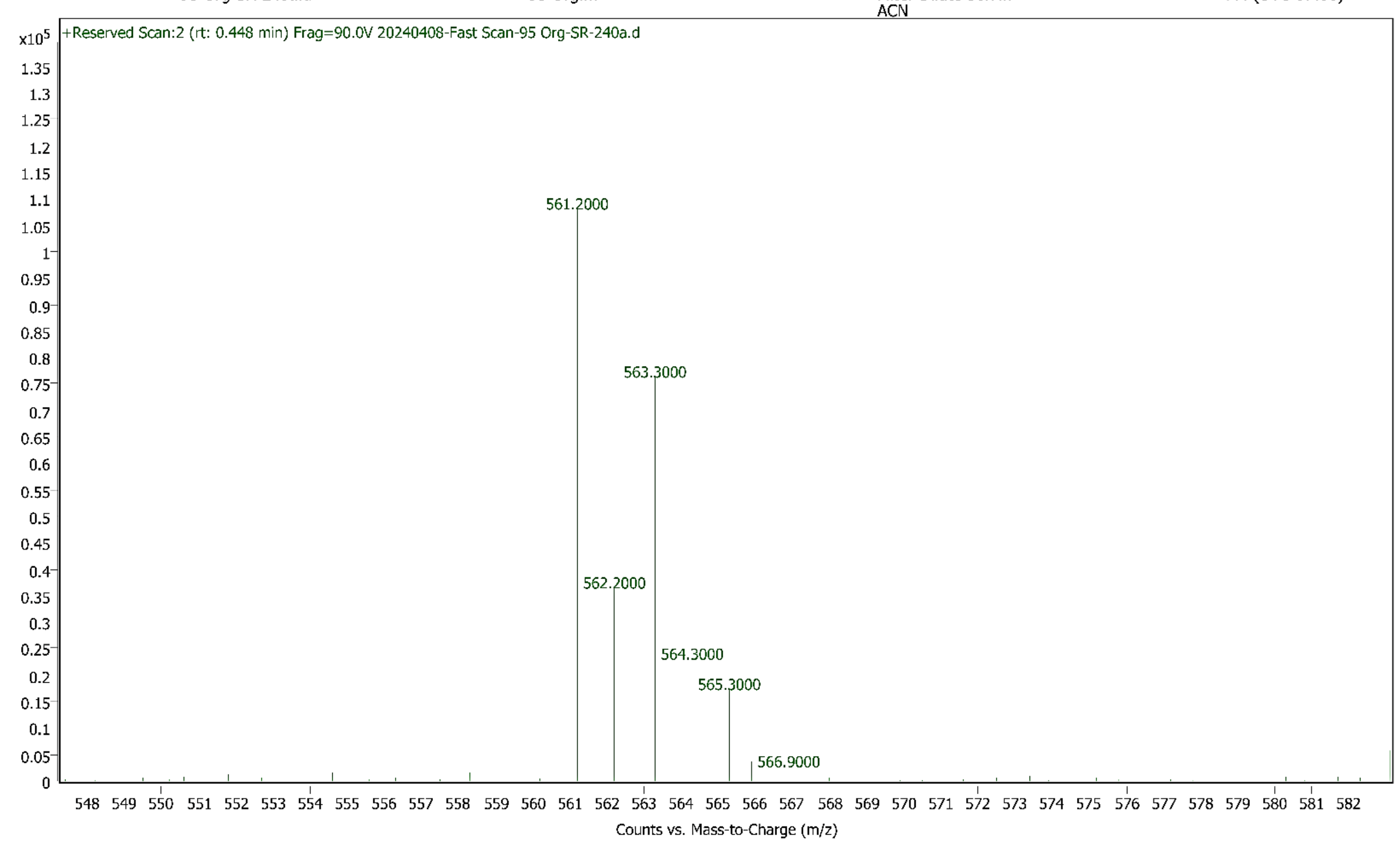

Figure S13: ^{1}H NMR spectrum (400 MHz, DMSO-d_6) of compound **5c**

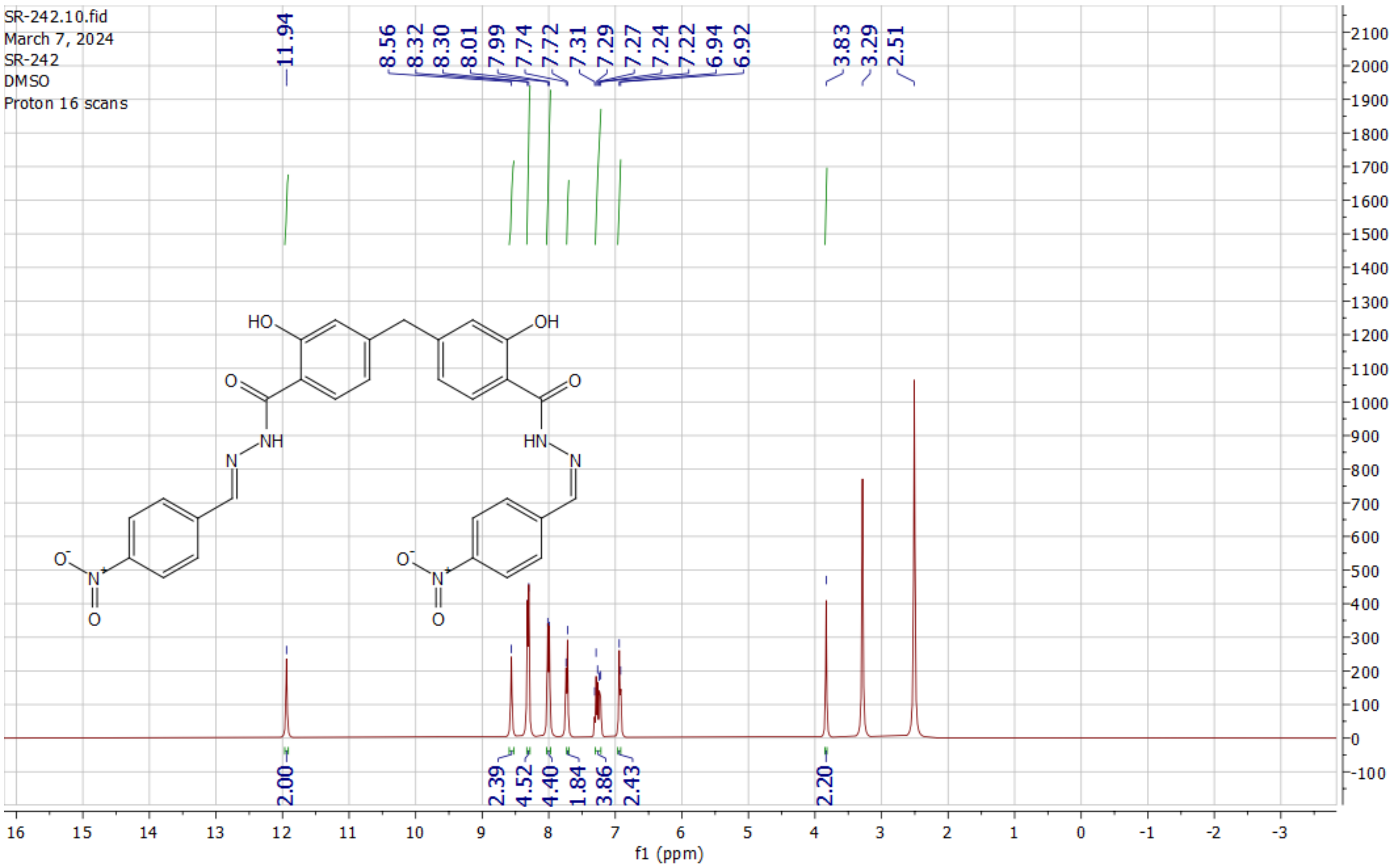

^{1}H NMR (400 MHz, δ ppm DMSO-d_6): 11.94 (s, 2H,phenolic-OH), 8.56 (s, 2H, amidic-NH), 8.30 (d, J = 7.3 Hz, 4H, Ar–H), 8.00 (d, J = 5.1 Hz, 4H, Ar–H), 7.72 (d, J = 9 Hz, 2H, Ar–H), 7.31-7.22 (m, 2H, CH=N, 2H, Ar–H), 6.93 (d, J = 7.5 Hz, 2H, Ar–H), 3.83 (s, 2H, Ar–CH_2)

Figure S14: LC-MS spectrum of compound **5c**

Spectrum Plot Report

Name	SR-242	Rack Pos.		Instrument	APOGEE-LCMS	Operator	SYSTEM (SYSTEM)
Inj. Vol. (ul)	3	Plate Pos.		IRM Status			
Data File	20240409-Fast Scan-95 Org-SR-242a.d	Method (Acq)	20240404-Fast Scan-95 Org.m	Comment	DMSO-Heat-ACN-Filter-Dilute 50x in ACN	Acq. Time (Local)	2024-04-09 2:34:04 PM (UTC-07:00)

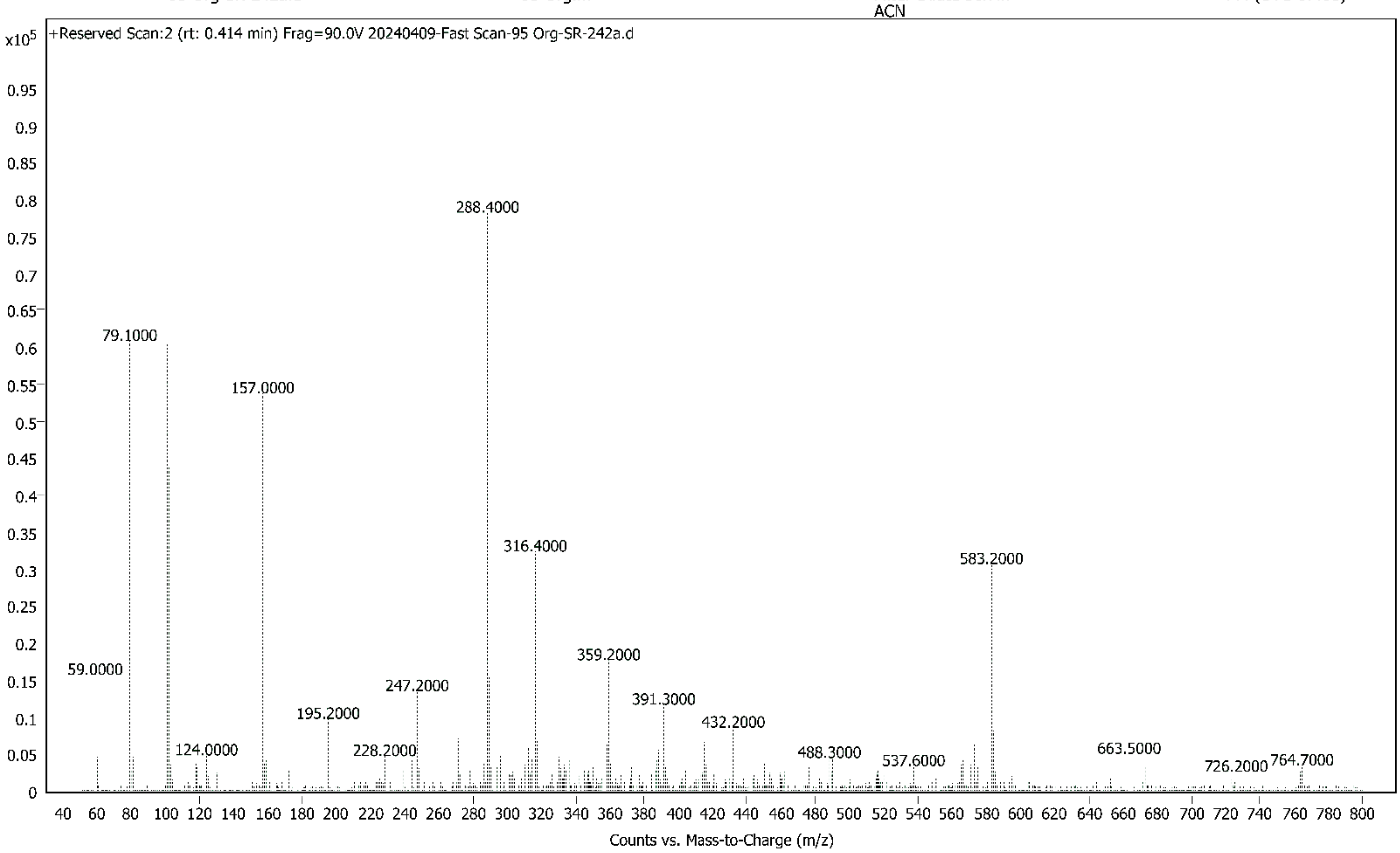

Figure S15: ^{1}H NMR spectrum (400 MHz, DMSO-d_6) of compound **5d**

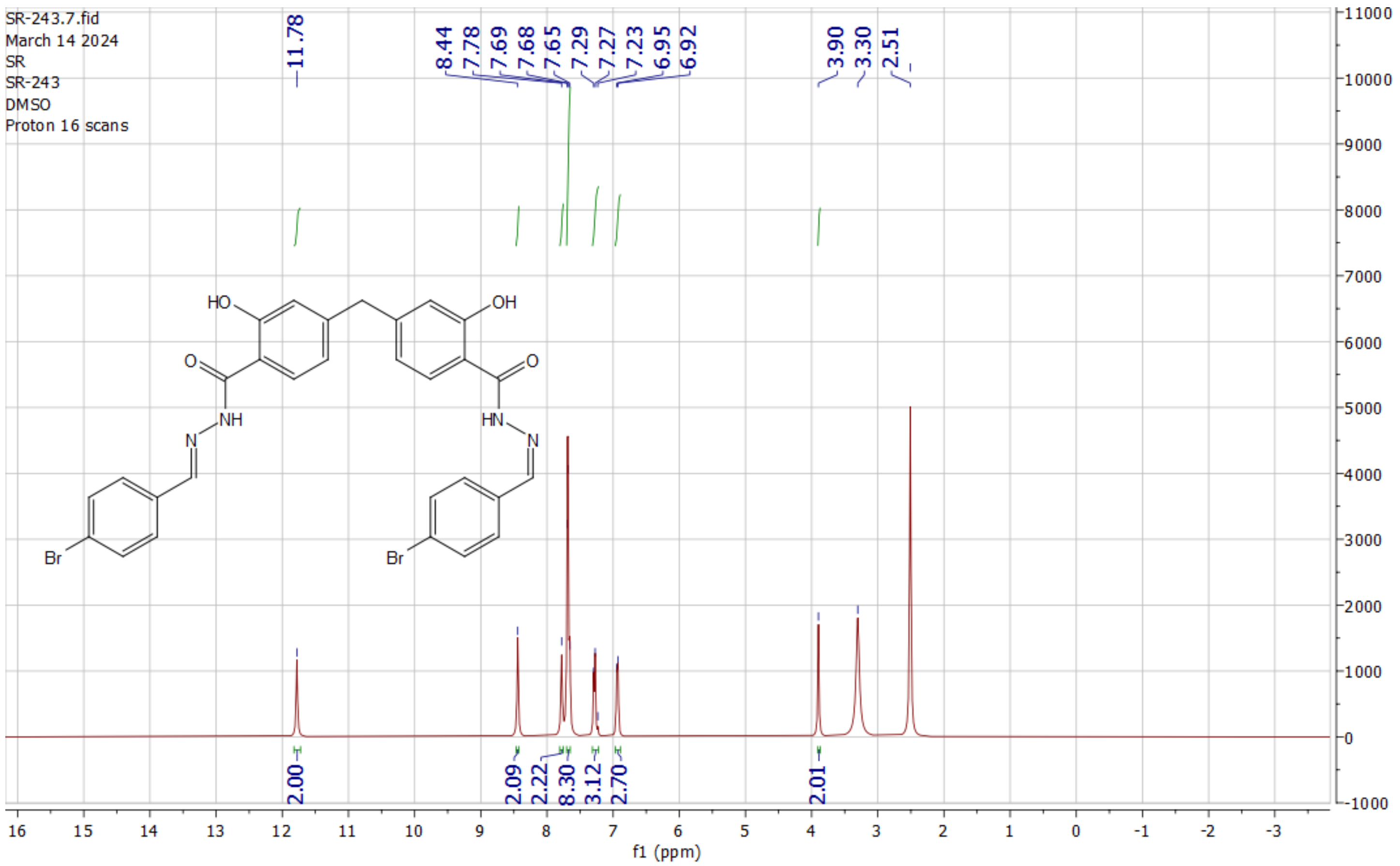

^{1}H NMR (400 MHz, δ ppm DMSO-d_6): 11.78 (s, 2H, phenloic-OH), 8.44 (s, 2H, amidic-NH), 7.78 (s, 2H, CH=N), 7.72-7.60 (m, 8H, Ar–H), 7.28 (d, J= 7.8 Hz, 3H, Ar–H), 6.93 (d, J= 6.7 Hz, 3H, Ar–H), 3.90 (s, 2H, Ar–CH$_2$).

Figure S16: LC-MS spectrum of compound **5d**

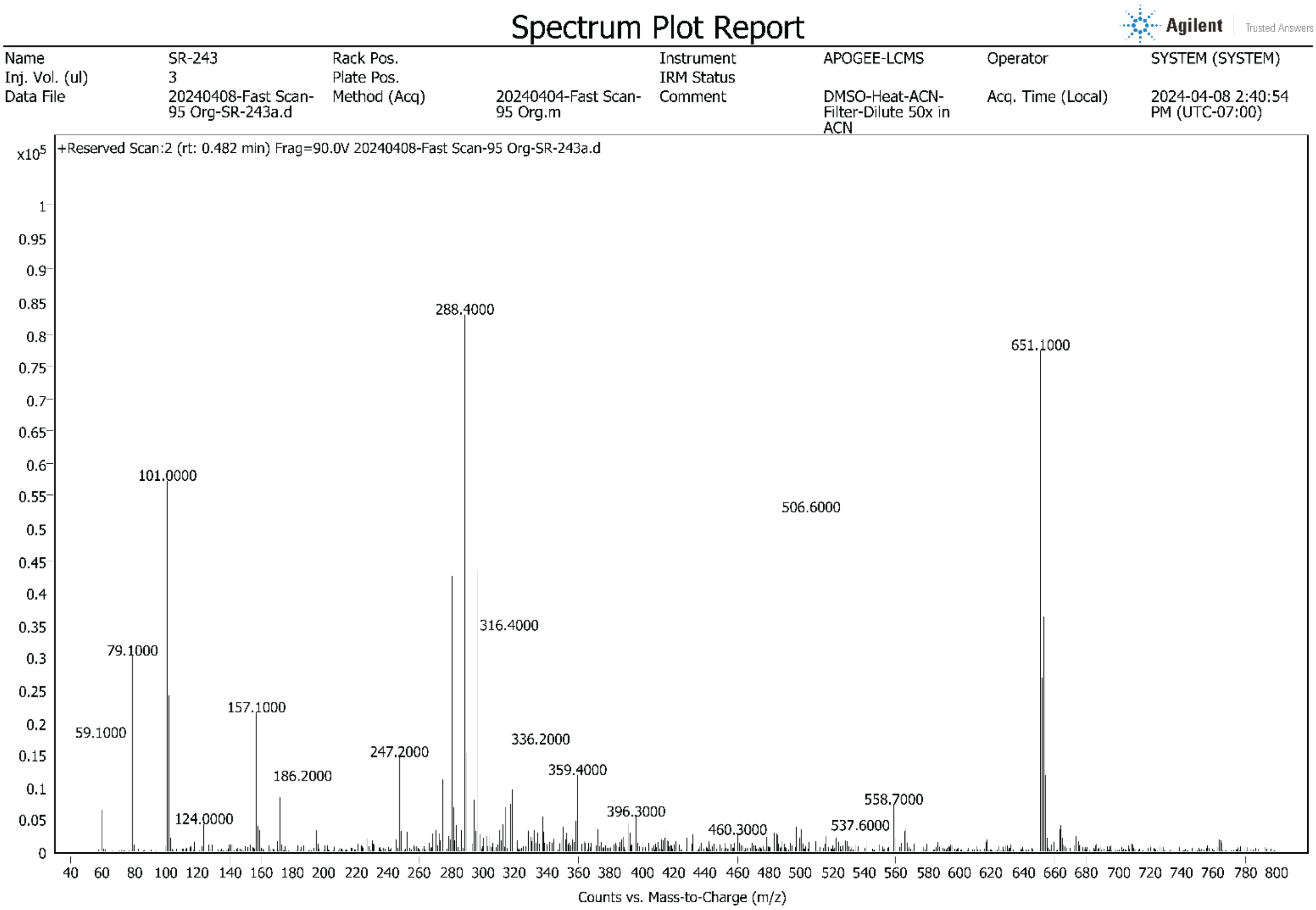

Figure S17: ^{1}H NMR spectrum (400 MHz, DMSO-d_6) of compound **5e**

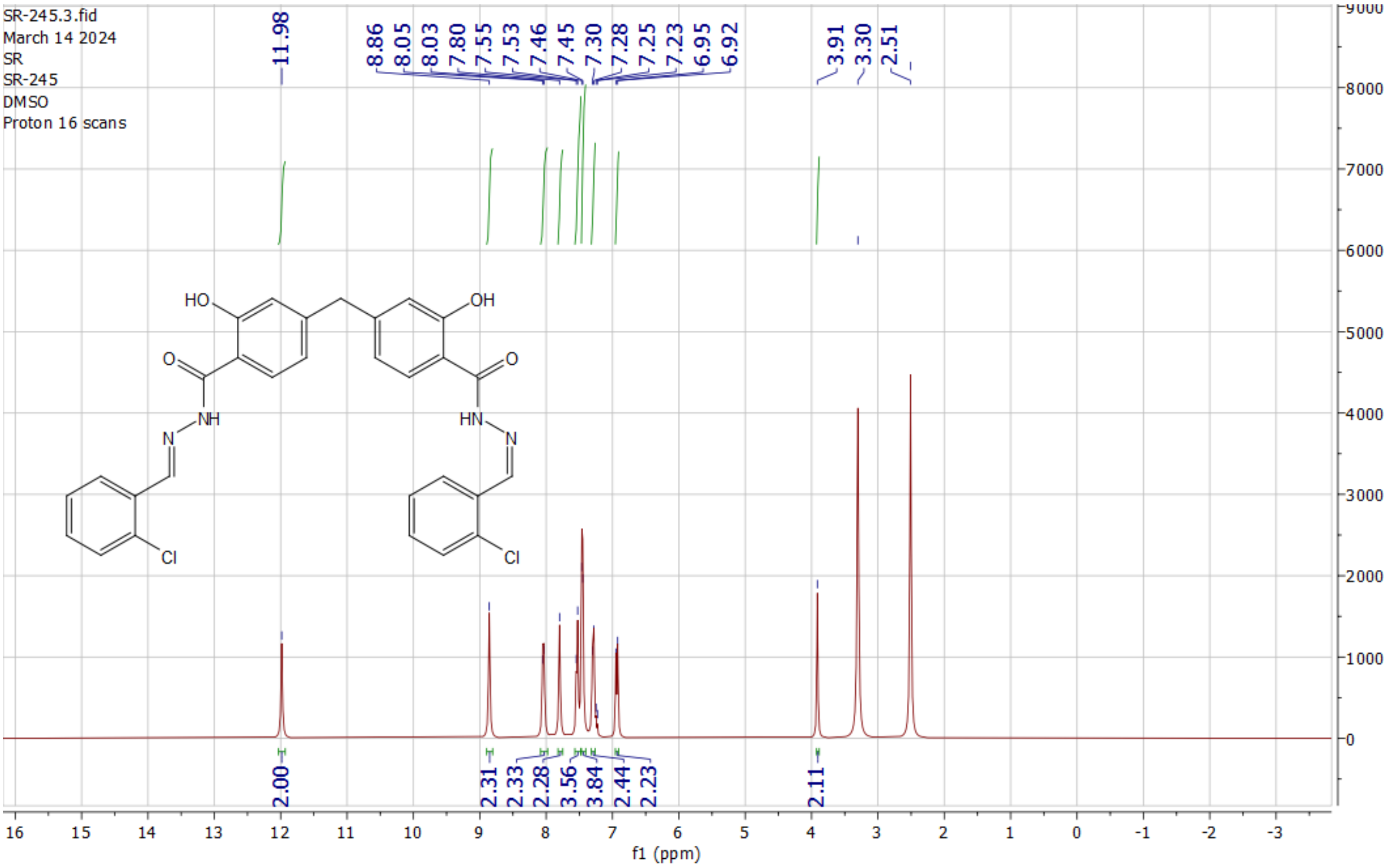

^{1}H NMR (400 MHz, δ ppm DMSO-d_6): 11.98 (s, 2H, phenolic-OH), 8.86 (s, 2H, amidic-NH), 8.04 (d, J= 6.5 Hz, 2H, Ar–H), 7.80 (s, 2H, CH=N), 7.53 (d, J= 7.5 Hz, 4H, Ar–H), 7.46 (d, J= 6 Hz, 4H, Ar–H), 7.30-7.23 (m, 2H, Ar–H), 7.28 (d, J= 7 Hz, 2H, Ar–H), 3.91 (s, 2H, Ar–CH_2).

Figure S18: LC-MS spectrum of compound **5e**

Spectrum Plot Report

Agilent | Trusted Answers

Name	SR-245	Rack Pos.		Instrument	APOGEE-LCMS	Operator	SYSTEM (SYSTEM)
Inj. Vol. (ul)	3	Plate Pos.		IRM Status			
Data File	20240408-Fast Scan-95 Org-SR-245a.d	Method (Acq)	20240404-Fast Scan-95 Org.m	Comment	DMSO-Heat-ACN-Filter-Dilute 100x in ACN	Acq. Time (Local)	2024-04-08 2:07:51 PM (UTC-07:00)

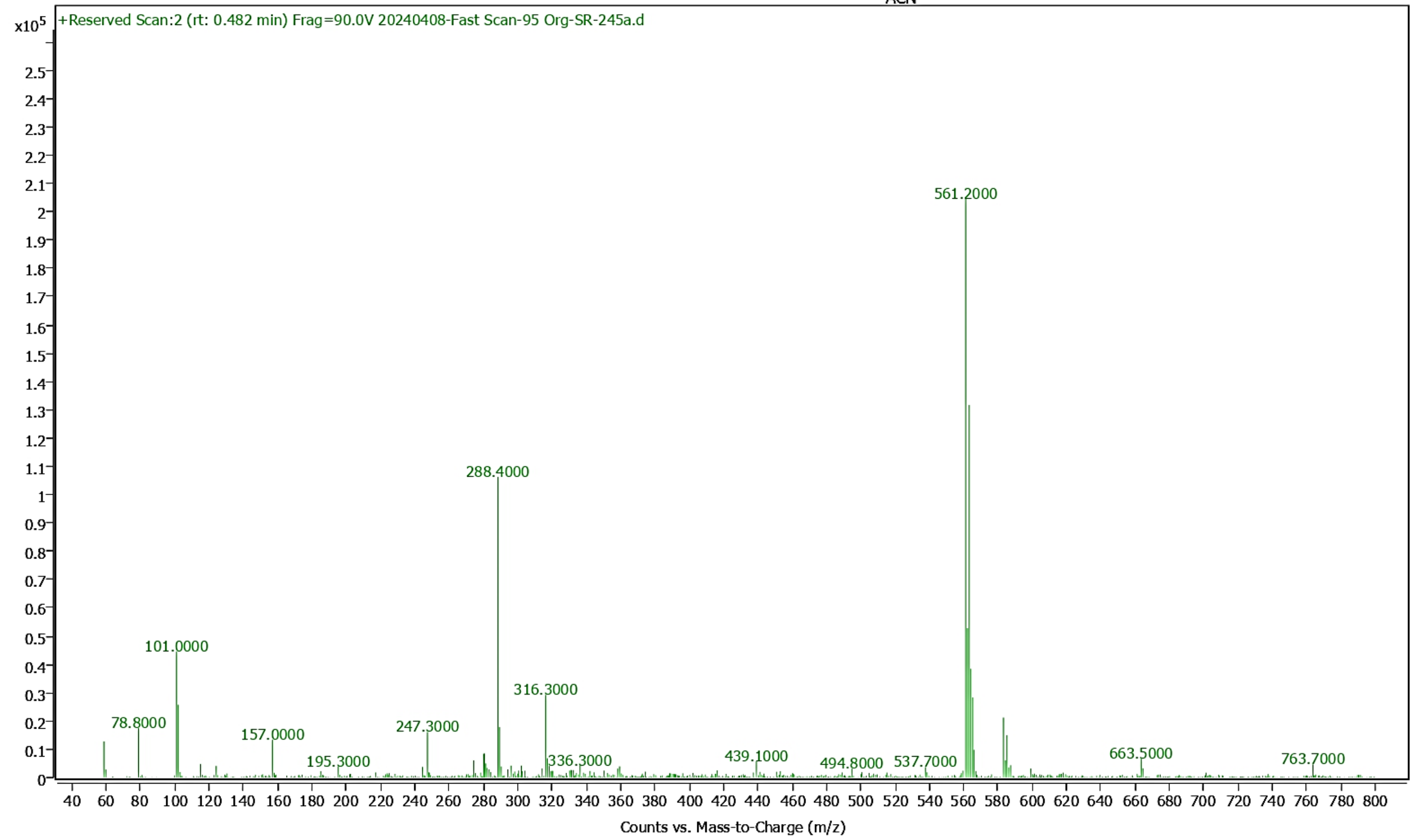

Figure S19: ^{1}H NMR spectrum (400 MHz, DMSO-d_6) of compound **5f**

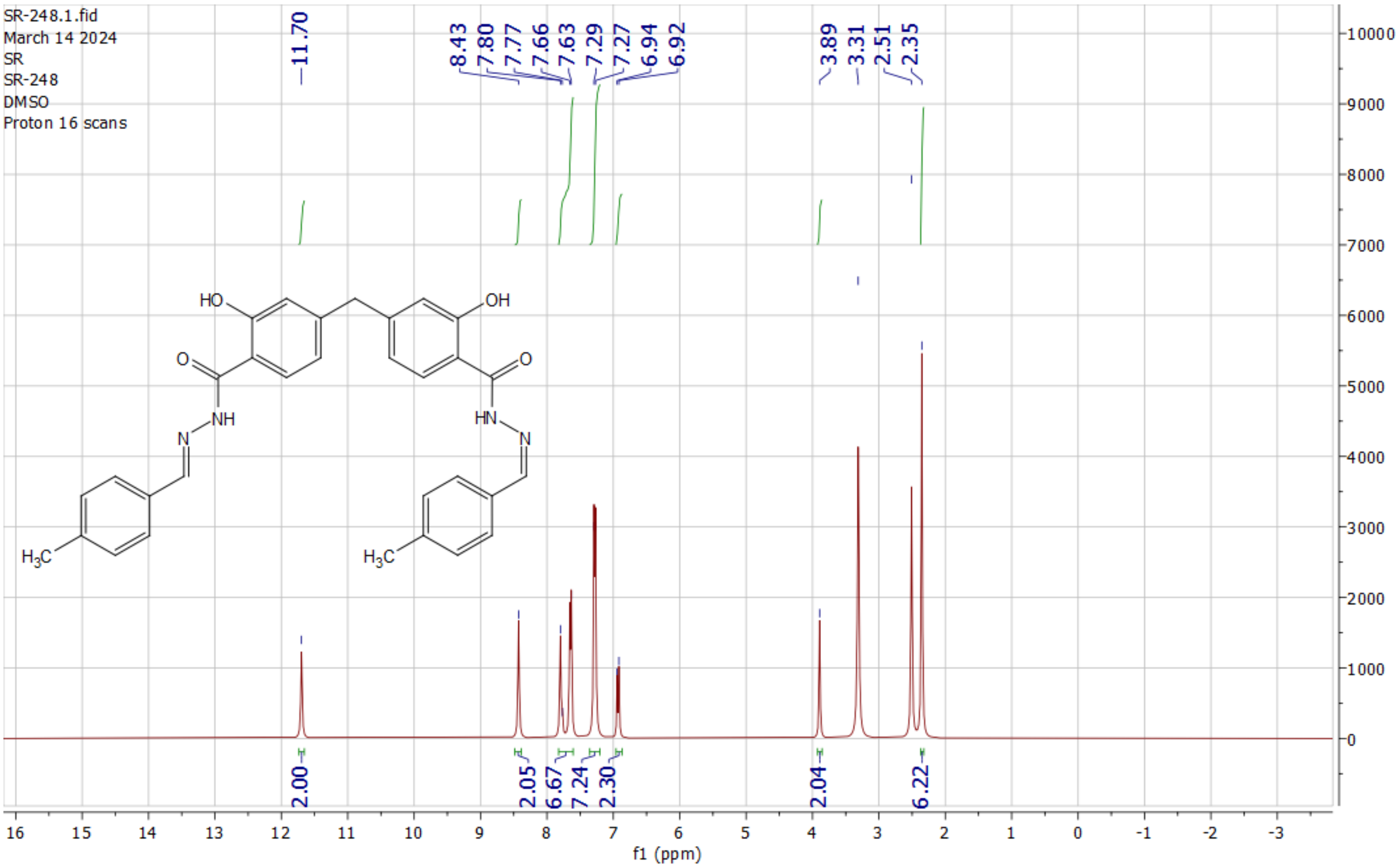

^{1}H NMR (400 MHz, δ ppm DMSO-d_6): 11.70 (s, 2H, phenolic-OH), 8.43 (s, 2H, amidic-NH), 7.80 (s, 2H, CH=N), 7.75-7.59 (m, 5H, Ar–H), 7.28 (d, J = 7 Hz, 7H, Ar–H), 6.92 (d, J = 7.2 Hz, 2H, Ar–H), 3.89 (s, 2H, Ar–CH_2), 2.85 (s, 6H, Ar–CH_3).

Figure S20: LC-MS spectrum of compound **5f**

Spectrum Plot Report

Name	SR-248	Rack Pos.		Instrument	APOGEE-LCMS	Operator	SYSTEM (SYSTEM)
Inj. Vol. (ul)	3	Plate Pos.		IRM Status			
Data File	20240404-Fast Scan-95 Org-SR-248a.d	Method (Acq)	20240404-Fast Scan-95 Org.m	Comment	DMSO-Heat-ACN-Filter-Dilute 300x in ACN	Acq. Time (Local)	2024-04-04 2:31:01 PM (UTC-07:00)

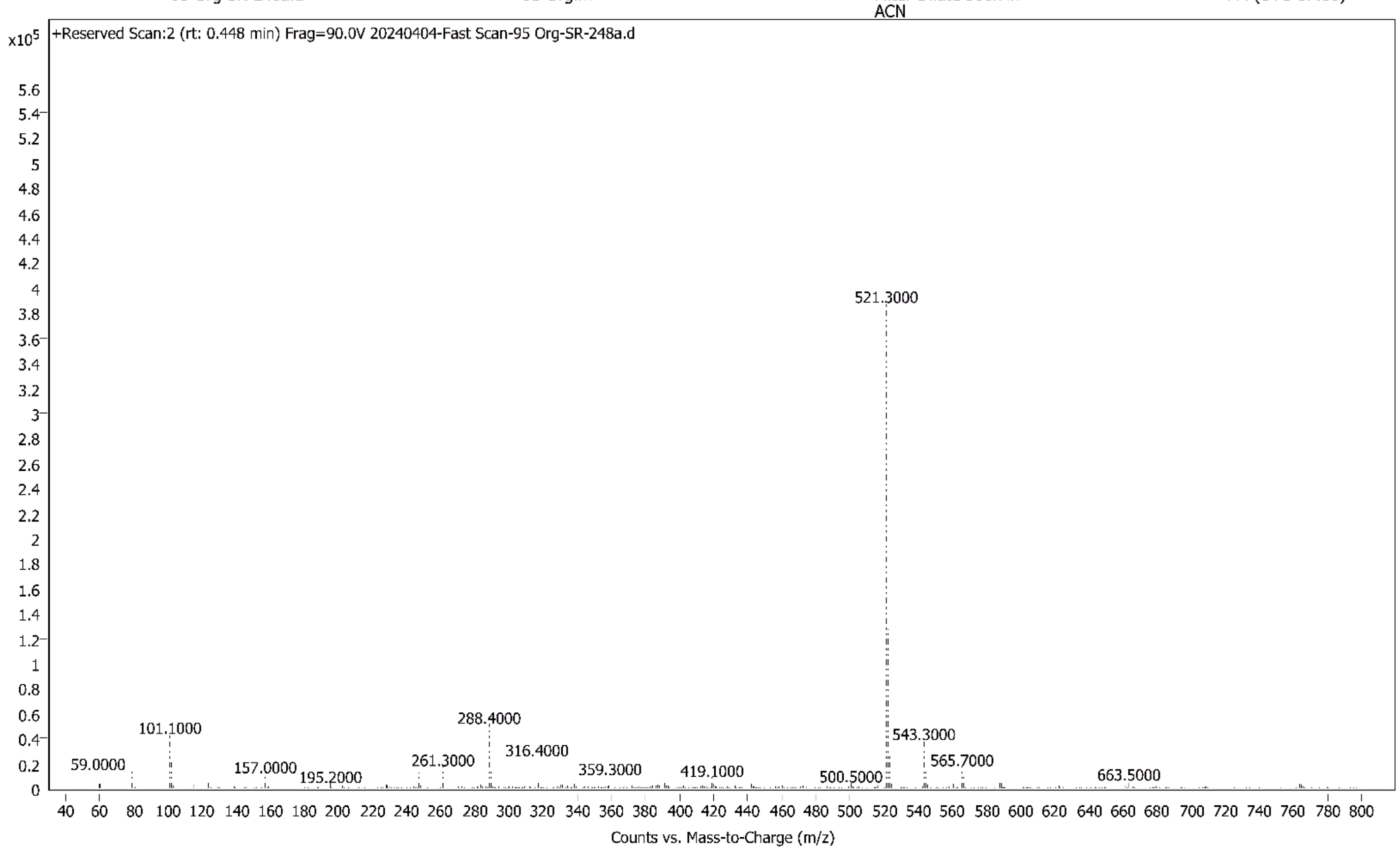

Figure S21: ^{1}H NMR spectrum (400 MHz, DMSO-d_6) of compound **5g**

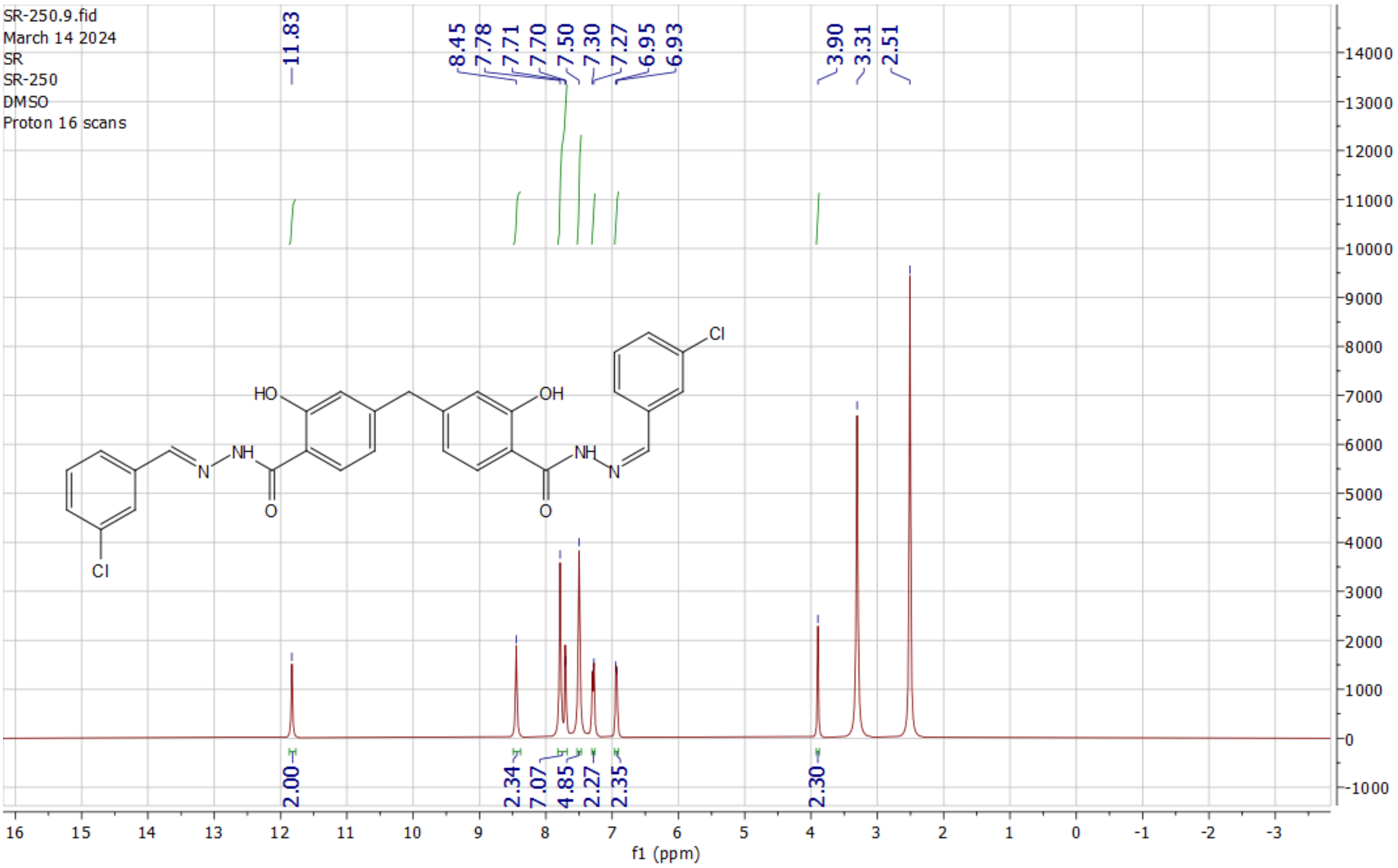

^{1}H NMR (400 MHz, δ ppm DMSO-d_6): 11.83 (s, 2H, phenolic-OH), 8.45 (s, 2H, amidic-NH), 7.81-7.67 (m, 2H, CH=N, 5H, Ar-H), 7.55-7.45 (m, 5H, Ar-H), 7.28 (d, J = 7.7 Hz, 2H, Ar–H), 6.93 (d, J = 6.7 Hz, 2H, Ar–H), 3.91 (s, 2H, Ar–CH_2).

Figure S22: LC-MS spectrum of compound **5g**

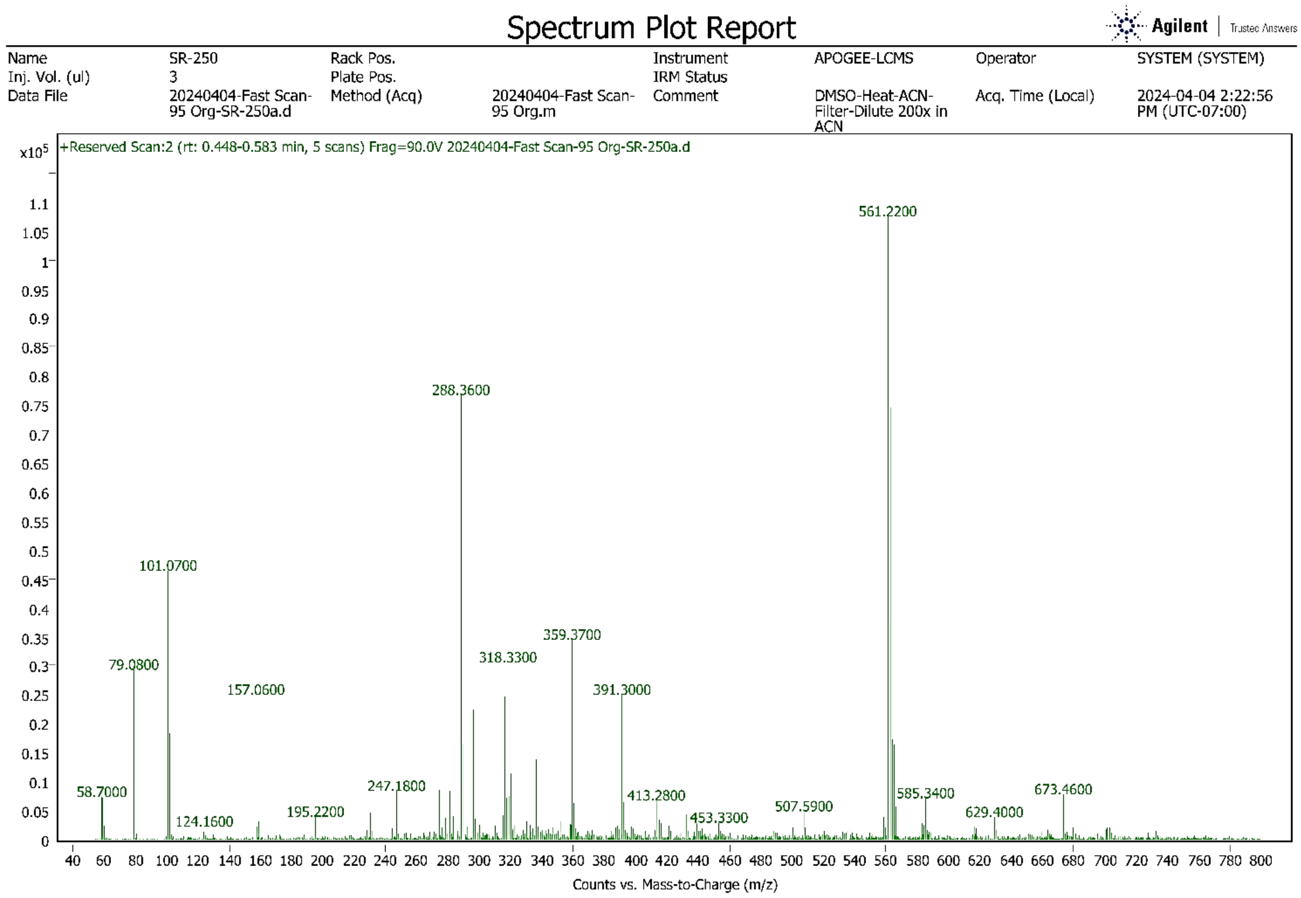

Figure S23: ^{1}H NMR spectrum (400 MHz, DMSO-d_6) of compound **5h**

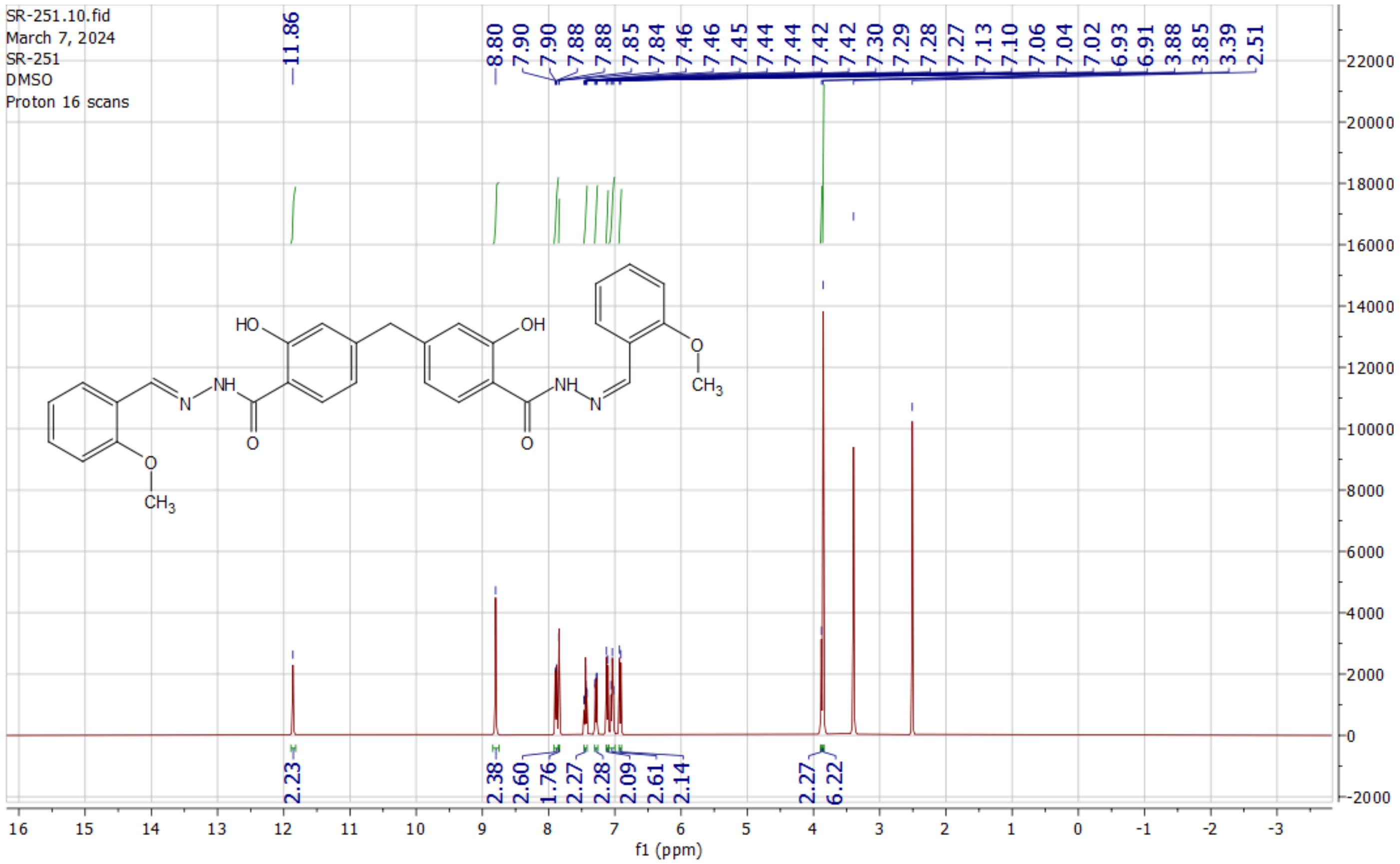

^{1}H NMR (400 MHz, δ ppm DMSO-d_6): 11.86 (s, 2H, phenolic-OH), 8.80 (s, 2H, amidic-NH), 7.88 (two d, J = 6.4 Hz, 1.3 Hz, 3H, Ar-H), 7.84 (s, 2H, CH=N), 7.46-7.42 (m, 2H, Ar–H), 7.28 (two d, J = 6.7 Hz, 1.8 Hz, 2H, Ar-H), 7.11 (d, J = 8.3 Hz, 2H, Ar–H), 7.03 (t, J = 7.5 Hz, 3H, Ar–H), 6.91 (d, J = 8.4 Hz, 2H, Ar–H), 3.88 (s, 2H, Ar–CH_2), 3.85 (s, 6H, Ar–OCH_3).

Figure S24: LC-MS spectrum of compound **5h**

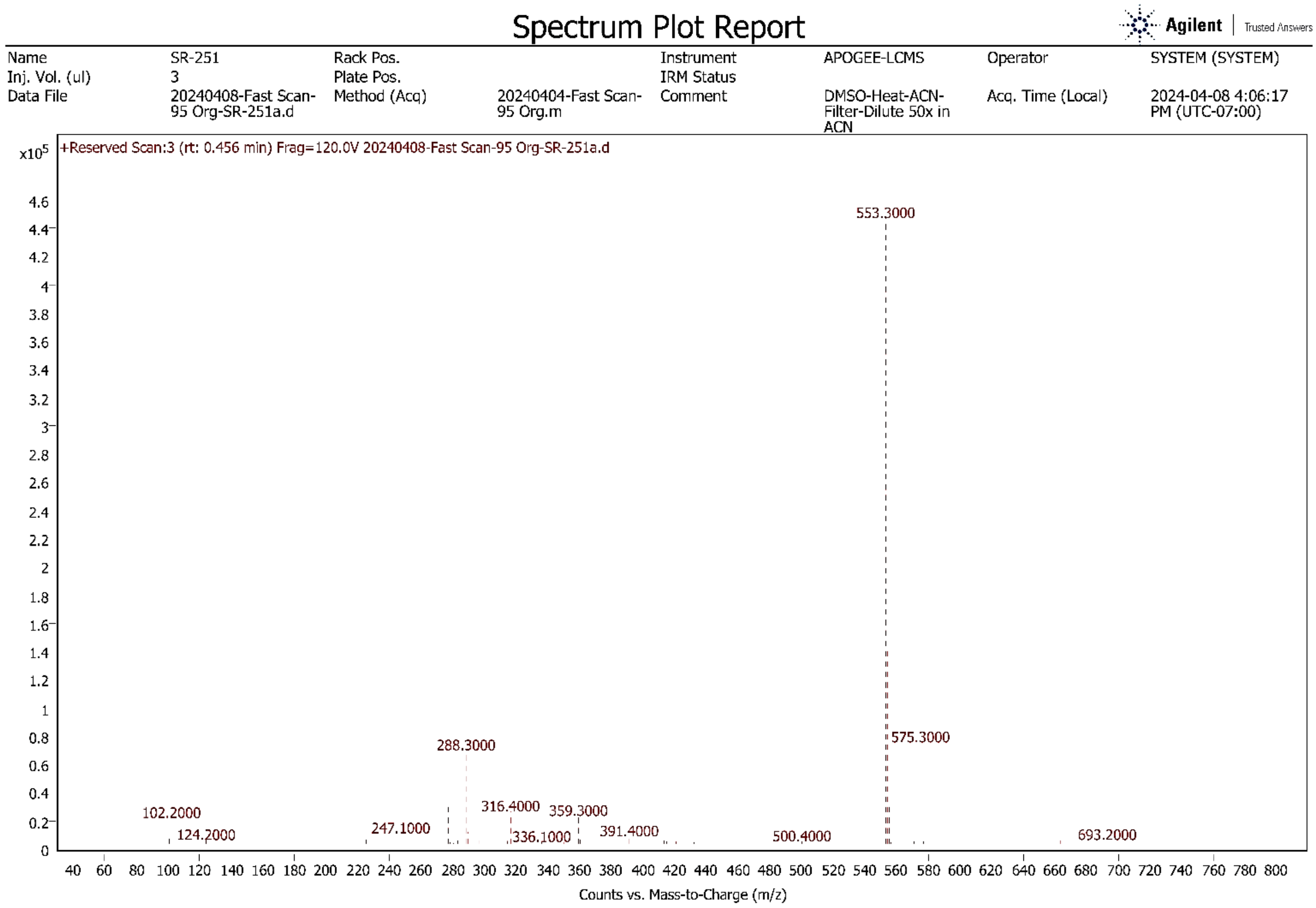

Figure S25: ^{1}H NMR spectrum (400 MHz, DMSO-d_6) of compound **5i**

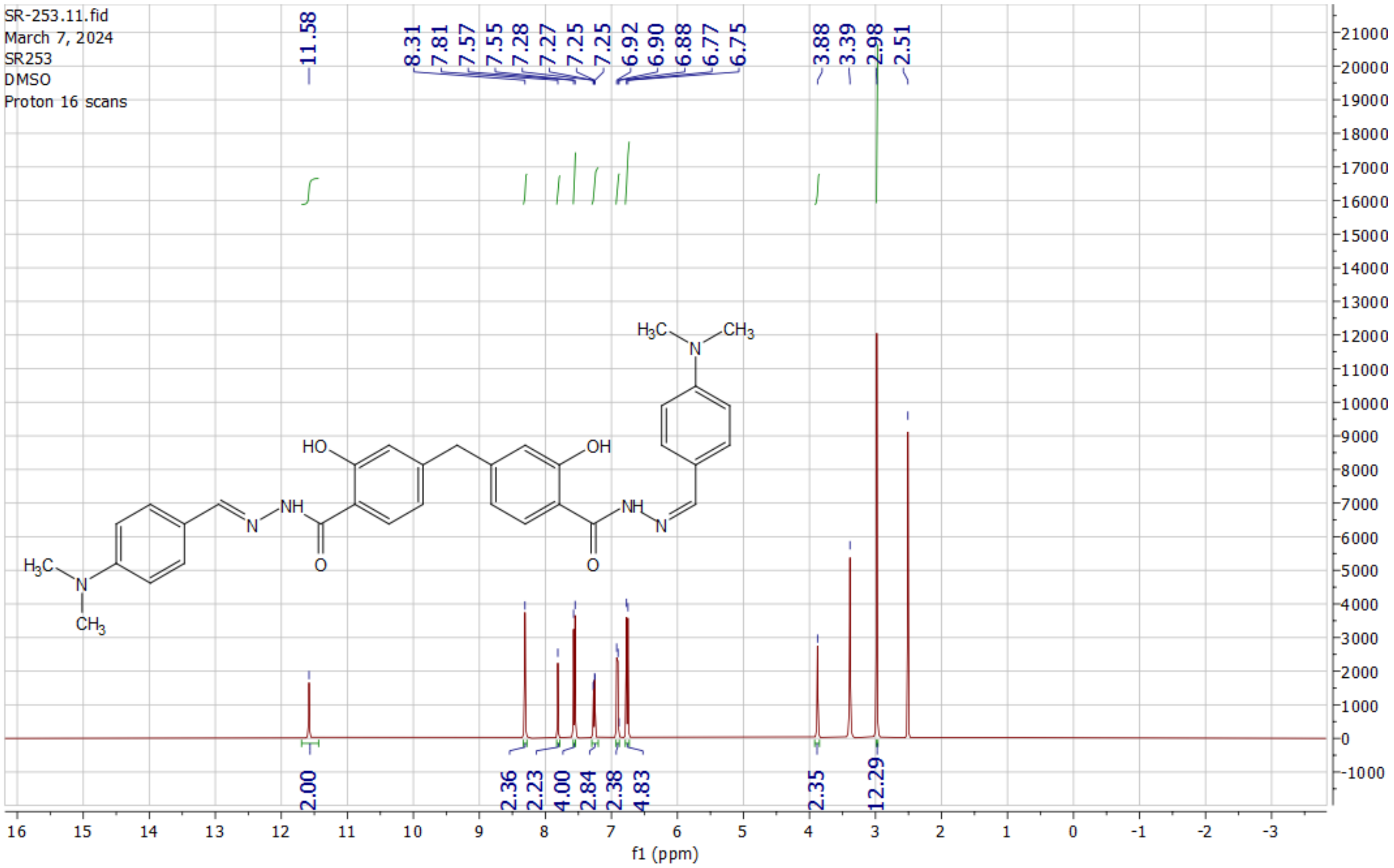

^{1}H NMR (400 MHz, δ ppm DMSO-d_6): 11.58 (s, 2H, phenolic-OH), 8.31 (s, 2H, amidic-NH), 7.81 (s, 2H, CH=N), 7.56 (d, J= 8.7 Hz, 4H, Ar–H), 7.26 (two d, J= 6.7 Hz, 1.7 Hz, 3H, Ar-H), 6.91 (d, J= 8.4 Hz, 2H, Ar–H), 6.76 (t, J= 8.7 Hz, 5H, Ar–H), 3.88 (s, 2H, Ar–CH_2), 2.98 (s, 12H, Ar–$N(CH_3)_2$).

Figure S26: LC-MS spectrum of compound **5i**

Spectrum Plot Report

Name	SR-253	Rack Pos.		Instrument	APOGEE-LCMS	Operator	SYSTEM (SYSTEM)
Inj. Vol. (ul)	3	Plate Pos.		IRM Status			
Data File	20240408-Fast Scan-95 Org-SR-253b.d	Method (Acq)	20240404-Fast Scan-95 Org.m	Comment	DMSO-Heat-ACN-Filter-Dilute 100x in ACN	Acq. Time (Local)	2024-04-08 1:56:18 PM (UTC-07:00)

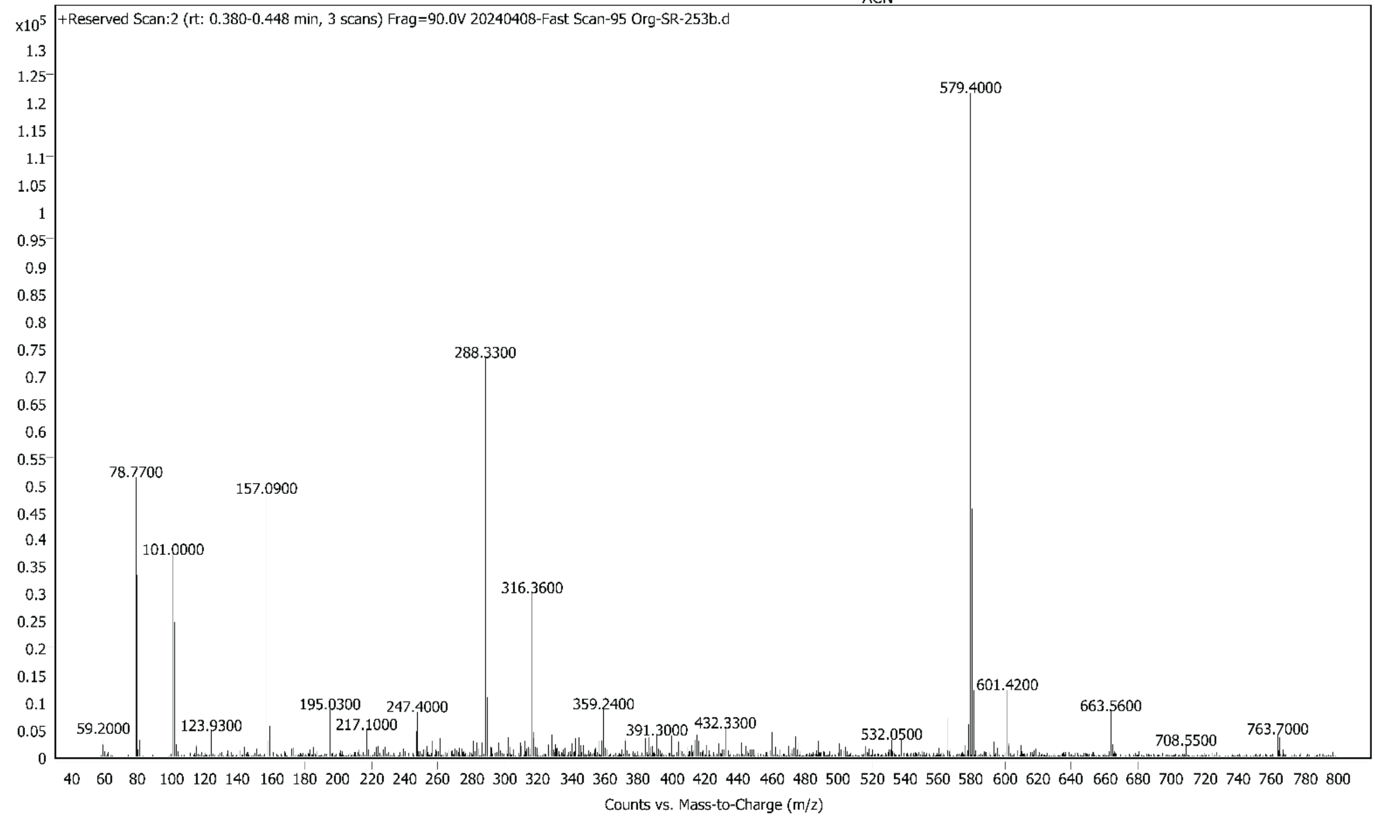

Figure S27: ^{1}H NMR spectrum (400 MHz, DMSO-d_6) of compound **5j**

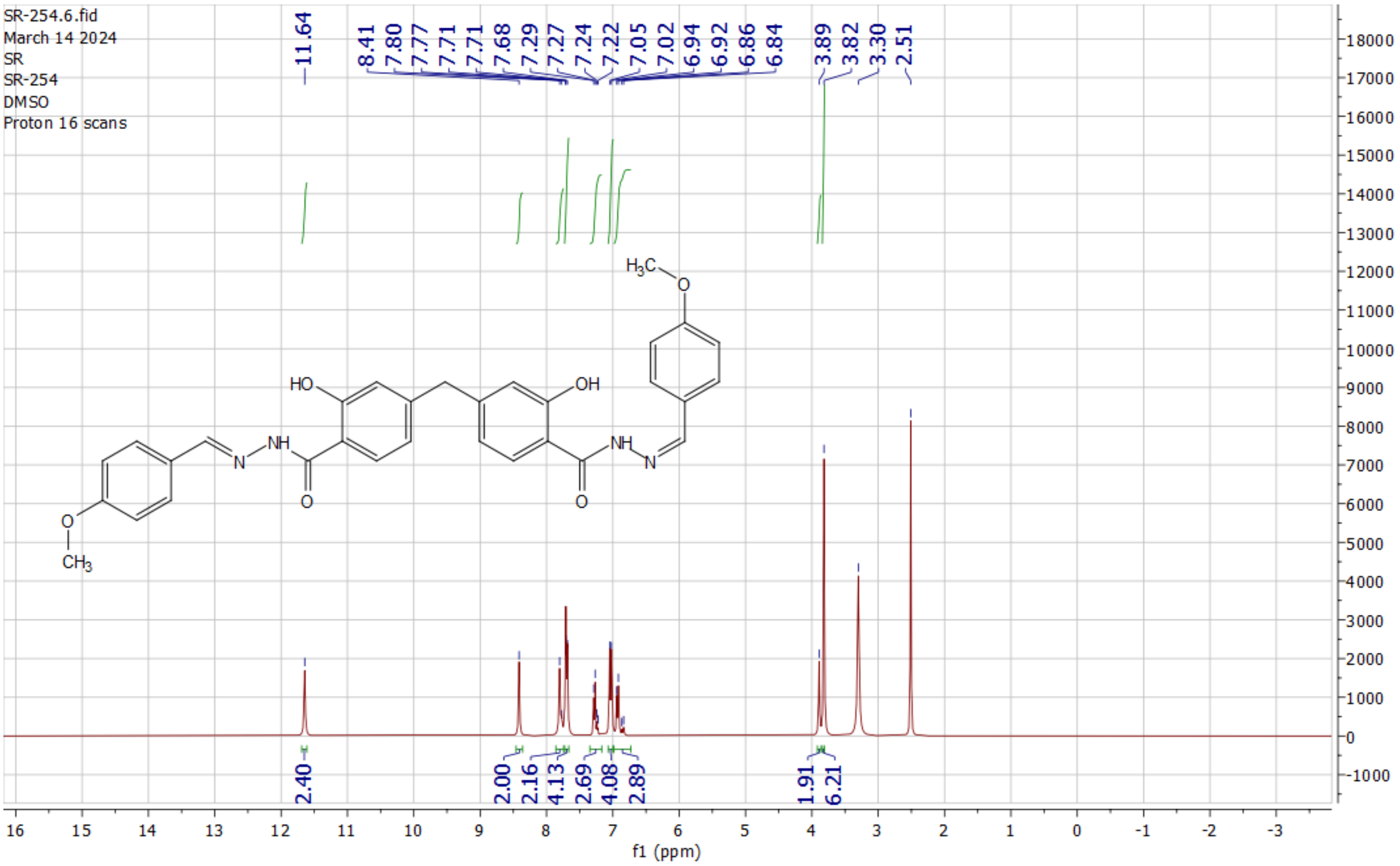

^{1}H NMR (400 MHz, δ ppm DMSO-d_6): 11.64 (s, 2H, phenolic-OH), 8.41 (s, 2H, amidic-NH), 7.80 (s, 2H, CH=N), 7.77-7.66 (m, 4H, Ar-H), 7.30-7.22 (m, 3H, Ar–H), 7.03 (d, J = 6.8 Hz, 4H, Ar-H), 6.92 (d, J = 7 Hz, 3H, Ar–H), 3.89 (s, 2H, Ar–CH_2), 3.82 (s, 6H, Ar–OCH_3).

Figure S28: LC-MS spectrum of compound **5j**

Spectrum Plot Report

Name	SR-254	Rack Pos.		Instrument	APOGEE-LCMS	Operator	SYSTEM (SYSTEM)
Inj. Vol. (ul)	3	Plate Pos.		IRM Status			
Data File	20240408-Fast Scan-95 Org-SR-254a.d	Method (Acq)	20240404-Fast Scan-95 Org.m	Comment	DMSO-Heat-ACN-Filter-Dilute 50x in ACN	Acq. Time (Local)	2024-04-08 2:56:59 PM (UTC-07:00)

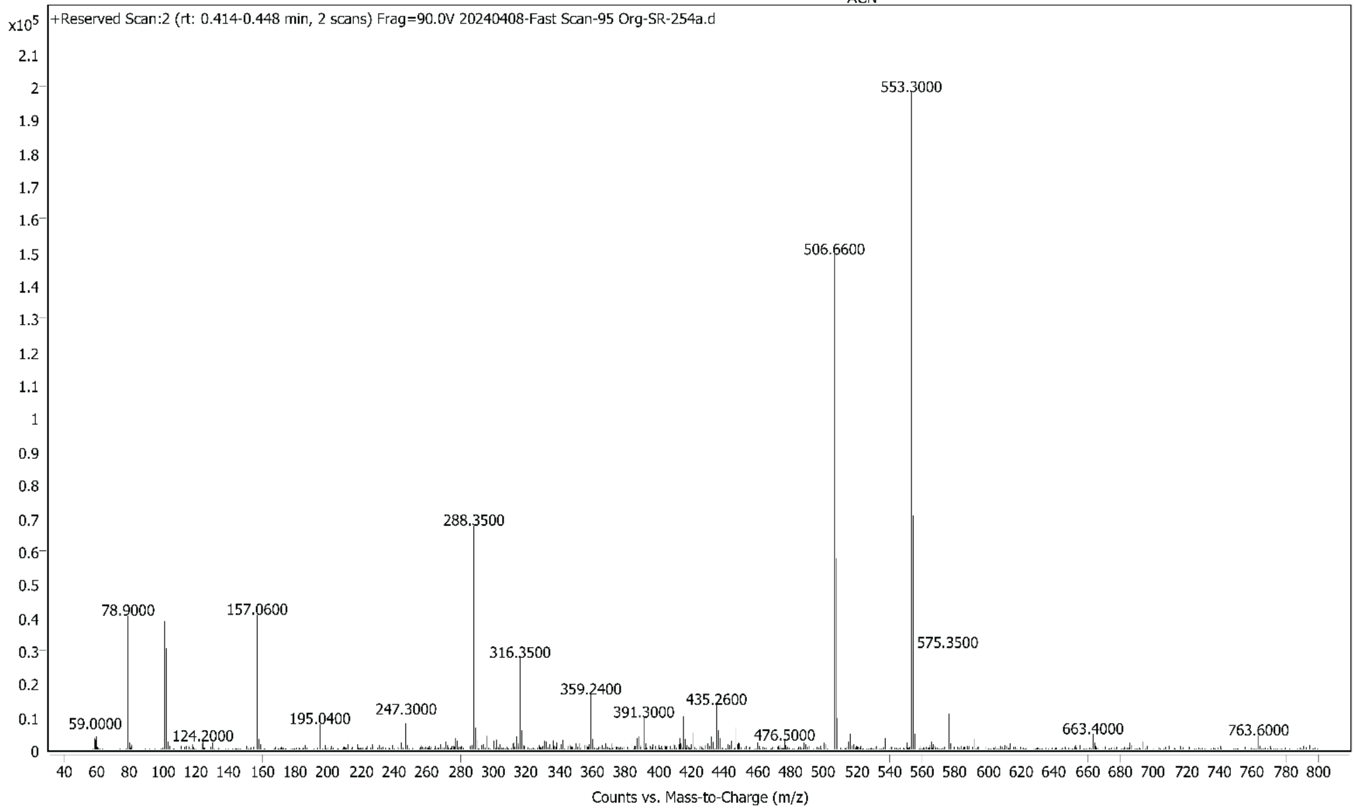

Figure S29: ^{1}H NMR spectrum (400 MHz, DMSO-d_6) of compound **5k**

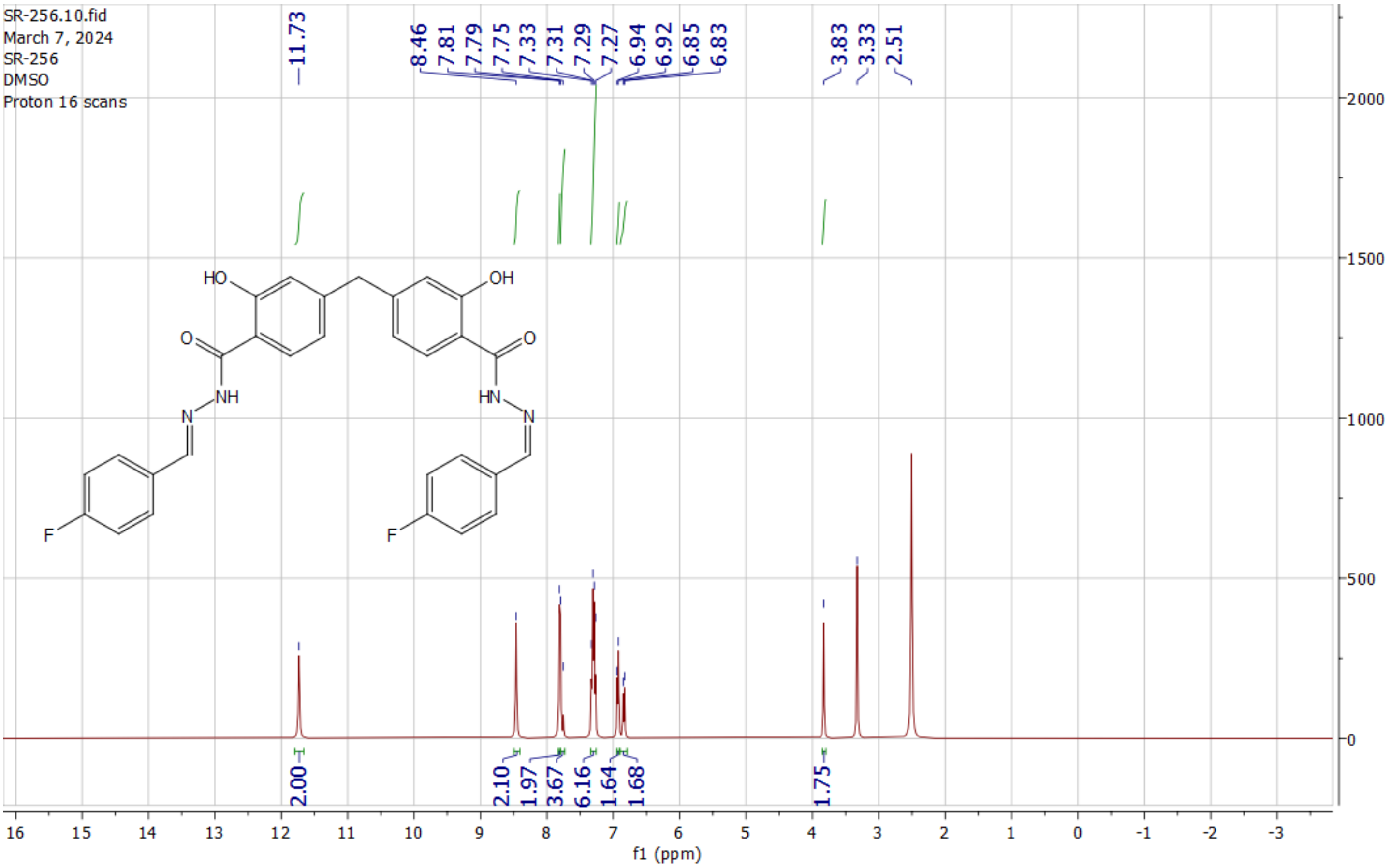

^{1}H NMR (400 MHz, δ ppm DMSO-d_6): 11.73 (s, 2H, phenolic-OH), 8.46 (s, 2H, amidic-NH), 7.81 (s, 2H, CH=N), 7.79-7.73 (m, 4H, Ar-H), 7.34-7.22 (m, 6H, Ar–H), 6.93 (d, J= 7.2 Hz, 2H, Ar-H), 6.84 (d, J= 8 Hz, 2H, Ar–H), 3.83 (s, 2H, Ar–CH_2).

Figure S30: LC-MS spectrum of compound **5k**

Spectrum Plot Report

Name	SR-256	Rack Pos.		Instrument	APOGEE-LCMS	Operator	SYSTEM (SYSTEM)
Inj. Vol. (ul)	3	Plate Pos.		IRM Status			
Data File	20240408-Fast Scan-95 Org-SR-256b.d	Method (Acq)	20240404-Fast Scan-95 Org.m	Comment	DMSO-Heat-ACN-Filter-Dilute 50x in ACN	Acq. Time (Local)	2024-04-08 2:31:34 PM (UTC-07:00)

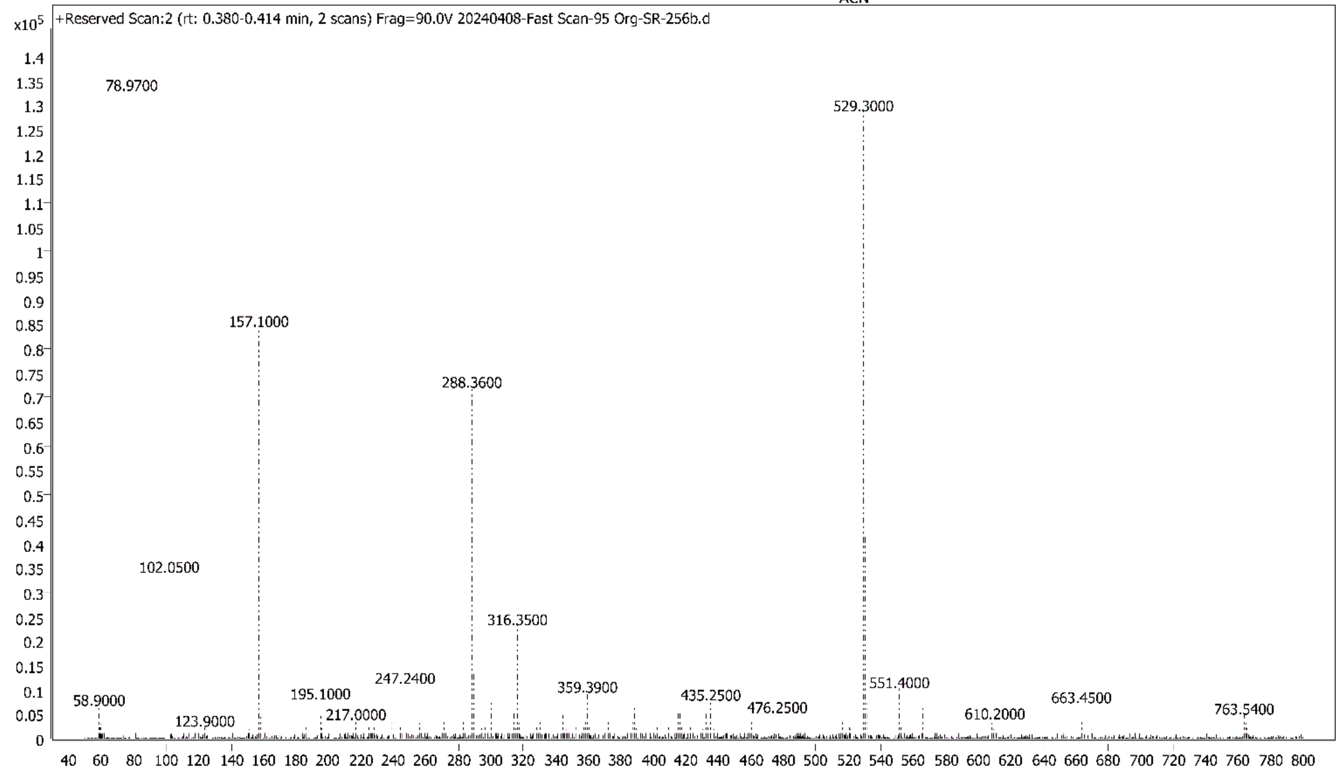

Counts vs. Mass-to-Charge (m/z)

Elemental Analysis sheet

جامعة الأزهر
Al-Azhar University
المركز الإقليمى للفطريات وتطبيقاتها
The Regional Center for Mycology and Biotechnology

Requester Data:

Name: **Dr. Bahaa Gamal Youssif**

Authority: Faculty of Pharmacy, Assuit University

Sample Data:

Eleven samples had been submitted for elemental analysis.

Analysis Report:

Sample Code	C%	H%	N%
5a	72.98	5.15	11.50
5b	62.25	4.09	10.25
5c	60.01	3.97	14.61
5d	53.78	3.54	8.89
5e	61.97	4.12	10.07
5f	71.34	5.66	10.97
5g	62.29	4.08	10.12
5h	67.51	5.23	10.41
5i	68.76	6.05	14.67
5j	67.25	5.07	10.35
5k	66.13	4.36	10.81

INVESTIGATOR M. M

DIRECTOR M. Mansour
4.4.2024

جامعة الأزهر
المركز الإقليمى للفطريات وتطبيقاتها

شارع المخيم الدائم - مدينة نصر - القاهرة
تليفون: ٢٢٦٢٠٣٧٣ (٠٢٠٢) فاكس: ٢٢٦٢٠٣٧٣ (٠٢٠٢)
البريد الإلكتروني: rcmb@azhar.edu.eg
الموقع الإلكتروني:
http:// www.azhar.edu.eg.htm
http://www.azhar.edu.eg/pages/fungi_center.htm
Facebook: RCMB AZHAR
صندوق بريد ١١٧٥١ مدينة نصر القاهرة

Appendix A

4. EXPERIMENTAL

4.1. Chemistry

General details:

All chemicals were purchased from Sigma Aldrich, Combi-Blocks, Fisher Scientific and they were used without purification unless mentioned. 1H NMR spectra were recorded in DMSOd-6 at 400 MHz on a Bruker AC 400 Ultrashield 10 spectrophotometer. Chemical shifts are expressed in ppm, (δ scale). When peak multiplicities are reported, the following abbreviations are used: s (singlet), d (doublet), m (multiplet), dd (doublet of doublet). Coupling constants are reported in Hertz (Hz). Low- and high-resolution mass spectra were recorded on a AB Sciex UHPLC/MS/MS System and a Thermo Scientific Q Exactive Orbitrap High Resolution Mass.

4.2. Antimicrobial activity

4.2.1. Organisms and culture conditions

The cultures used were collected from the Cairo University's Microanalytical Centre, Faculty of Science. An updated Kirby-Bauer disc diffusion method was applied for antimicrobial activities of the tested compounds [28]. Shortly, the 10 ml of fresh medium was grown to 100 μl bacteria / food until a count of 108 cell / ml or 105 cell / ml was achieved [29]. 100 μl microbial suspension has been spread over agar plates that suit the broth in which it was held. Selected colonies of each organism that may play a pathogenic function should be from the primary agar plates and tested by the disc diffusion method for susceptibility [30, 31]. Plates inoculated with filamentous fungi *as Aspergillus flavus* at 25°C for 48 hours; Gram positive bacteria as *Staphylococcus aureus* (ATCC 12600), *Bacillus subtilis* (ATCC 6051); Gram negative bacteria as *Escherichia coli* (ATCC 11775), *Pseudomonas aeuroginosa* (ATCC 10145) they were

incubated at 35-37°C for 24-48 hours and yeast as *Candida albicans* (ATCC 7102) incubated at 30°C for 24-48 hours and, then the diameters of the inhibition zones were measured in millimeters [28]. Standard discs of ciprofloxacin (Antibacterial agent), Fluconazole (Antifungal agent) served as positive controls for antimicrobial activity but filter discs impregnated with 10 µl of solvent (distilled water, chloroform, DMSO) were used as a negative control. Blank paper disks (Schleicher & Schuell, Spain) with a diameter of 8.0 mm were impregnated 10µ of tested concentration of the stock solutions. When a filter paper disc impregnated with a tested chemical is placed on agar the chemical will diffuse from the disc into the agar. This diffusion will place the chemical in the agar only around the disc. The solubility of the chemical and its molecular size will determine the size of the area of chemical infiltration around the disc. If an organism is placed on the agar it will not grow in the area around the disc if it is susceptible to the chemical. This area of no growth around the disc is known as a "Zone of inhibition" or" Clear zone". For the disc diffusion, the zone diameters were measured with slipping calipers of the National Committee for Clinical Laboratory Standards [30], and the results are given in **Table 1**. Agar-based methods such as E-test and disk diffusion can be good alternatives because they are simpler and faster than broth-based methods [30, 31].

4.2.2. Minimum inhibitory concentration assay

In 96-well microtiter plates and 50 mL of fresh bacterial culture of a single McFarland unit overnight, a double serial dilution of each compound (100 mL) in sterile standard saline were prepared to every single source well. Ciprofloxacin antibiotic (5 mg / mL-1) and normal saline were included as standard reference in each assay [32]. The plates were incubated at 37 ^{0}C overnight. As an indicator of bacterial growth, 40 mL of p-iodonitrotetrazolium violet (INT) was added to each well and incubated at 37 ^{0}C for 30

min. MIC values are recorded as the lowest concentration of the extract that completely inhibited bacterial growth that is clear well. The colorless tetrazolidium salt acts as an electron accepter and is reduced to a red colored formazan product by biological activity organisms. Where bacterial growth was inhibited, the solution in the well remained clear after incubation with INT. The observed MIC values are presented in **Table 2**.

4.2.3. Determination of Inhibitory Activities on *E. coli* DNA Gyrase and Topoisomerase IV.

All the final compounds were tested for *E. coli* DNA gyrase inhibitory activity in a supercoiling assay. Activities were determined on streptavidin-coated 96-well microtiter plates from Thermo scientific Pierce. First, the plates were rehydrated with buffer (20 mM Tris-HCl with pH 7.6, 0.01% w/v BSA, 0.05% v/v Tween 20, 137 mM NaCl) and the biotinylated oligonucleotide was then immobilized. After washing off the unbound oligonucleotide, the enzyme test was performed. The reaction volume of 30 μL in buffer (35 mM Tris-HCl with pH 7.5, 4 mM $MgCl_2$, 24 mM KCl, 2 mM DTT, 1.8 mM spermidine, 1 mM ATP, 6.5 % w/v glycerol, 0.1 mg/mL albumin) contained 1.5 U of DNA gyrase from *E. coli* or *S. aureus*, 0.75 μg of relaxed pNO1 plasmid, and 3 μL solution of the inhibitor in 10% DMSO and 0.008% Tween 20. Reaction solutions were incubated at 37 °C for 30 min. After that, the TF buffer (50 mM NaOAc with pH 5.0, 50 mM NaCl and 50 mM $MgCl_2$) was added to terminate the enzymatic reaction. After additional incubation for 30 min at rt, during which biotin-oligonucleotide-plasmid triplex was formed, the unbound plasmid was washed off using TF buffer and SybrGOLD in T10 buffer (10 mM Tris HCl with pH 8.0 and 1 mM EDTA) was added. The fluorescence was measured with a microplate reader (BioTek Synergy H4, excitation: 485 nm, emission: 535 nm). Initial screening was done at 100 or 10 μM concentration of inhibitors. For the most active inhibitors IC_{50} was determined using

seven concentrations of tested compounds. GraphPad Prism software was used to calculate the IC_{50} values. The result is given as the average value of three independent measurements. As the internal standard novobiocin (IC_{50} = 0.168 µM for *E. coli* gyrase and IC_{50} = 0.041 µM for *S. aureus* gyrase) was used. Determination of inhibitory activities on *E. coli* and *S. aureus* Topoisomerase IV. IC_{50} values were determined in an assay from In spiralis on streptavidin-coated 96-well microtiter plates from Thermo scientific Pierce. First, the plates were rehydrated with buffer (20 µM Tris-HCl with pH 7.6, 0.01% w/v BSA, 0.05% v/v Tween 20, 137 mM NaCl) and biotinylated oligonucleotide was then immobilized. After washing off the unbound oligonucleotide, the enzyme test was performed. The reaction volume of 30 µL in buffer (40 mM HEPES KOH with pH 7.6, 100 mM potassium glutamate, 10 mM magnesium acetate, 10 mM DTT, 1 mM ATP, 0.05 mg/mL albumin) contained 1.5 U of topoisomerase IV from *E. coli* or *S. aureus*, 0.75 µg of pNO1 supercoiled plasmid, and 3 µL solution of the inhibitor in DMSO (10%) and Tween 20 (0.008%). Reaction mixtures were incubated at 37 °C for 30 min and after that, the TF buffer (50 mM NaOAc with pH 5.0, 50 mM NaCl and 50 mM $MgCl_2$) was added to terminate the enzymatic reaction. After additional incubation for 30 min at rt, during which triplex (biotin-oligonucleotide-plasmid) was formed, the unbound plasmid was washed off using TF buffer and Sybr GOLD in T10 buffer (10 mM Tris HCl with pH 8.0 and 1 mM EDTA) was added. The fluorescence was measured with a microplate reader (BioTek Synergy H4, excitation: 485 nm, emission: 535 nm). Initial screening was done at 100 or 10 µM concentration of inhibitors. For the most active inhibitors IC_{50} was determined using seven concentrations of tested compounds. GraphPad Prism software was used to calculate the IC_{50} values. The result is given as the average value of three independent

measurements. As the internal standard novobiocin (IC_{50} = 11.1 µM) for *E. coli* topoisomerase IV and IC_{50} = 26.7 µM for *S. aureus* topoisomerase IV) was used.

4.2.3. Cell Viability assay

MTT assay was carried out to study the effect of compounds on mammary epithelial cells (MCF-10A). The medium in which cells were propagated contained Dulbecco's modified Eagle's medium (DMEM)/ Ham's F-12 medium (1:1) supplemented with epidermal growth factor (20 ng/mL), hydrocortisone (500 ng/mL), insulin (10 µg/mL), 2 mM glutamine and 10% fetal calf serum. After every 2-3 days, the cells were passaged using trypsin ethylenediamine tetra acetic acid (EDTA). The cells were seeded at a density of 10^4 cells mL^{-1} in flat-bottomed culture plates containing 96 wells each. After 24 h, medium was removed from the plates and the compounds in (in 0.1% DMSO) were added (in 200 µL medium to yield a final concentration of 0.1% v/v) to the wells of plates. A single compound was designated with four wells followed by incubation of plates for 96h at 37°C. After incubation, medium was removed completely from the plates followed by addition of MTT (0.4 mg/mL in medium) to each well and subsequent incubation of plates for 3h. MTT (along with the medium) was removed and DMSO (150µL) was added to each well of the culture plates, followed by vortexing and subsequent measurement of absorbance (at 540 nm) using microplate reader. The data are shown as percentage inhibition of proliferation in comparison with controls containing 0.1% DMSO.
